# How 3‑*O*‑Glucosylation Modulates Resveratrol Radical Scavenging: A QM-ORSA Study of Polydatin/Piceid, a Stilbenoid Glucoside

**DOI:** 10.1021/acs.jpcb.6c03897

**Published:** 2026-09-14

**Authors:** Maciej Spiegel, Nino Russo

**Affiliations:** † Department of Organic Chemistry and Pharmaceutical Technology, Faculty of Pharmacy, 49550 Wroclaw Medical University, Wroclaw 50-556, Poland; ‡ Department of Chemistry and Chemical Technologies, Università della Calabria, Rende 87036, Italy

## Abstract

The antioxidant properties of polydatin/piceid (3-*O*-β-*D*-glucopyranosyl-resveratrol), a stilbenoid polyphenol widely found in *Vitaceae*, *Liliaceae*, and *Leguminosae* plants and used in traditional medicine, have been investigated against the ^•^OOH radical by means of a density functional theory-based QM-ORSA protocol. The central mechanistic question addressed is how 3-*O*-glucosylation modulates the primary antiradical activity. To provide a biologically meaningful answer, its mechanisms of action were studied in both aqueous solution at physiological pH and in a lipid-like environment. Conformational analysis performed at the outset confirmed that the *trans* isomer is preferred over the *cis* form by 4.9 kcal·mol^–1^, and species distribution calculations show that the neutral form dominates at pH 7.4 (98.6%). The *para*-hydroxyl of ring B emerges as the dominant contributor to anti-^•^OOH activity regardless of the milieu; however, the activity trends show that while polydatin reacts faster than Trolox in the modeled lipid-like phase (9.78 × 10^3^ vs. 3.40 × 10^3^ M^–1^·s^–1^), in water its apparent rate constant (4.12 × 10^4^ M^–1^·s^–1^) falls below both *trans*-resveratrol (1.42 × 10^5^ M^–1^·s^–1^) and Trolox (8.96 × 10^4^ M^–1^·s^–1^).

## Graphical abstract

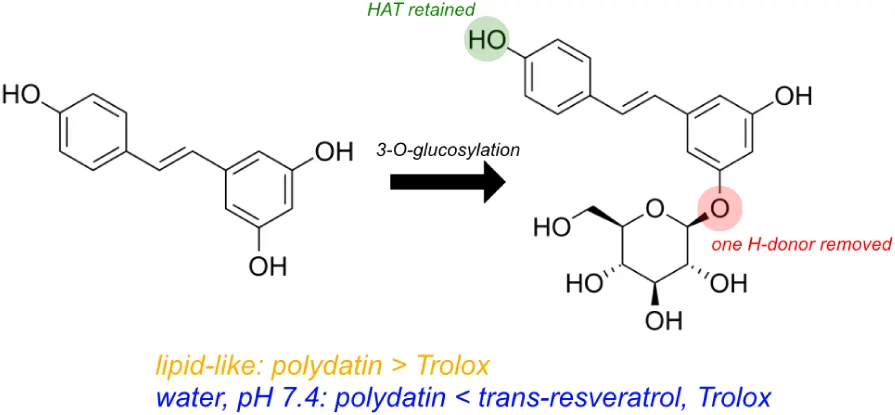

## Introduction

Polydatin, also known as piceid or 3-*O*-β-*D*-glucopyranosyl-resveratrol (Figure 1), is a stilbenoid polyphenol first isolated from the rhizome and root of *Polygonum cuspidatum*.^1^ Additional natural sources include plants of the *Vitaceae*, *Liliaceae*, and *Leguminosae* families.^2^ In traditional Chinese medicine, polydatin has long been used to treat inflammation, infections, and cardiovascular disorders. The substances has been also associated with antioxidant, anti-inflammatory, neuroprotective, hepatoprotective, immunostimulatory, and anticancer effects.^1,3–6^ Nonetheless, the scavenging power of polydatin relative to its aglycone is disputed: it has been found to exceed that of resveratrol in some experimental assays,^7–9^ whereas resveratrol shows greater activity in other assay systems, even within a single multi-assay study^9,10^ . Such discrepancies invite a mechanistic clarification especially given that polydatin, not resveratrol, is the major circulating stilbenoid following oral ingestion of plant-derived sources.^7,8^

**Figure 1:**
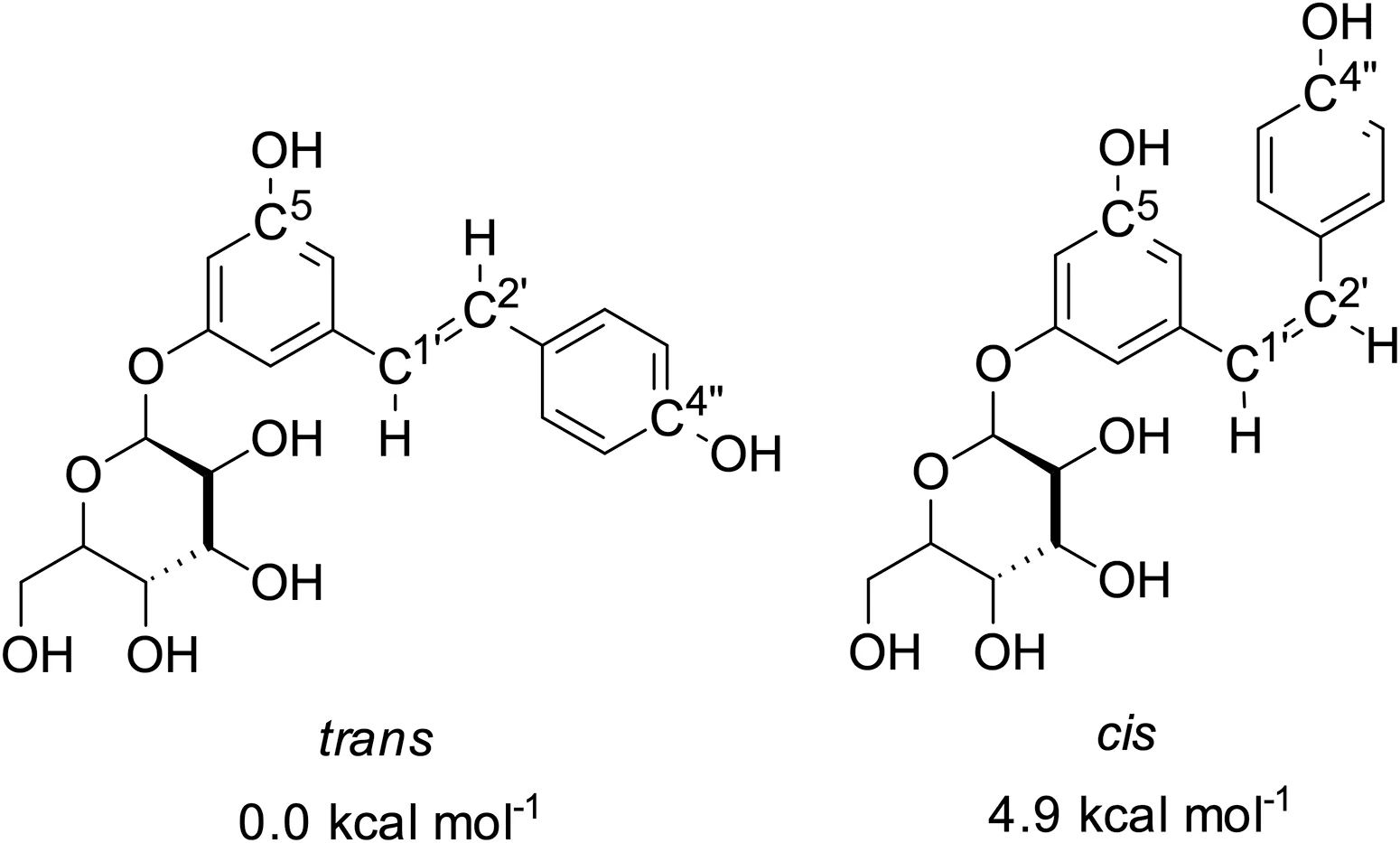
*Trans* and *cis* isomers of polydatin with atom numbering.

The chemistry of resveratrol and its derivatives has been extensively characterized by computational methods.^11–18^ From a structural perspective, polydatin differs from resveratrol solely by a β-d-glucopyranose unit attached at the C3 position of the stilbene backbone. One might assume, therefore, that polydatin’s antioxidant mechanism can be inferred by direct analogy to the parent compound; however, in our previous work on the related flavonoid glucosides vitexin and isovitexin, we demonstrated that glucosylation at an apparently unconnected motif relative to the main reactive part of the molecule may alter the overall radical-scavenging activity.^19^ Here the structure is smaller, and glycosylation directly masks one critical phenolic hydroxyl group, raising the question of whether and to what degree the polyhydroxylated sugar ring indirectly impacts the activity. Polydatin itself has not previously been characterized at this level of detail and the present work addresses this gap by comparing the calculated radical-scavenging behavior of polydatin with that of *trans*-resveratrol, providing for the first time systematic kinetic data for this stilbenoid glucoside.

We used density functional theory within a dedicated thermochemistry-kinetic framework, previously applied to a series of natural antioxidants,^20–24^ to examine the structural and electronic properties of polydatin and its Hydrogen Atom Transfer (HAT), Single Electron Transfer (SET), and Radical Adduct Formation (RAF) reactions with ^•^OOH in aqueous and lipid-like environments. The computed rate constants were then compared with those of Trolox, *trans*-resveratrol, and selected natural and non-natural antioxidants to contextualize the effect of 3-*O*-glucosylation on the radical-scavenging profile of the stilbene scaffold.

### Computational Details

Since unambiguous structural parameters for polydatin in its ground-state conformation are not established in the literature, initial geometries were obtained *via* metadynamics conformational sampling as implemented in the CREST software.^25^ The resulting structures were subjected to full geometry optimization using the M05–2X exchange-correlation meta-GGA functional^26^ combined with the 6–311+G­(d,p) basis set^27,28^ and the SMD implicit solvation model.^29^ Frequency calculations were performed at the same level of theory to confirm that the optimized structures correspond to true minima (ground states) or first-order saddle points (transition states). Open-shell species were treated using the unrestricted formalism. Intrinsic reaction coordinate calculations were carried out for each transition state to confirm proper connectivity between reactants and products. All calculations were carried out using Gaussian 16 (Rev. C.01).^30^

Pentyl ethanoate (ε = 4.7) and water (ε = 78.4) environments were used to simulate lipid-like environments and aqueous conditions, respectively. The p*K*_a_ values and molar fractions in aqueous phase at physiological pH (7.4) were obtained using an empirically fitted parameters method^31^ to account for the different protonation states of the same molecule.

Global reactivity indices—bond dissociation energies (BDE) and ionization potentials (IP)—were estimated within the adiabatic approximation using the solvation enthalpies of H^+^ (Δ*H*(H^+^) = 252.3 kcal·mol^–1^) and the electron (Δ*H*(e^–^) = 18.5 kcal·mol^–1^).^32^

Rate constants were calculated within the framework of the quantum mechanics-based computational protocol for overall free radical scavenging activity (QM-ORSA) approach,^33^ based on conventional Transition State Theory:

$$k=\sigma \kappa \frac{k_{B}T}{h}e^{\left(-\frac{\Delta G^{‡}}{RT}\right)}$$

where σ and κ represent the reaction path degeneracy and tunneling correction (Eckart model), respectively; *k*_B_, *h*, and *R* are the Boltzmann, Planck, and gas constants; and Δ*G* ^*‡*^ is the Gibbs free energy of activation at temperature *T*. For SET mechanisms, activation free energies were estimated using Marcus theory:^34^

$$\Delta G^{‡}=\frac{\lambda }{4}{\left(1+\frac{\Delta G}{\lambda }\right)}^{2}$$

where λ is the reorganization energy, computed using the ΔSCF method. For reactions approaching the diffusion limit, the apparent rate constant was calculated *via* the Collins–Kimball expression:^35^

$$k_{app}=\frac{k_{D}k}{k_{D}+k}$$

where *k* is the TST thermal rate constant and *k*_D_ is the Smoluchowski steady-state diffusion-controlled rate constant.^36^ All energies were corrected to the 1 M standard state and for solvent cage effects using Okuno’s corrections with Benson’s free volume theory^37^ prior to substitution into the expression used to calculate the second-order rate constants.

## Results and Discussion

### Conformational Preference and Structural Overview

Polydatin can adopt two distinct conformations depending on the orientation of the stilbene double bond. As shown in Figure 1, the *trans* form is more stable than the *cis* isomer by 4.9 kcal·mol^–1^ (in aqueous solution), which, according to a Boltzmann distribution at 298 K, corresponds to a population of the *cis* form of approximately 0.025%. All subsequent calculations were therefore conducted exclusively on the *trans* isomer. This conformational preference mirrors that observed for resveratrol.^38^

### Acid–Base Behavior and Species Distribution

Given that polydatin contains two free phenolic hydroxyl groups capable of deprotonation, it can exist as neutral, monoanionic, and dianionic species in aqueous solution, with each protonation state contributing differently to overall antioxidant activity. The order of deprotonation of these groups is not *a priori* evident. Therefore, we first investigated its acid–base behavior to determine the distribution of protonation states at physiological pH—experimental studies report only the first dissociation constant (9.40 ^39^ and 9.22 ^40^), whereas our calculations provide both p*K*_a_ values. The deprotonation pathway and the corresponding molar fraction distributions are shown in Figure 2.

**Figure 2:**
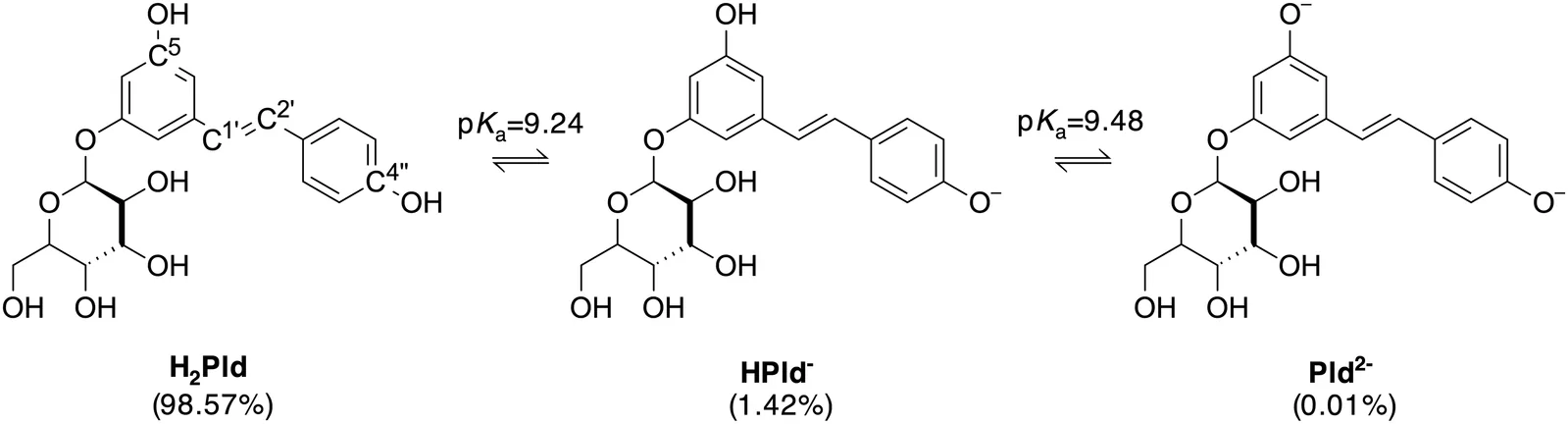
Deprotonation pathways and molar fraction distributions of polydatin as a function of pH.

The first deprotonation involves the hydroxyl group at C4”, with a computed p*K*_a_ of 9.24, in good agreement with the above-mentioned literature data. The subsequent deprotonation occurs at C5, yielding p*K*_a_ = 9.48. Both values lie well above physiological pH, so at pH 7.4 the neutral form **H**_**2**_ **Pld** dominates overwhelmingly (98.57%), followed by the monoanionic form **HPld** ^–^ (1.42%). The dianionic species **Pld** ^**2**–^ accounts for only 0.01% and hence is not included in the calculations.

### Global Reactivity Descriptors

The reactivity indices in Table 1 reveal a mechanistically important consequence of 3-*O*-glucosylation. With the C3-hydroxyl of ring A blocked by the glycosidic bond, the molecule retains two free phenolic groups whose relative lability governs which site should dominate radical scavenging. Consistent with prior computational studies on *trans*-resveratrol that have established the role of *para*-hydroxyl of the ring B^11,12^ (here denoted as C4”), this group also bears the weakest O–H bond in both environments (BDE = 89.2 kcal·mol^–1^ in lipid, 90.2 kcal·mol^–1^ in water), implying it as the thermodynamically preferred HAT site. The C5 group is a weaker H-donor (BDE = 95.5 kcal·mol^–1^ in lipid, 97.5 kcal·mol^–1^ in water), though its reactivity increases substantially in the anionic species **HPld** ^–^ (BDE = 89.4 kcal·mol^–1^). This activating effect can be associated with the increased electron density propagated through the conjugated π-system from the deprotonated C4” group. The remaining possible sites of H atom abstraction are vinyl C–H positions (C1’, C2’), which exhibit BDE values exceeding 110 kcal·mol^–1^ in all environments, confirming they are not competitive HAT donors. The adiabatic ionization potential decreases from the lipid-like phase (122.2 kcal·mol^–1^) to aqueous solution (104.1 kcal·mol^–1^), reflecting enhanced charge stabilization by the polar environment, and decreases further for **HPld** ^–^ (82.8 kcal·mol^–1^), consistent with increased electron-donating character upon deprotonation.

**Table 1:** O–H Bond Dissociation Energies (BDE) and Adiabatic Ionization Potentials (aIP) in Lipid-Like and Aqueous Environments^a^

		H_2_Pld_lipid_	H_2_Pld_aq_	HPld^–^_aq_
**BDE**	C5	95.5	97.5	89.4
	C1’	111.2	113.0	113.5
	C2’	110.8	112.5	110.9
	C4”	89.2	90.2	
**aIP**		122.2	104.1	82.8

a All values in kcal·mol^–1^.

### Thermodynamics and Kinetics of Radical Scavenging

Intrinsic reactivity indices alone cannot discriminate among competing mechanisms but only guide the analysis. The antioxidant properties of polydatin were therefore determined by means of the QM-ORSA protocol.

The Gibbs free energies for the reaction between the ^•^OOH radical and the polydatin species are collected in Table 2. The HAT mechanism at C4” is thermodynamically most favored in both lipid (ΔG = −2.4 kcal·mol^–1^) and aqueous (ΔG = −5.3 kcal·mol^–1^) media, reflecting the low BDE of this hydroxyl group. The C5 site is also thermodynamically accessible to HAT in aqueous solution (ΔG = 2.3 kcal·mol^–1^) and becomes exergonic for the monoanionic species (ΔG = −5.9 kcal·mol^–1^), rationalizing the high reactivity of **HPld^–^** as discussed below.

**Table 2:** Gibbs Free Energies of Reaction (ΔG) for HAT, RAF, and SET Mechanisms Between Polydatin Species and the ^•^OOH Radical.^a^

		H_2_Pld_lipid_	H_2_Pld_aq_	HPld^–^_aq_
**HAT**	C5	3.9	2.3	–5.9
	C1’	17.7	16.5	16.4
	C2’	19.2	16.4	14.5
	C4”	–2.4	–5.3	
**RAF**	C1	21.5	20.0	20.2
	C2	10.2	7.7	7.2
	C3	16.1	13.6	13.5
	C4	8.9	6.1	6.2
	C5	16.1	14.8	14.7
	C6	9.7	10.4	9.9
	C1’	–2.5	–3.5	–4.9
	C2’	–0.1	–1.7	–2.0
	C1”	19.0	17.0	12.6
	C2”	11.9	9.7	10.2
	C3”	16.2	15.4	7.8
	C4”	8.0	6.2	7.7
	C5”	15.3	14.9	7.1
	C6”	13.5	11.7	11.6
**SET**			23.3	0.5

a All values in kcal·mol^–1^.

Among the RAF pathways, adduct formation at C1’ and C2’ yields slightly negative ΔG values (−2.5 and −0.1 kcal·mol^–1^ in lipid, respectively), indicating thermodynamic feasibility. Of note, these pathways become more exergonic upon conversion of **H_2_Pld** to **HPld** ^–^ and upon changing the solvent from lipid to water. RAF on the non-glycosylated ring carbons (C1”–C6”) is generally endergonic, but several positions show modest positive ΔG values (6–15 kcal·mol^–1^) that do not preclude kinetic participation, particularly for the monoanionic species. This confirms that the stilbenoid π-system remains chemically accessible to RAF pathways beyond the phenolic HAT sites.

SET is strongly endergonic for neutral polydatin in aqueous solution (23.3 kcal·mol^–1^), and it becomes near-thermoneutral for the monoanionic species (0.5 kcal·mol^–1^). Such behavior is consistent with the high aIP of **H**_**2**_ **Pld**_**aq**_ and with the general observation that SET processes are significant mainly for deprotonated polyphenol species, where electron-donating ability is substantially enhanced. In this regard, although the SET pathway appears relevant primarily for the monoanionic species, a strongly endergonic free energy change does not in itself rule out electron transfer, since within the framework of Marcus theory the reaction rate depends on both ΔG and the reorganization energy.

For the exergonic and mildly endergonic reactions (ΔG ≤ 10.0 kcal·mol^–1^), the activation free energies were determined (Table 3). The results confirm the hierarchy established by the thermodynamic analysis while revealing important medium- and speciation-dependent nuances: in the lipid-like environment, the lowest barrier (ΔG^‡^ = 14.6 kcal·mol^–1^) is associated with HAT at C4”, identifying this as the kinetically preferred route, consistent with its lowest BDE. In aqueous solution the HAT barrier at C4” rises modestly to 18.0 kcal·mol^–1^, while RAF at C1’ becomes increasingly competitive (ΔG^‡^ = 13.7 kcal·mol^–1^ for **H**_**2**_ **Pld**, falling to 8.3 kcal·mol^–1^ for **HPld** ^–^) demonstrating stabilization of the transition state for radical addition in water. A similar, though less pronounced, trend is observed for C2’. The SET barrier for the neutral form (23.3 kcal·mol^–1^) effectively renders this pathway inoperative, but it drops substantially for **HPld** ^–^ (6.4 kcal·mol^–1^), with both processes characterized by a reorganization energy of λ = 24.5 kcal·mol^–1^. This indicates that the dramatic kinetic enhancement observed upon deprotonation is driven strictly by thermodynamics, rather than changes in structural or solvent cage reorganization costs, reflecting the rigid π-conjugated nature of the polydatin scaffold and the localized charge redistribution upon electron transfer.

**Table 3:** Activation Free Energies (ΔG^‡^) for Feasible HAT, RAF, and SET Mechanisms.^a^ ^b^

ΔG^‡^		H_2_Pld_lipid_	H_2_Pld_aq_	HPld^–^_aq_
HAT	C5	17.7	18.3	b.l.^c^
	C4”	14.6	18.0	
RAF	C2		16.0	14.7
	C4	20.9	17.5	15.7
	C6	19.7		16.6
	C1’	15.8	13.7	8.3
	C2’	16.2	13.8	12.0
	C2”		19.9	
	C3”			12.7
	C4”	23.3	18.9	10.5
	C5”			12.8
SET			23.3	6.4

a All values in kcal·mol^–1^.

b All values are calculated relative to infinitely separated reactants, and include Okuno’s solvent cage correction alongside the 1 M standard state correction.

c b.l. = barrierless reaction.

The optimized transition state structures for the HAT and RAF pathways are shown in Figure 3. Analysis of the single imaginary frequency and the corresponding IRC profiles for the representative TSs (see Supporting Information) confirms correct connectivity between reactants and products.

**Figure 3:**
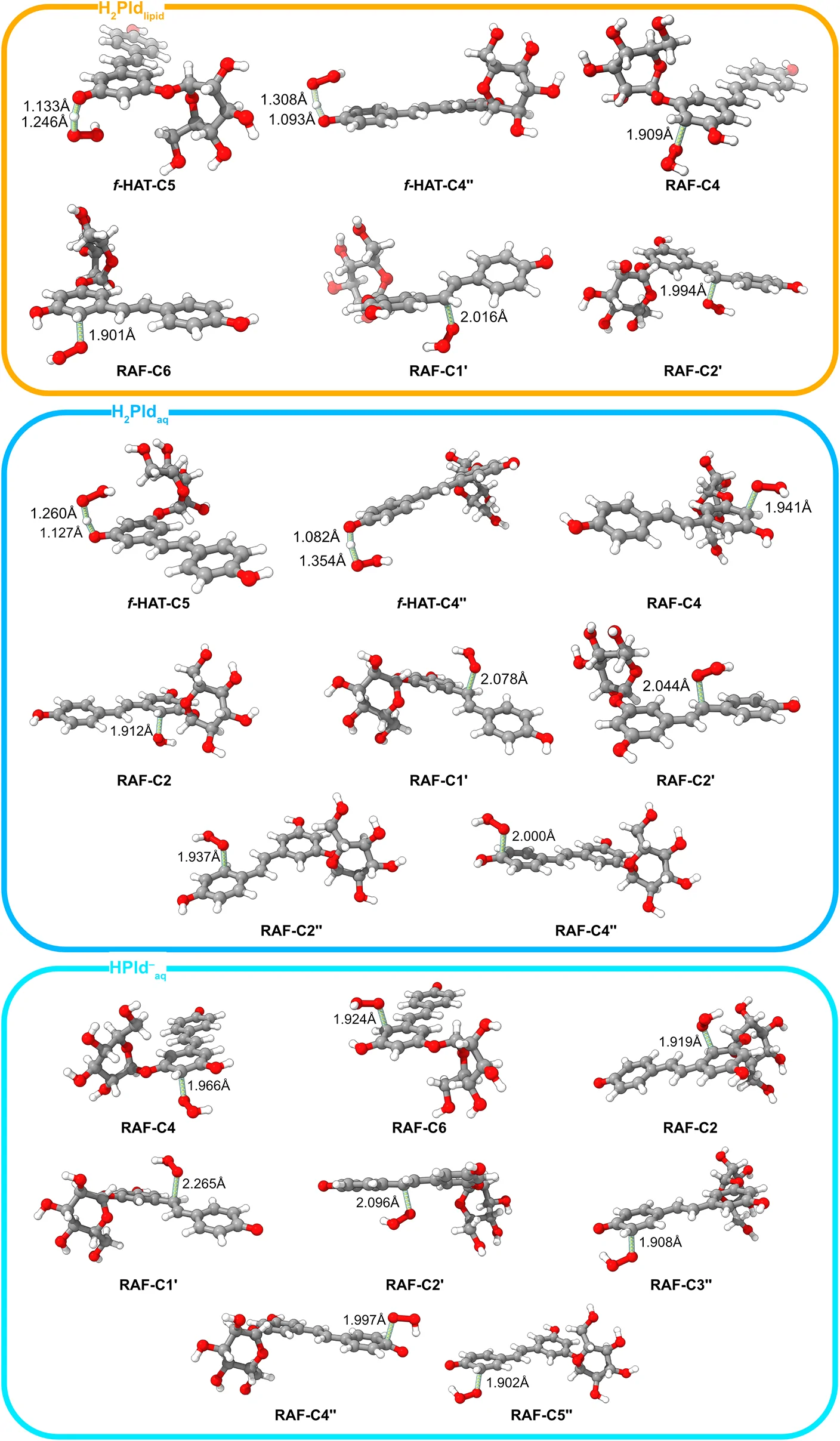
Optimized transition state structures for HAT and RAF reactions of polydatin with ^•^OOH.

The computed rate constants and branching ratios are collected in Table 4. In the lipid-like environment, HAT at C4” dominates overwhelmingly, accounting for 98.93% of the total rate constant (9.67 × 10^3^ M^–1^·s^–1^), with minor contributions from C5-HAT (0.71%) and vinyl RAF (C1’ 0.25% at C1’ and 0.12% at C2’). In aqueous solution, the neutral form **H**_**2**_ **Pld** still reacts predominantly via HAT at C4” (72.76%) and C5 (5.76%), but the RAF pathways at C1’ and C2’ account for a non-negligible 11.25% and 10.00%, respectively—a noteworthy departure from the lipid-phase behavior.

**Table 4:** Rate Constants (*k*, M^–1^·s^–1^)­^a^ and Branching Ratios (Γ, %) for Polydatin Species Reacting with the ^•^OOH Radical

		H_2_Pld_lipid_	H_2_Pld_aq_	HPld^–^_aq_
HAT	C5	6.93×10^1^ (0.71)	3.86×10^2^ (5.76)	1.02×10^9^ (88.03)^b^
	C4”	9.67×10^3^ (98.93)	4.87×10^3^ (72.76)	
RAF	C2		1.36×10^1^ (0.20)	1.16×10^2^ (0.00)
	C4	3.10×10^–2^ (0.00)		5.29×10° (0.00)
	C6	4.54×10^–3 (^0.00)	1.25×10° (0.02)	2.39×10^1^ (0.00)
	C1’	2.40×10^1^ (0.25)	7.53×10^2^ (11.25)	6.72×10^6^ (0.58)
	C2’	1.16×10^1^ (0.12)	6.70×10^2^ (10.00)	1.22×10^4^ (0.00)
	C2”		2.57×10^–2^ (0.00)	
	C3”			3.21×10^3^ (0.00)
	C4”		1.44×10^–1^ (0.00)	1.43×10^5^ (0.01)
	C5”			2.67×10^3^ (0.00)
SET			5.15×10^–5^ (0.00)	1.32×10^8^ (11.38)
*k*_total_ ^c^		9.78×10^3^	6.70×10^3^	1.16×10^9^
*k*_overall_ ^c^			6.60×10^3^	1.64×10^7^
*k*_apparent_ ^c^			4.12 ×10^4^	

a All rate constants were calculated using activation free energies corrected for the 1 M standard state and Okuno’s solvent cage effect. For diffusion-controlled reactions, apparent rate constants were calculated via the Collins–Kimball model.

b Reaction approaching the diffusion-controlled limit.

c *k*_total_ = Σ*k*_*i*_ for a specific species; *k*_overall_ = *k*_total_ × α_*i*_ (α_*i*_ = molar fraction at pH 7.4); *k*_apparent_ = Σ*k*_overall_ × Φ_OOH_, where Φ_OOH_ = 0.0025 (molar fraction of ^•^OOH at pH 7.4).

The monoanionic species **HPld** ^–^ (molar fraction 1.42% at pH 7.4) is markedly more reactive, with a total rate constant of 1.16 × 10^9^ M^–1^·s^–1^, dominated by barrierless HAT at C5 (88.03%), which approaches the diffusion-controlled limit. Despite constituting less than 1.5% of the speciation at pH 7.4, **HPld** ^–^ contributes disproportionately to the apparent rate constant, a pattern documented previously also for other types of polyphenolic anions.^20,41^

### Comparison with Reference Antioxidants and the Effect of 3-*O*-Glucosylation

The agreement between the computed first p*K*_a_ and two independent experimental datasets, together with the well-benchmarked QM-ORSA protocol, lends confidence to the kinetic outcomes. The computed apparent rate constant for polydatin (*k*_app_ = 4.12 × 10^4^ M^–1^·s^–1^) can therefore be contextualized against a set of small antioxidants and reference compounds previously investigated. The medium changes the comparison decisively (Figure 4)—in the lipid-like environment, the reactivity trend is

**Figure 4:**
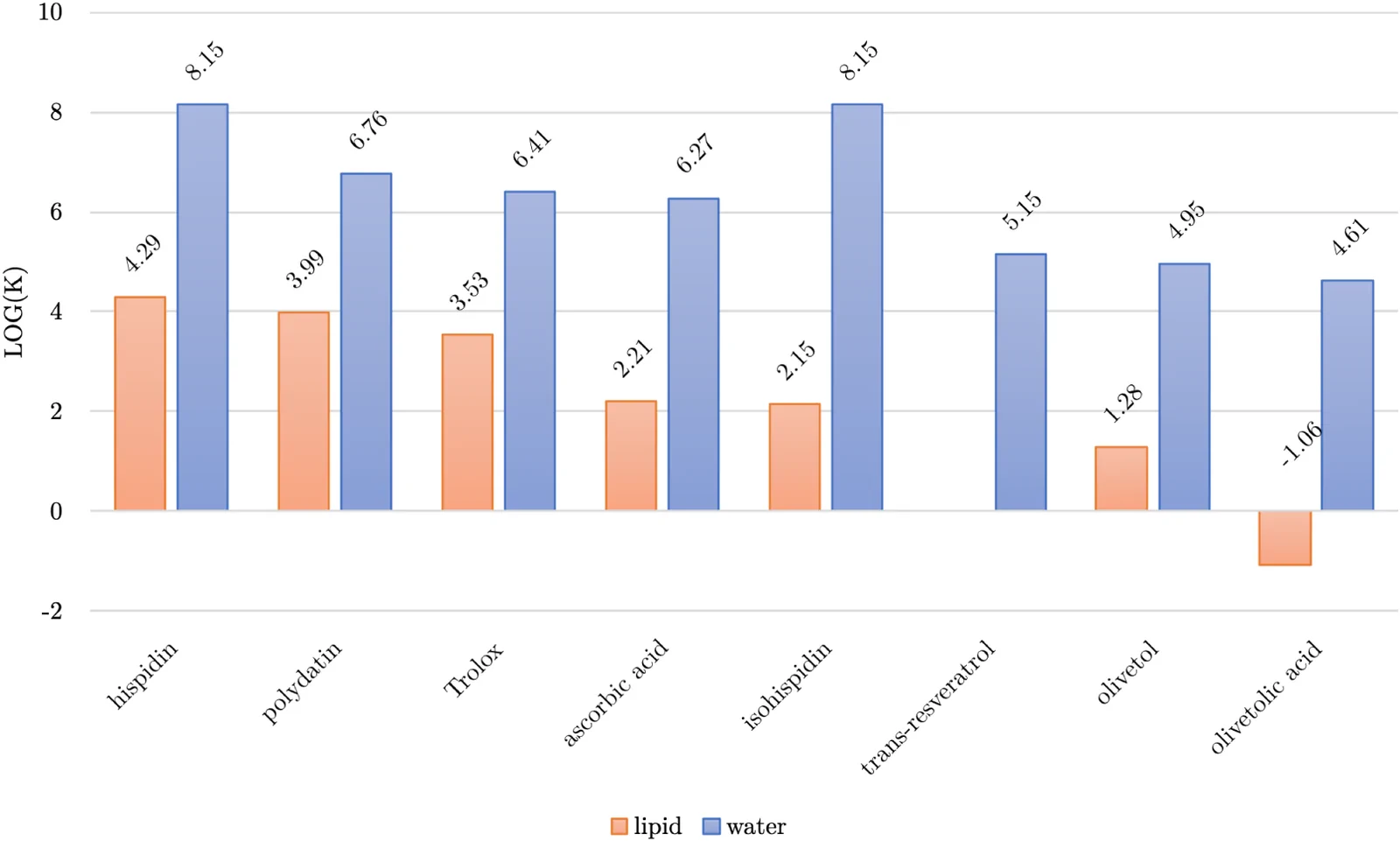
Comparison of ^•^OOH-scavenging rate constants (log scale) for selected antioxidants in lipid-like (pentyl ethanoate) and aqueous environments.

hispidin (1.94 × 10^4^)^42^ > polydatin (9.78 × 10^3^) > Trolox (3.40 × 10^3^)^43^ > ascorbic acid (1.63 × 10^2^)^44^ > isohispidin (1.40 × 10^2^)^42^ > olivetol (1.92 × 10^1^)^24^ > olivetolic acid (8.70 × 10^–2^)^24^

whereas in aqueous solution it becomes

isohispidin/hispidin (1.40 × 10^8^) > olivetol (5.76 × 10^6^) > ascorbic acid (2.56 × 10^6^) > olivetolic acid (1.87 × 10^6^) > *trans*-resveratrol (1.42 × 10^5^)^11^ > Trolox (8.96 × 10^4^) > polydatin (4.12 × 10^4^).

In lipid, glycosylation does not suppress antioxidant activity relative to Trolox, and the C4” hydroxyl remains the most labile H-donor, producing a rate constant 2.9-fold higher than Trolox and approximately 60-fold higher than ascorbic acid. Hence, in this environment, polydatin is a competent lipid-phase antioxidant.

In water at physiological pH, the picture reverses relative to resveratrol, and polydatin’s apparent rate constant falls approximately 3.4-fold below *trans*-resveratrol and 2.2-fold below Trolox. The mechanistic origin is unambiguous and can be associated with the C3-involving pathway that drives resveratrol’s aqueous reactivity is blocked in polydatin. While the monoanionic **HPld** ^–^ is intrinsically highly reactive, its low molar fraction at pH 7.4 limits its contribution to the apparent rate constant.

As a final remark, we emphasize that experimental observations in which polydatin appears comparable or superior to resveratrol should therefore not be interpreted as a direct consequence of higher intrinsic ^•^OOH- scavenging reactivity. The present calculations instead suggest that such differences may arise from biological or physicochemical factors, including solubility, membrane partitioning, metabolic stability, or assay-specific radical chemistry.^9,10,45^ In this regard, further studies on the antioxidant metabolites predominating in the living organism are clearly warranted.

## Conclusions

The primary antioxidant properties of polydatin against the ^•^OOH radical have been studied using the QM-ORSA density functional theory protocol. Overall, the calculations indicate that 3-*O*-glucosylation changes but does not suppress the primary radical-scavenging chemistry of the resveratrol scaffold, yet visibly alters it. The key findings are summarized as follows:

(i) The *trans* isomer is more stable than the *cis* form by 4.9 kcal·mol^–1^. At physiological pH the neutral **H**_**2**_ **Pld** species dominates (98.6%).
(ii) Stepwise deprotonation is predicted to occur at C4” (p*K*_a_ = 9.24), followed by C5 (p*K*_a_ = 9.48), both well above physiological pH. The first p*K*_a_ is in good agreement with experimental values, lending confidence to the approach.
(iii) In both lipid-like and aqueous environments the HAT mechanism is the preferred antioxidant pathway, with the C4” hydroxyl as the dominant reactive site. RAF pathways at C1’ and C2’ contribute to radical scavenging in aqueous solution, for the neutral species, while SET becomes significant only for **HPld** ^–^.
(iv) Relative to the reference compounds considered here, polydatin performs more favorably in the lipid-like medium than in water. In pentyl ethanoate, its rate constant is approximately 2.9-fold higher than Trolox and about 60-fold higher than ascorbic acid. In water, its apparent rate constant is approximately 2.2-fold lower than Trolox, 3.4-fold lower than *trans*-resveratrol, and about 62-fold lower than ascorbic acid.
(v) The antioxidant behavior of polydatin relative to resveratrol is medium-dependent. In lipid, the persistence of the reactive C4” hydroxyl ensures competitive HAT despite glycosylation at C3. In water, the attenuation relative to resveratrol is explained by the loss of the C3-phenolate HAT pathway, which is permanently blocked by the glycosidic bond.

## Supplementary Material



## Data Availability

The data underlying this study are available in the published article and its Supporting Information.

## Notes

Cartesian coordinates, electronic energies, thermal energies, enthalpies, and free energies (PDF)
