# Supplementary material for: How 3‑O‑Glucosylation Modulates Resveratrol Radical Scavenging: A QM-ORSA Study of Polydatin/Piceid, a Stilbenoid Glucoside
Source: J Phys Chem B. 2026 Sep 14;130(38):9981–8. doi: 10.1021/acs.jpcb.6c03897 (PMC13618030; doi:10.1021/acs.jpcb.6c03897)
Supplement: Supplementary file 1 [file jp6c03897_si_001.pdf]

How 3-*O*-Glucosylation Modulates Resveratrol Radical Scavenging: A QM-ORSA Study of Polydatin/Piceid, a Stilbenoid Glucoside

Maciej Spiegel[a,b,*] and Nino Russo[b]

[a] Department of Organic Chemistry and Pharmaceutical Technology, Faculty of Pharmacy, Wroclaw Medical University, Wroclaw, Poland; e-mail: maciej.spiegel@umw.edu.pl

[b] Department of Chemistry and Chemical Technologies, Università della Calabria, Rende, Italy

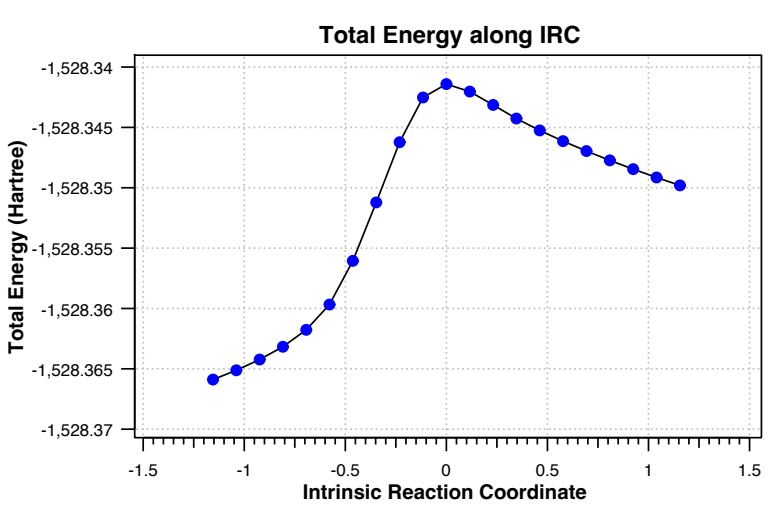

H_2Pld_{lipid} – HAT@C4”

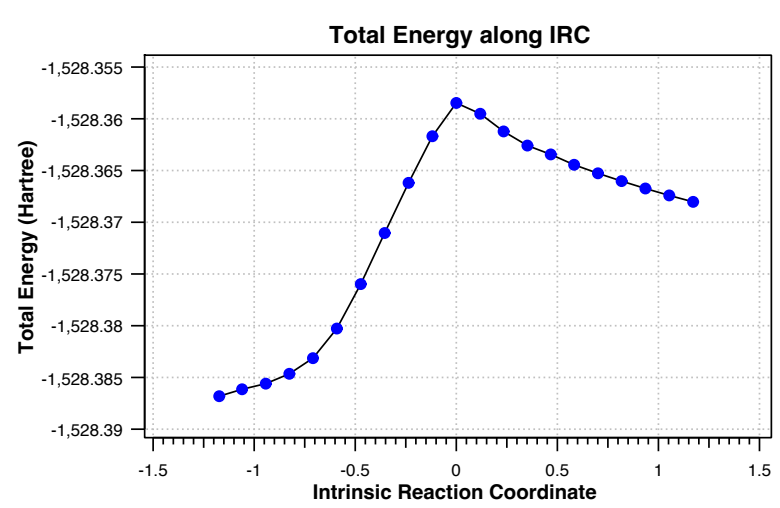

H_2Pld_{aq} – HAT@C4”

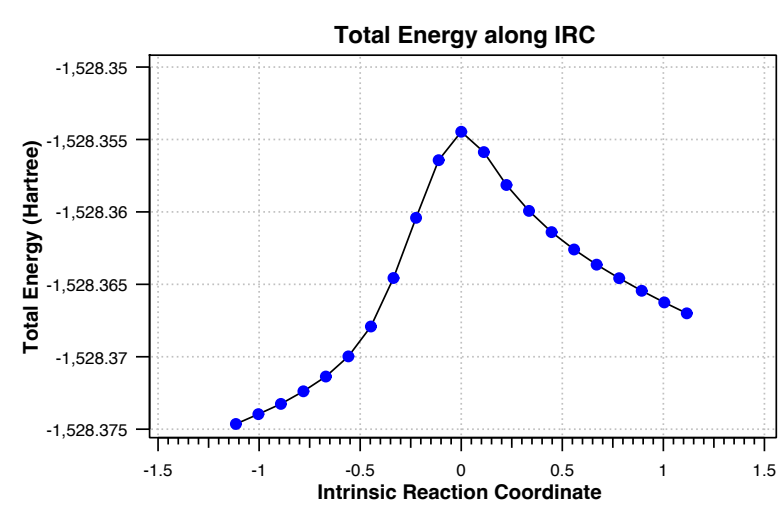

H_2Pld_{aq} – HAT@C5

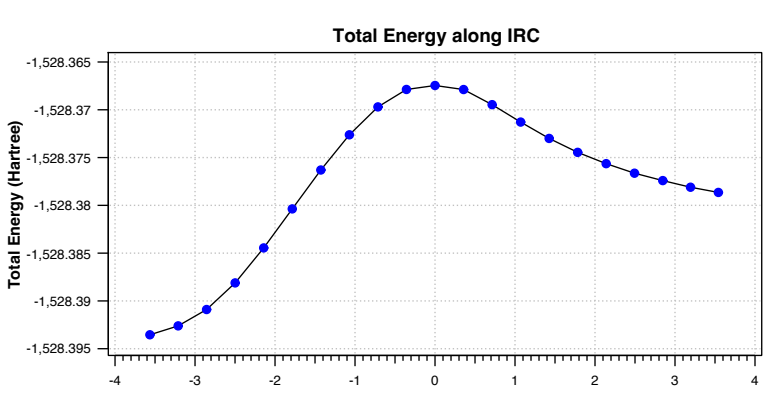

H_2Pld_{aq} – RAF@C1’

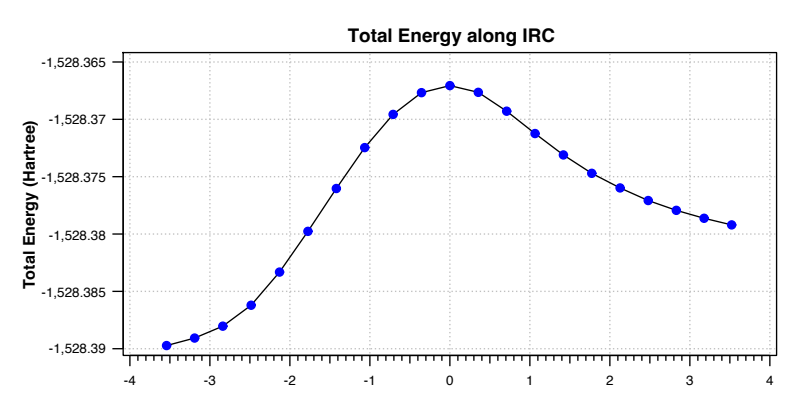

H_2Pld_{aq} – RAF@C2’

Figure S1. Intrinsic Reaction Coordinate plots for the pathways contributing most to k_{app}.

Table S1. XYZ coordinates and energies of the studied molecules.

==> AQ <==
50
XYZ-COORDINATES + ENERGIES

O	1.830294	-1.271478	0.063568
O	5.742817	-1.692247	-1.019637
O	5.084426	-1.590023	1.681366
O	2.339002	0.822160	-0.834131
O	3.275905	-1.912764	-2.738268
O	3.148754	-0.311086	3.311399
O	0.573637	5.093006	-0.075620
O	-8.155946	-1.968211	0.258702
C	4.193073	-1.009780	0.735368
C	4.388673	-1.720265	-0.587245
C	2.741425	-1.220588	1.176995
C	3.556252	-1.033821	-1.655587
C	2.212268	-0.565834	-1.091277
C	2.283573	-0.175539	2.177432
C	1.200023	1.558268	-0.592124
C	-0.069188	1.007790	-0.496462
C	1.419787	2.925997	-0.444805
C	-1.161063	1.855792	-0.258125
C	0.332020	3.749207	-0.209732
C	-0.954615	3.227878	-0.117443
C	-2.539176	1.361350	-0.135719
C	-2.924707	0.080839	-0.211802
C	-4.298059	-0.417827	-0.085108
C	-5.406398	0.408425	0.150214
C	-4.521031	-1.793143	-0.202489
C	-6.680764	-0.118766	0.262195
C	-5.794297	-2.335181	-0.092560

C	-6.874388	-1.493274	0.140277
H	4.052727	-2.755987	-0.481687
H	4.416948	0.055138	0.623962
H	2.685584	-2.205323	1.641908
H	4.100574	-0.158596	-2.012523
H	1.427539	-0.743116	-1.822961
H	1.246033	-0.362546	2.457615
H	2.374901	0.825180	1.755285
H	6.268571	-2.191256	-0.385392
H	4.878378	-1.190922	2.537180
H	4.115891	-2.209274	-3.105629
H	3.017257	0.444699	3.890982
H	-0.216174	-0.055659	-0.587704
H	2.418695	3.331229	-0.523384
H	-1.791449	3.889572	0.069003
H	-3.277526	2.136442	0.034708
H	-2.185262	-0.694082	-0.381227
H	-0.258361	5.560433	0.062354
H	-5.279265	1.477683	0.248972
H	-3.679937	-2.450348	-0.383862
H	-7.533324	0.521337	0.444468
H	-5.954340	-3.401986	-0.186062
H	-8.159022	-2.926647	0.152949

ENERGIES
SCF ENERGY: -1377.43886832
SUM OF ELECTRONIC AND ZERO-POINT ENERGIES: -1377.033389
SUM OF ELECTRONIC AND THERMAL ENERGIES: -1377.006817
SUM OF ELECTRONIC AND THERMAL ENTHALPIES: -1377.005873
SUM OF ELECTRONIC AND THERMAL FREE ENERGIES: -1377.092136

==> AQ/AIP <==
50
XYZ-COORDINATES + ENERGIES

O	1.817670	-1.269113	0.034531
O	5.754299	-1.623083	-0.998306
O	5.053546	-1.574499	1.695081
O	2.319205	0.840047	-0.827678
O	3.308008	-1.866620	-2.750359
O	3.079054	-0.361430	3.319283
O	0.524075	5.088944	-0.064131
O	-8.048324	-1.960431	0.238481
C	4.168688	-0.992970	0.744287
C	4.395303	-1.680889	-0.585366
C	2.714031	-1.231124	1.161553
C	3.566816	-0.994620	-1.657194
C	2.210884	-0.549150	-1.105277
C	2.229070	-0.212192	2.176113
C	1.172621	1.551456	-0.585363
C	-0.086514	0.988271	-0.487145
C	1.367131	2.925548	-0.435017
C	-1.184845	1.837876	-0.243892
C	0.278377	3.752535	-0.195278
C	-0.998193	3.221147	-0.100470
C	-2.530419	1.352558	-0.118227
C	-2.919959	0.032377	-0.219587
C	-4.243962	-0.432626	-0.092407
C	-5.364018	0.409991	0.176841
C	-4.469131	-1.831928	-0.242691
C	-6.616945	-0.114286	0.284190
C	-5.721315	-2.364048	-0.138523
C	-6.807096	-1.505242	0.126485
H	4.075881	-2.723577	-0.498826
H	4.378352	0.076749	0.652211
H	2.663464	-2.225863	1.604963
H	4.104217	-0.109408	-1.999748
H	1.437803	-0.720526	-1.850763
H	1.189772	-0.416995	2.436788
H	2.314973	0.798173	1.776219
H	6.280150	-2.123986	-0.365678
H	4.832471	-1.187502	2.552766
H	4.154904	-2.153101	-3.109824
H	2.926183	0.377047	3.915684
H	-0.224398	-0.075856	-0.577232
H	2.362891	3.339465	-0.513746
H	-1.846688	3.866243	0.087607
H	-3.276892	2.111888	0.070528
H	-2.175506	-0.727818	-0.416334
H	-0.303207	5.565115	0.075689
H	-5.230131	1.474188	0.299377
H	-3.621181	-2.472058	-0.445109
H	-7.477766	0.506119	0.488388
H	-5.901753	-3.424544	-0.253516
H	-8.086933	-2.920387	0.117157

ENERGIES
SCF ENERGY: -1377.23467309
SUM OF ELECTRONIC AND ZERO-POINT ENERGIES: -1376.827974
SUM OF ELECTRONIC AND THERMAL ENERGIES: -1376.801767
SUM OF ELECTRONIC AND THERMAL ENTHALPIES: -1376.800822
SUM OF ELECTRONIC AND THERMAL FREE ENERGIES: -1376.886122

==> AQ/BDE/C1P <==
49
XYZ-COORDINATES + ENERGIES

O	2.202811	-1.053632	0.846785
O	5.651921	-2.025672	-1.102419
O	5.785108	-0.466678	1.195287
O	2.033382	0.214323	-1.103074
O	2.883806	-3.104278	-1.554107
O	4.200305	1.519001	2.460788
O	-0.074406	4.324406	-1.857233
O	-7.979754	-1.999671	-0.685423
C	4.573406	-0.483471	0.448722
C	4.502054	-1.799117	-0.297509
C	3.367879	-0.406079	1.391088
C	3.296646	-1.784901	-1.219679
C	2.104418	-1.091196	-0.555034
C	3.037306	1.020802	1.788266
C	0.903682	0.966599	-0.868709
C	-0.179585	0.526008	-0.137007
C	0.936561	2.241282	-1.440508
C	-1.297952	1.396626	0.026085
C	-0.158082	3.085199	-1.273333
C	-1.271380	2.692831	-0.556290
C	-2.389773	0.982687	0.745592
C	-3.434944	0.594927	1.419827
C	-4.618204	-0.091984	0.858357
C	-4.721373	-0.400929	-0.501911
C	-5.670278	-0.440736	1.704169
C	-5.843704	-1.036667	-1.002793
C	-6.802468	-1.079586	1.213639
C	-6.885875	-1.373998	-0.141406
H	4.389981	-2.609845	0.428309
H	4.566876	0.348781	-0.261441
H	3.621406	-0.973760	2.286852
H	3.555454	-1.230930	-2.123040
H	1.194463	-1.643534	-0.776303
H	2.172368	1.028957	2.452946
H	2.828080	1.625839	0.906300
H	6.409989	-2.123882	-0.516463
H	5.765900	0.333874	1.736406
H	3.623298	-3.546283	-1.985317
H	4.128056	2.474786	2.534529
H	-0.206447	-0.445005	0.329870
H	1.799069	2.561812	-2.006817
H	-2.111840	3.362417	-0.434683
H	-3.470735	0.781148	2.493792
H	-0.883070	4.820441	-1.685889
H	-3.913174	-0.138100	-1.173039
H	-5.603906	-0.208597	2.759535
H	-5.927500	-1.275634	-2.054305
H	-7.618298	-1.346545	1.873555
H	-8.635360	-2.168121	0.000979

ENERGIES
SCF ENERGY: -1376.76171802
SUM OF ELECTRONIC AND ZERO-POINT ENERGIES: -1376.370246
SUM OF ELECTRONIC AND THERMAL ENERGIES: -1376.343771
SUM OF ELECTRONIC AND THERMAL ENTHALPIES: -1376.342827

SUM OF ELECTRONIC AND THERMAL FREE ENERGIES: -1376.429764

==> AQ/BDE/C2P <==

49

XYZ-COORDINATES + ENERGIES

O	1.305168	-1.199078	0.127924
O	4.966933	-2.466344	-1.157740
O	4.532405	-2.112472	1.564733
O	2.167125	0.701212	-0.914915
O	2.383652	-2.253589	-2.708971
O	3.015725	-0.394403	3.230835
O	1.373396	5.246791	-0.114894
O	-7.111230	-2.719481	0.274513
C	3.712831	-1.403023	0.642171
C	3.668004	-2.196170	-0.646740
C	2.283083	-1.290522	1.180477
C	2.918286	-1.402510	-1.702205
C	1.742438	-0.640443	-1.085919
C	2.118315	-0.131149	2.145518
C	1.219720	1.658470	-0.628462
C	-0.131013	1.388869	-0.466603
C	1.723773	2.951576	-0.506250
C	-0.999305	2.449597	-0.184816
C	0.846921	3.985528	-0.226477
C	-0.515870	3.748107	-0.066062
C	-2.446500	2.216344	0.007507
C	-3.040225	1.051807	-0.032883
C	-4.074337	0.131540	0.032465
C	-4.409971	-0.503468	1.258555
C	-4.797675	-0.243796	-1.129784
C	-5.418857	-1.439982	1.316593
C	-5.806193	-1.183017	-1.062572
C	-6.119804	-1.782495	0.158602
H	3.137741	-3.134892	-0.461526
H	4.139597	-0.413045	0.457084
H	2.060533	-2.221450	1.702774
H	3.602011	-0.677541	-2.145635
H	0.889528	-0.682310	-1.758776
H	1.086771	-0.087895	2.497593
H	2.380498	0.810063	1.662212
H	5.420073	-3.039831	-0.530529
H	4.472885	-1.642098	2.406968
H	3.115738	-2.726112	-3.119804
H	3.071148	0.390668	3.782905
H	-0.528414	0.387526	-0.535506
H	2.780618	3.139808	-0.633195
H	-1.186590	4.569350	0.154455
H	-3.039514	3.111173	0.204757
H	0.671141	5.874556	0.090007
H	-3.864504	-0.239195	2.153621
H	-4.553202	0.219678	-2.075210
H	-5.679022	-1.917820	2.251370
H	-6.360391	-1.460200	-1.950682
H	-7.523764	-2.873099	-0.583455

ENERGIES

SCF ENERGY: -1376.76256846

SUM OF ELECTRONIC AND ZERO-POINT ENERGIES: -1376.370972

SUM OF ELECTRONIC AND THERMAL ENERGIES: -1376.344520

SUM OF ELECTRONIC AND THERMAL ENTHALPIES: -1376.343575

SUM OF ELECTRONIC AND THERMAL FREE ENERGIES: -1376.429943

==> AQ/BDE/C4PP <==

49

XYZ-COORDINATES + ENERGIES

O	1.805983	-1.242736	0.146904
O	5.689722	-1.746043	-0.988962
O	5.092179	-1.455640	1.709881
O	2.256070	0.784869	-0.914417
O	3.218861	-2.031483	-2.660806
O	3.196408	-0.036899	3.281413
O	0.460716	5.049333	-0.209452
O	-8.083887	-2.052837	0.338186
C	4.182368	-0.935867	0.746892
C	4.344624	-1.733645	-0.529456
C	2.740158	-1.104886	1.233815
C	3.498783	-1.103407	-1.620254
C	2.155031	-0.622427	-1.064901
C	2.309366	0.019248	2.157657
C	1.112062	1.508209	-0.669319
C	-0.148123	0.946768	-0.540450
C	1.314322	2.882427	-0.553377
C	-1.242967	1.793123	-0.299828
C	0.225157	3.704181	-0.313893
C	-1.052612	3.172658	-0.187388
C	-2.602483	1.295203	-0.147665
C	-2.985853	-0.008450	-0.224952
C	-4.320250	-0.486011	-0.070724
C	-5.439522	0.360847	0.201610
C	-4.548605	-1.889141	-0.194192
C	-6.687425	-0.153084	0.338476
C	-5.790707	-2.423046	-0.061516
C	-6.933389	-1.577410	0.213602
H	4.001005	-2.757049	-0.352866
H	4.408585	0.117796	0.557507
H	2.690592	-2.048886	1.777092
H	4.033275	-0.240335	-2.019388
H	1.362906	-0.870750	-1.767263
H	1.275979	-0.134462	2.471425
H	2.402050	0.983277	1.657628
H	6.223682	-2.209427	-0.334817
H	4.902267	-1.003896	2.542909
H	4.059229	-2.329632	-3.025963
H	3.101244	0.773378	3.790207
H	-0.287400	-0.119227	-0.606827
H	2.308047	3.295767	-0.654549
H	-1.894950	3.825907	0.002395
H	-3.343276	2.060845	0.042495
H	-2.244386	-0.773340	-0.419588
H	-0.369935	5.515198	-0.057888
H	-5.294513	1.426989	0.300974
H	-3.699040	-2.527661	-0.399870
H	-7.538569	0.481621	0.545372
H	-5.964804	-3.486343	-0.155797

ENERGIES

SCF ENERGY: -1376.79803807

SUM OF ELECTRONIC AND ZERO-POINT ENERGIES: -1376.405258

SUM OF ELECTRONIC AND THERMAL ENERGIES: -1376.378977

SUM OF ELECTRONIC AND THERMAL ENTHALPIES: -1376.378033

SUM OF ELECTRONIC AND THERMAL FREE ENERGIES: -1376.464434

==> AQ/BDE/C4PP/TS <==

53

XYZ-COORDINATES + ENERGIES

O	2.347615	-1.298000	-0.003436
O	6.272281	-1.938291	-0.950451
O	5.500231	-1.929854	1.728524
O	3.034513	0.793837	-0.782584
O	3.871469	-1.903743	-2.777775
O	3.603275	-0.578888	3.333152
O	1.570784	5.091111	0.377188
O	-7.690762	-1.136793	-0.742467
C	4.694478	-1.241357	0.776998
C	4.900601	-1.897695	-0.573695
C	3.211535	-1.368558	1.147155
C	4.163574	-1.101182	-1.638857
C	2.826709	-0.570854	-1.108193
C	2.784838	-0.340090	2.181248
C	1.941753	1.590184	-0.520127
C	0.626896	1.141673	-0.549764
C	2.257614	2.913744	-0.207898
C	-0.407127	2.054055	-0.274660
C	1.225671	3.798737	0.070548
C	-0.104387	3.383198	0.038438
C	-1.823343	1.671880	-0.289788
C	-2.314701	0.460460	-0.624186
C	-3.724282	0.091602	-0.628256
C	-4.762138	0.970296	-0.242074
C	-4.066794	-1.216304	-1.035087

C	-6.077905	0.560362	-0.266228
C	-5.380423	-1.640449	-1.057852
C	-6.406776	-0.752758	-0.675578
H	4.494540	-2.914000	-0.538771
H	4.999428	-0.190391	0.732023
H	3.063558	-2.369599	1.556405
H	4.782283	-0.249149	-1.928995
H	2.064807	-0.664682	-1.879920
H	1.728099	-0.472263	2.423452
H	2.950492	0.672126	1.808930
H	6.734556	-2.533810	-0.341721
H	5.297252	-1.542439	2.597245
H	4.708706	-2.242475	-3.130109
H	3.477854	0.148033	3.959194
H	0.398503	0.109160	-0.762845
H	3.289933	3.238174	-0.183351
H	-0.897523	4.089480	0.257359
H	-2.502654	2.467827	-0.004496
H	-1.639588	-0.334326	-0.923778
H	0.771958	5.605982	0.569523
H	-4.533455	1.977960	0.078573
H	-3.276377	-1.896008	-1.330766
H	-6.878813	1.228207	0.026739
H	-5.644710	-2.644239	-1.366004
H	-8.019046	-1.436131	0.244534
O	-8.043261	-1.776052	1.555047
O	-6.730803	-1.621495	1.864571
H	-6.647291	-0.703771	2.189751

ENERGIES
SCF ENERGY: -1528.35847035
SUM OF ELECTRONIC AND ZERO-POINT ENERGIES: -1527.941029
SUM OF ELECTRONIC AND THERMAL ENERGIES: -1527.912647
SUM OF ELECTRONIC AND THERMAL ENTHALPIES: -1527.911703
SUM OF ELECTRONIC AND THERMAL FREE ENERGIES: -1528.001749

==> AQ/BDE/C5 <==
49
XYZ-COORDINATES + ENERGIES

O	1.771720	-1.169636	0.152985
O	5.626704	-1.922319	-0.949791
O	5.034933	-1.532423	1.738604
O	2.328230	0.794893	-0.972519
O	3.160033	-2.110215	-2.630710
O	3.212842	0.035579	3.264424
O	0.591704	5.063913	-0.223682
O	-8.094580	-1.971282	0.337386
C	4.160813	-0.985448	0.757980
C	4.283139	-1.823409	-0.497102
C	2.709505	-1.058839	1.241583
C	3.479753	-1.175009	-1.608885
C	2.158320	-0.614215	-1.076513
C	2.338633	0.111855	2.132820
C	1.236098	1.582858	-0.713334
C	-0.064837	1.068082	-0.564195
C	1.477623	2.925861	-0.598018
C	-1.163638	1.931878	-0.298348
C	0.389154	3.830769	-0.329632
C	-0.938090	3.280401	-0.184111
C	-2.529036	1.422817	-0.141355
C	-2.899806	0.136963	-0.211878
C	-4.263539	-0.376743	-0.060312
C	-5.376899	0.437162	0.193563
C	-4.470731	-1.754707	-0.176415
C	-6.642532	-0.105389	0.324193
C	-5.735169	-2.311838	-0.048011
C	-6.821366	-1.482281	0.202281
H	3.881750	-2.821014	-0.296826
H	4.447653	0.048593	0.543648
H	2.602328	-1.984444	1.807366
H	4.061326	-0.349597	-2.021647
H	1.359593	-0.846786	-1.776491
H	1.295567	0.025803	2.441030
H	2.490035	1.055658	1.608906
H	6.129279	-2.409355	-0.288161
H	4.877232	-1.043171	2.556842
H	3.986475	-2.463539	-2.978048
H	3.142370	0.854414	3.763568
H	-0.233512	0.007659	-0.644463
H	2.476835	3.323376	-0.706688
H	-1.747718	3.968496	0.018901
H	-3.269006	2.191654	0.046308
H	-2.158342	-0.631551	-0.398251
H	-5.260616	1.507774	0.290873
H	-3.624463	-2.400925	-0.372303
H	-7.500178	0.523716	0.520015
H	-5.884078	-3.380184	-0.141399
H	-8.089594	-2.929264	0.227374

ENERGIES
SCF ENERGY: -1376.78649039
SUM OF ELECTRONIC AND ZERO-POINT ENERGIES: -1376.393750
SUM OF ELECTRONIC AND THERMAL ENERGIES: -1376.367629
SUM OF ELECTRONIC AND THERMAL ENTHALPIES: -1376.366685
SUM OF ELECTRONIC AND THERMAL FREE ENERGIES: -1376.452360

==> AQ/BDE/C5/TS <==
53
XYZ-COORDINATES + ENERGIES

O	1.914036	-0.971927	1.534929
O	5.775543	-1.731284	0.450059
O	5.249917	-0.713159	2.987089
O	2.309134	0.682079	-0.058840
O	3.243971	-2.513709	-0.974755
O	3.354806	1.049065	4.192029
O	0.529394	5.013358	0.270512
O	-8.009491	-2.014136	2.055509
C	4.302712	-0.493535	1.947969
C	4.445179	-1.612037	0.937081
C	2.879388	-0.536432	2.512260
C	3.550531	-1.325387	-0.256232
C	2.219640	-0.714639	0.188819
C	2.456614	0.795035	3.105702
C	1.188947	1.454716	0.137469
C	-0.074462	0.944782	0.385600
C	1.406085	2.826558	0.087685
C	-1.169629	1.820324	0.587415
C	0.322870	3.700427	0.294062
C	-0.962684	3.187844	0.546364
C	-2.523081	1.326362	0.852852
C	-2.877328	0.039133	0.976681
C	-4.226954	-0.460877	1.247637
C	-5.361360	0.361280	1.311795
C	-4.398443	-1.833238	1.455322
C	-6.611169	-0.166930	1.580302
C	-5.646267	-2.375653	1.727519
C	-6.752342	-1.537560	1.790299
H	4.132661	-2.550964	1.403161
H	4.501048	0.468921	1.466851
H	2.861267	-1.297007	3.293233
H	4.055743	-0.610338	-0.906896
H	1.411253	-1.151764	-0.391919
H	1.426093	0.732890	3.459930
H	2.543493	1.586098	2.363035
H	6.339029	-2.006199	1.181142
H	5.079335	-0.044778	3.663973
H	4.070584	-2.898151	-1.285855
H	3.266964	1.971166	4.451433
H	-0.229191	-0.120399	0.438883
H	2.394516	3.223700	-0.095070
H	-1.778028	3.879947	0.708066
H	-3.269527	2.104749	0.959483
H	-2.127554	-0.738723	0.884669
H	-5.273848	1.426285	1.146241
H	-3.536136	-2.486016	1.406279
H	-7.485449	0.468001	1.627016
H	-5.766434	-3.439356	1.890580
H	-7.973418	-2.969716	2.178590
H	0.900782	5.320335	1.289747
O	1.286202	5.333207	2.489743

O	1.623335	4.033145	2.699630
H	0.825999	3.604946	3.055619

ENERGIES
SCF ENERGY: -1528.35446658
SUM OF ELECTRONIC AND ZERO-POINT ENERGIES: -1527.937936
SUM OF ELECTRONIC AND THERMAL ENERGIES: -1527.908497
SUM OF ELECTRONIC AND THERMAL ENTHALPIES: -1527.907553
SUM OF ELECTRONIC AND THERMAL FREE ENERGIES: -1528.001244

==> AQ/PA/C4PP <==
49
XYZ-COORDINATES + ENERGIES

O	1.779245	-1.259884	0.013954
O	5.690842	-1.670124	-1.066322
O	5.019166	-1.676180	1.634219
O	2.296354	0.868629	-0.792965
O	3.241643	-1.778512	-2.809204
O	3.091665	-0.421066	3.298358
O	0.511628	5.114815	0.061125
O	-8.111558	-2.149539	0.180654
C	4.139805	-1.045070	0.709811
C	4.334162	-1.699128	-0.641741
C	2.683002	-1.257447	1.134234
C	3.516988	-0.954976	-1.682313
C	2.170256	-0.506878	-1.107817
C	2.228715	-0.244139	2.168488
C	1.153431	1.597130	-0.539649
C	-0.115675	1.042172	-0.477332
C	1.370453	2.960153	-0.350937
C	-1.216192	1.878588	-0.229163
C	0.275357	3.773183	-0.111336
C	-1.011255	3.248472	-0.052814
C	-2.591028	1.374860	-0.138171
C	-2.954763	0.081013	-0.148581
C	-4.309515	-0.451608	-0.063317
C	-5.468275	0.344746	-0.028835
C	-4.491658	-1.842353	-0.014052
C	-6.727217	-0.214739	0.053693
C	-5.747061	-2.417073	0.070413
C	-6.925445	-1.625113	0.107051
H	3.984478	-2.733978	-0.584038
H	4.376550	0.021049	0.645649
H	2.614844	-2.257772	1.562681
H	4.070539	-0.067220	-1.990966
H	1.390536	-0.652905	-1.851553
H	1.189924	-0.435349	2.441088
H	2.325202	0.769427	1.779338
H	6.203020	-2.221827	-0.465643
H	4.818811	-1.305859	2.504046
H	4.082984	-2.073369	-3.174911
H	2.960944	0.314801	3.903094
H	-0.259858	-0.017454	-0.608830
H	2.368901	3.370006	-0.406796
H	-1.851187	3.905081	0.138276
H	-3.347159	2.145798	-0.040605
H	-2.184886	-0.681149	-0.220158
H	-0.324058	5.576500	0.195592
H	-5.382528	1.423558	-0.071608
H	-3.616403	-2.482128	-0.041685
H	-7.604006	0.421581	0.075370
H	-5.853035	-3.494664	0.107298

ENERGIES
SCF ENERGY: -1376.96832020
SUM OF ELECTRONIC AND ZERO-POINT ENERGIES: -1376.576113
SUM OF ELECTRONIC AND THERMAL ENERGIES: -1376.549916
SUM OF ELECTRONIC AND THERMAL ENTHALPIES: -1376.548972
SUM OF ELECTRONIC AND THERMAL FREE ENERGIES: -1376.634527

==> AQ/PA/C4PP/AIP <==
49
XYZ-COORDINATES + ENERGIES

O	1.804806	-1.261822	0.106709
O	5.713381	-1.660377	-1.002444
O	5.080743	-1.471824	1.698485
O	2.265853	0.802399	-0.877462
O	3.236726	-1.981990	-2.688150
O	3.142996	-0.163495	3.302177
O	0.458279	5.058662	-0.155924
O	-8.080389	-2.053696	0.300565
C	4.170724	-0.939956	0.742069
C	4.364220	-1.695162	-0.555694
C	2.727441	-1.155590	1.207277
C	3.510561	-1.061218	-1.639324
C	2.163286	-0.598094	-1.079146
C	2.265493	-0.079918	2.172745
C	1.119218	1.522074	-0.635532
C	-0.140604	0.956991	-0.519102
C	1.318338	2.895727	-0.508126
C	-1.238701	1.799859	-0.280386
C	0.226024	3.713811	-0.270721
C	-1.051587	3.179000	-0.157502
C	-2.598498	1.299442	-0.139857
C	-2.979848	-0.004496	-0.222063
C	-4.314769	-0.483392	-0.078089
C	-5.436455	0.361965	0.188784
C	-4.541423	-1.886378	-0.206531
C	-6.684980	-0.153092	0.315422
C	-5.784062	-2.421400	-0.084010
C	-6.929270	-1.577229	0.185189
H	4.046230	-2.731645	-0.409431
H	4.377262	0.123360	0.589300
H	2.691024	-2.122770	1.709366
H	4.035694	-0.189274	-2.031316
H	1.375700	-0.818405	-1.795903
H	1.232844	-0.268301	2.469543
H	2.341540	0.905576	1.713197
H	6.254773	-2.119803	-0.351580
H	4.875209	-1.048023	2.542491
H	4.078851	-2.275112	-3.053324
H	3.012338	0.613996	3.852563
H	-0.276486	-0.108967	-0.593684
H	2.311863	3.311571	-0.600492
H	-1.896626	3.829343	0.030225
H	-3.341726	2.063726	0.046435
H	-2.236167	-0.768335	-0.412480
H	-0.374468	5.522402	-0.009420
H	-5.292939	1.427954	0.292403
H	-3.690046	-2.523857	-0.407986
H	-7.537881	0.480627	0.518226
H	-5.956778	-3.484598	-0.182196

ENERGIES
SCF ENERGY: -1376.79804368
SUM OF ELECTRONIC AND ZERO-POINT ENERGIES: -1376.405391
SUM OF ELECTRONIC AND THERMAL ENERGIES: -1376.379029
SUM OF ELECTRONIC AND THERMAL ENTHALPIES: -1376.378084
SUM OF ELECTRONIC AND THERMAL FREE ENERGIES: -1376.464933

==> AQ/PA/C4PP/BDE/C1P <==
48
XYZ-COORDINATES + ENERGIES

O	2.155028	-1.037081	0.862673
O	5.573383	-2.046068	-1.119731
O	5.744857	-0.476575	1.167304
O	1.970555	0.223022	-1.091428
O	2.791336	-3.102709	-1.538456
O	4.188824	1.531677	2.436892
O	-0.156653	4.313781	-1.904495
O	-7.931212	-2.130257	-0.674562
C	4.524626	-0.487060	0.434504
C	4.434045	-1.805948	-0.303935
C	3.330527	-0.394817	1.389846
C	3.218798	-1.785638	-1.213045
C	2.039404	-1.079827	-0.537929
C	3.014425	1.036691	1.781952
C	0.843306	0.980293	-0.855937
C	-0.228293	0.555047	-0.098503
C	0.866991	2.242354	-1.456000

C	-1.348893	1.424778	0.061838
C	-0.227755	3.086785	-1.291627
C	-1.329973	2.707722	-0.550933
C	-2.427621	1.025976	0.811382
C	-3.487837	0.613228	1.451146
C	-4.638638	-0.103159	0.872531
C	-4.714047	-0.434293	-0.487523
C	-5.708102	-0.477260	1.692496
C	-5.804800	-1.106499	-1.003570
C	-6.808197	-1.151649	1.187387
C	-6.903914	-1.496318	-0.186210
H	4.322920	-2.612032	0.427186
H	4.517208	0.341529	-0.279932
H	3.590082	-0.958873	2.286184
H	3.472277	-1.237058	-2.121168
H	1.122915	-1.626837	-0.745268
H	2.157868	1.054568	2.457189
H	2.798017	1.637365	0.898719
H	6.337974	-2.140060	-0.541555
H	5.738182	0.326568	1.704812
H	3.519004	-3.550036	-1.983968
H	4.120510	2.487770	2.510726
H	-0.245557	-0.405327	0.390440
H	1.720514	2.551558	-2.041868
H	-2.169921	3.378892	-0.433596
H	-3.554391	0.805031	2.523361
H	-0.964701	4.810312	-1.731526
H	-3.898223	-0.156634	-1.146088
H	-5.670680	-0.231878	2.748132
H	-5.843132	-1.353066	-2.058015
H	-7.624110	-1.430559	1.843449

ENERGIES
SCF ENERGY: -1376.29039671
SUM OF ELECTRONIC AND ZERO-POINT ENERGIES: -1375.912146
SUM OF ELECTRONIC AND THERMAL ENERGIES: -1375.885969
SUM OF ELECTRONIC AND THERMAL ENTHALPIES: -1375.885024
SUM OF ELECTRONIC AND THERMAL FREE ENERGIES: -1375.972365

==> AQ/PA/C4PP/BDE/C2P <==
48
XYZ-COORDINATES + ENERGIES

O	1.380683	-1.213950	0.139564
O	5.090971	-2.312045	-1.166349
O	4.657027	-1.971395	1.556713
O	2.152981	0.717167	-0.917632
O	2.478021	-2.242315	-2.692736
O	3.073478	-0.321310	3.228133
O	1.166717	5.228883	-0.134189
O	-7.348698	-2.693782	0.204202
C	3.798637	-1.304023	0.637749
C	3.783502	-2.104205	-0.647485
C	2.368802	-1.258307	1.185385
C	2.986221	-1.356573	-1.702031
C	1.784286	-0.641643	-1.079799
C	2.154883	-0.106620	2.149836
C	1.166726	1.635372	-0.629439
C	-0.169335	1.308288	-0.456462
C	1.616060	2.949492	-0.517000
C	-1.084643	2.329186	-0.172609
C	0.695307	3.943997	-0.234944
C	-0.654331	3.647949	-0.061703
C	-2.518993	2.030031	0.026061
C	-3.060497	0.834778	-0.041939
C	-4.170741	-0.000105	0.032282
C	-4.946954	-0.309437	-1.119516
C	-4.528622	-0.652624	1.245226
C	-6.002430	-1.187932	-1.057415
C	-5.584811	-1.530385	1.302114
C	-6.368676	-1.849053	0.152999
H	3.302306	-3.067195	-0.453117
H	4.177063	-0.295745	0.446899
H	2.194219	-2.198169	1.709976
H	3.630876	-0.606438	-2.161792
H	0.928722	-0.723119	-1.745542
H	1.125556	-0.114570	2.510739
H	2.365012	0.845394	1.662151
H	5.578360	-2.852721	-0.535584
H	4.580270	-1.503012	2.398577
H	3.224138	-2.694779	-3.100961
H	3.088263	0.464459	3.781748
H	-0.523321	0.290395	-0.517357
H	2.662534	3.183648	-0.652792
H	-1.357960	4.441039	0.160191
H	-3.143062	2.898336	0.248741
H	0.438128	5.826210	0.069356
H	-4.692935	0.164430	-2.059282
H	-3.949992	-0.444847	2.136460
H	-6.582560	-1.402329	-1.947028
H	-5.841401	-2.010134	2.239085

ENERGIES
SCF ENERGY: -1376.29458920
SUM OF ELECTRONIC AND ZERO-POINT ENERGIES: -1375.916065
SUM OF ELECTRONIC AND THERMAL ENERGIES: -1375.889885
SUM OF ELECTRONIC AND THERMAL ENTHALPIES: -1375.888941
SUM OF ELECTRONIC AND THERMAL FREE ENERGIES: -1375.975280

==> AQ/PA/C4PP/BDE/C5 <==
48
XYZ-COORDINATES + ENERGIES

O	1.814261	-1.237331	0.157285
O	5.703540	-1.699691	-0.972008
O	5.101940	-1.377933	1.722800
O	2.255024	0.781105	-0.928564
O	3.220361	-2.073539	-2.623385
O	3.189320	0.045721	3.268363
O	0.425383	5.058026	-0.214277
O	-8.096649	-2.007575	0.368020
C	4.184984	-0.884890	0.751623
C	4.358386	-1.701636	-0.511086
C	2.744773	-1.065582	1.241059
C	3.498956	-1.110513	-1.613694
C	2.155446	-0.622884	-1.063044
C	2.298427	0.068274	2.145563
C	1.103811	1.513435	-0.688219
C	-0.153557	0.943772	-0.567958
C	1.314202	2.883939	-0.571149
C	-1.239550	1.810499	-0.328271
C	0.243968	3.775470	-0.328639
C	-1.043076	3.185729	-0.214050
C	-2.600230	1.320143	-0.175958
C	-2.992231	0.015229	-0.235485
C	-4.325938	-0.455035	-0.075264
C	-5.445517	0.398029	0.176831
C	-4.559406	-1.860196	-0.167820
C	-6.694575	-0.111122	0.324500
C	-5.802663	-2.388663	-0.023063
C	-6.943750	-1.536452	0.232539
H	4.033589	-2.727445	-0.313648
H	4.398448	0.168147	0.546351
H	2.709847	-2.000015	1.802041
H	4.023062	-0.256103	-2.043784
H	1.361813	-0.885193	-1.758778
H	1.268547	-0.096288	2.465347
H	2.373624	1.024524	1.628458
H	6.247621	-2.130967	-0.304355
H	4.909025	-0.911372	2.546889
H	4.061341	-2.381315	-2.978848
H	3.087909	0.866039	3.759474
H	-0.298084	-0.121223	-0.632110
H	2.320164	3.271432	-0.671462
H	-1.891726	3.832292	-0.025486
H	-3.340401	2.089534	0.003456
H	-2.252048	-0.754577	-0.415751
H	-5.299427	1.466088	0.251967
H	-3.711559	-2.505728	-0.358818
H	-7.544307	0.530398	0.516136
H	-5.977655	-3.453778	-0.093435

ENERGIES
SCF ENERGY: -1376.32886680
SUM OF ELECTRONIC AND ZERO-POINT ENERGIES: -1375.948910
SUM OF ELECTRONIC AND THERMAL ENERGIES: -1375.923050
SUM OF ELECTRONIC AND THERMAL ENTHALPIES: -1375.922106
SUM OF ELECTRONIC AND THERMAL FREE ENERGIES: -1376.007870

==> AQ/PA/C4PP/BDE/C5/TS <==
52
XYZ-COORDINATES + ENERGIES

O	2.092682	-0.336631	2.025016
O	5.415521	-2.301060	0.851296
O	5.073065	-1.233552	3.456568
O	1.835714	0.615289	-0.109005
O	3.166698	-2.053417	-0.876752
O	4.363286	2.360696	3.053582
O	0.026488	4.824504	1.025210
O	-8.450281	-2.373578	1.275265
C	4.541926	-0.619322	2.288844
C	4.201341	-1.718456	1.302717
C	3.286155	0.223081	2.601551
C	3.438285	-1.099627	0.140575
C	2.098702	-0.565267	0.637388
C	3.414859	1.690858	2.217186
C	0.696811	1.339717	0.232634
C	-0.545765	0.741598	0.379477
C	0.885652	2.706378	0.391423
C	-1.633688	1.572074	0.717166
C	-0.174723	3.555884	0.781164
C	-1.446051	2.944870	0.915550
C	-2.985331	1.053646	0.882413
C	-3.369127	-0.246020	0.727409
C	-4.697643	-0.745171	0.882981
C	-5.821150	0.073845	1.222897
C	-4.919036	-2.142766	0.685099
C	-7.064744	-0.460435	1.354228
C	-6.156103	-2.696302	0.812478
C	-7.305274	-1.880015	1.155955
H	3.576323	-2.468288	1.797602
H	5.305788	0.016813	1.826004
H	3.115909	0.172655	3.676509
H	4.029227	-0.272809	-0.264498
H	1.321065	-1.307875	0.464962
H	2.454851	2.172125	2.387711
H	3.688899	1.807215	1.168075
H	5.182581	-3.101329	0.358827
H	5.402313	-0.524980	4.028357
H	3.967221	-2.144599	-1.412425
H	5.246534	2.228370	2.680023
H	-0.665856	-0.322274	0.235698
H	1.875029	3.124927	0.259776
H	-2.289247	3.564692	1.198881
H	-3.726211	1.798190	1.148654
H	-2.626930	-0.989885	0.461094
H	-5.682486	1.135479	1.377238
H	-4.067794	-2.761885	0.427795
H	-7.918018	0.154591	1.610250
H	-6.322535	-3.755297	0.662428
H	1.313977	4.856093	1.970205
O	2.019461	4.856122	2.699968
O	3.242331	4.786759	1.951854
H	3.696384	4.044251	2.398653

ENERGIES
SCF ENERGY: -1527.42967534
SUM OF ELECTRONIC AND ZERO-POINT ENERGIES: -1527.016831
SUM OF ELECTRONIC AND THERMAL ENERGIES: -1526.988727
SUM OF ELECTRONIC AND THERMAL ENTHALPIES: -1526.987783
SUM OF ELECTRONIC AND THERMAL FREE ENERGIES: -1527.078222

==> AQ/PA/C4PP/PA/C5 <==
48
XYZ-COORDINATES + ENERGIES

O	1.801567	-1.263180	0.063884
O	5.709313	-1.637213	-1.042619
O	5.067554	-1.520748	1.663968
O	2.288194	0.832148	-0.844991
O	3.225334	-1.917672	-2.734608
O	3.130558	-0.227364	3.285589
O	0.488783	5.100196	0.003500
O	-8.157439	-2.074996	0.247391
C	4.163747	-0.958589	0.717972
C	4.358509	-1.679178	-0.599301
C	2.716732	-1.180909	1.170697
C	3.507421	-1.017553	-1.669057
C	2.165537	-0.551734	-1.097143
C	2.251648	-0.126253	2.157733
C	1.141761	1.575058	-0.594774
C	-0.126739	1.016722	-0.527056
C	1.371730	2.934037	-0.416274
C	-1.212722	1.878371	-0.276621
C	0.299770	3.820706	-0.166626
C	-0.997423	3.242616	-0.108818
C	-2.594276	1.384149	-0.171469
C	-2.973076	0.095562	-0.164909
C	-4.335927	-0.421837	-0.061101
C	-5.485825	0.386317	-0.035107
C	-4.533614	-1.808394	0.018044
C	-6.751179	-0.158023	0.068645
C	-5.795482	-2.367810	0.124052
C	-6.964505	-1.563530	0.153220
H	4.038573	-2.718429	-0.479917
H	4.376414	0.107182	0.595909
H	2.677609	-2.158568	1.652288
H	4.039511	-0.143530	-2.046721
H	1.375698	-0.740995	-1.820203
H	1.220426	-0.325802	2.452225
H	2.320912	0.868093	1.717130
H	6.245650	-2.129876	-0.412451
H	4.863346	-1.113746	2.516409
H	4.064774	-2.213976	-3.103223
H	2.992242	0.536738	3.852539
H	-0.281242	-0.042231	-0.649644
H	2.382993	3.314988	-0.480919
H	-1.840049	3.896747	0.084111
H	-3.344289	2.162105	-0.076023
H	-2.212428	-0.676107	-0.234237
H	-5.389134	1.463074	-0.101578
H	-3.666057	-2.458956	-0.002562
H	-7.620422	0.488917	0.083194
H	-5.911959	-3.443378	0.184108

ENERGIES
SCF ENERGY: -1376.49576733
SUM OF ELECTRONIC AND ZERO-POINT ENERGIES: -1376.116789
SUM OF ELECTRONIC AND THERMAL ENERGIES: -1376.090787
SUM OF ELECTRONIC AND THERMAL ENTHALPIES: -1376.089843
SUM OF ELECTRONIC AND THERMAL FREE ENERGIES: -1376.175663

==> AQ/PA/C4PP/RAF/C1 <==
52
XYZ-COORDINATES + ENERGIES

O	2.302697	-0.856457	1.141695
O	5.202362	-2.812215	-0.959579
O	5.914670	-0.670391	0.666871
O	1.846705	-0.220471	-1.056243
O	2.328518	-3.607042	-0.531011
O	4.771319	1.787024	1.501206
O	-0.020226	3.683266	-2.838689
O	-7.965714	-2.400254	0.172302
C	4.577618	-0.751882	0.184910
C	4.262804	-2.209010	-0.079432
C	3.599401	-0.239761	1.246622
C	2.895441	-2.317980	-0.730358
C	1.920956	-1.297714	-0.136930
C	3.463322	1.271626	1.224823
C	0.853216	0.716577	-0.882384
C	-0.061719	0.697084	0.114908
C	0.872612	1.730727	-1.874739
C	-1.101736	1.762503	0.222140

C	-0.091579	2.753535	-1.827453
C	-1.033862	2.802272	-0.848369
C	-2.511189	1.234758	0.299490
C	-2.850078	-0.047947	0.117133
C	-4.197141	-0.614765	0.137735
C	-5.361578	0.136035	0.372826
C	-4.358895	-1.990025	-0.083202
C	-6.610158	-0.452842	0.384557
C	-5.603892	-2.594216	-0.074943
C	-6.789015	-1.848733	0.161144
H	4.248810	-2.744056	0.874622
H	4.485344	-0.176681	-0.741005
H	3.987411	-0.546900	2.218267
H	2.999352	-2.112527	-1.796610
H	0.941126	-1.754370	-0.016366
H	2.751661	1.590467	1.987708
H	3.128015	1.612480	0.245384
H	6.061164	-2.814680	-0.523398
H	6.059603	0.249905	0.924966
H	2.932384	-4.257741	-0.905453
H	4.784395	2.723762	1.285488
H	-0.059589	-0.051608	0.891453
H	1.615404	1.706914	-2.657768
H	-1.781254	3.585398	-0.842529
H	-3.264015	1.995623	0.466983
H	-2.062863	-0.770517	-0.076185
H	-0.717604	4.339282	-2.724097
H	-5.288782	1.202169	0.549833
H	-3.477380	-2.594479	-0.266927
H	-7.492567	0.148564	0.568310
H	-5.694984	-3.659437	-0.250442
O	-0.743265	2.382165	1.529427
O	-1.660274	3.423839	1.839345
H	-1.217141	4.211275	1.494304

ENERGIES
SCF ENERGY: -1527.88740203
SUM OF ELECTRONIC AND ZERO-POINT ENERGIES: -1527.478617
SUM OF ELECTRONIC AND THERMAL ENERGIES: -1527.449655
SUM OF ELECTRONIC AND THERMAL ENTHALPIES: -1527.448710
SUM OF ELECTRONIC AND THERMAL FREE ENERGIES: -1527.540533

==> AQ/PA/C4PP/RAF/C1P <==
52
XYZ-COORDINATES + ENERGIES

O	2.076726	-1.061869	-0.742470
O	6.155931	-0.987762	-0.869098
O	4.886389	-2.446315	1.126717
O	2.561308	1.131946	-0.111212
O	4.185224	-0.021235	-2.939811
O	2.515816	-2.245833	2.666491
O	0.187844	3.984708	2.744788
O	-7.780449	-2.411034	-0.008747
C	4.182698	-1.374087	0.509181
C	4.747972	-1.186219	-0.882885
C	2.696881	-1.721687	0.376757
C	4.120411	0.040666	-1.520067
C	2.641845	0.161533	-1.142289
C	1.920844	-1.431975	1.648370
C	1.319809	1.600205	0.255598
C	0.128548	1.187141	-0.328616
C	1.333588	2.533808	1.287525
C	-1.066758	1.737800	0.136055
C	0.132975	3.065404	1.728571
C	-1.076037	2.680373	1.152933
C	-2.374168	1.261062	-0.448478
C	-2.682868	-0.136757	-0.055173
C	-3.978327	-0.678565	-0.057469
C	-5.138459	0.044999	-0.459031
C	-4.189418	-2.026655	0.356756
C	-6.388553	-0.526525	-0.440662
C	-5.436437	-2.598175	0.373626
C	-6.602745	-1.874578	-0.023289
H	4.501815	-2.067140	-1.483033
H	4.319095	-0.459573	1.093705
H	2.632821	-2.787566	0.156133
H	4.640996	0.927790	-1.157067
H	2.071901	0.484984	-2.009758
H	0.870401	-1.692138	1.508267
H	2.004670	-0.379006	1.916789
H	6.564885	-1.786231	-0.518300
H	4.449033	-2.612856	1.972525
H	5.111357	-0.101196	-3.192138
H	2.200112	-1.945912	3.523674
H	0.100325	0.444119	-1.110990
H	2.269818	2.839013	1.733613
H	-2.006876	3.107907	1.504878
H	-3.170418	1.952672	-0.171071
H	-1.851798	-0.772003	0.222007
H	-0.703775	4.260820	2.986928
H	-5.036729	1.067957	-0.798508
H	-3.328044	-2.607383	0.666164
H	-7.252599	0.047227	-0.754375
H	-5.562731	-3.625297	0.694888
O	-2.309383	1.258984	-1.907068
O	-2.152406	2.604102	-2.358379
H	-1.191069	2.711670	-2.398505

ENERGIES
SCF ENERGY: -1527.93010135
SUM OF ELECTRONIC AND ZERO-POINT ENERGIES: -1527.519054
SUM OF ELECTRONIC AND THERMAL ENERGIES: -1527.490484
SUM OF ELECTRONIC AND THERMAL ENTHALPIES: -1527.489540
SUM OF ELECTRONIC AND THERMAL FREE ENERGIES: -1527.580624

==> AQ/PA/C4PP/RAF/C1P/TS <==
52
XYZ-COORDINATES + ENERGIES

O	-1.984474	-1.341688	0.152697
O	-5.902651	-1.374281	1.305033
O	-5.263249	-1.924369	-1.340352
O	-2.431630	0.915027	0.542462
O	-3.416867	-1.289668	3.025937
O	-3.307945	-1.129332	-3.225899
O	-0.586527	4.789685	-1.330103
O	7.779616	-2.582840	-0.184030
C	-4.344456	-1.170598	-0.556939
C	-4.552839	-1.549181	0.893716
C	-2.904723	-1.526608	-0.939189
C	-3.688218	-0.666598	1.776285
C	-2.338426	-0.378611	1.114098
C	-2.424828	-0.761994	-2.158937
C	-1.278960	1.532182	0.108213
C	-0.019996	0.953266	0.169816
C	-1.470654	2.813558	-0.403734
C	1.085224	1.684569	-0.291374
C	-0.367094	3.526635	-0.841384
C	0.910405	2.979038	-0.780230
C	2.444583	1.148226	-0.232133
C	2.742776	-0.189544	-0.214040
C	4.050292	-0.761373	-0.201985
C	5.244644	0.001644	-0.180621
C	4.185598	-2.170166	-0.206241
C	6.477058	-0.598846	-0.174012
C	5.413542	-2.783982	-0.201368
C	6.624736	-2.024476	-0.187420
H	-4.253766	-2.592856	1.028903
H	-4.532868	-0.102063	-0.695748
H	-2.883672	-2.594540	-1.158427
H	-4.202550	0.282572	1.931939
H	-1.553342	-0.404236	1.866071
H	-1.397320	-1.044872	-2.391738
H	-2.478291	0.312248	-1.982723
H	-6.452637	-1.958918	0.772301
H	-5.053261	-1.741121	-2.265919
H	-4.259406	-1.482327	3.451470
H	-3.166669	-0.529956	-3.964318
H	0.118628	-0.038879	0.567519
H	-2.461508	3.243770	-0.442154
H	1.762130	3.552525	-1.124474

H	3.229329	1.867393	-0.424404
H	1.925362	-0.901784	-0.173738
H	0.249583	5.181700	-1.607236
H	5.190422	1.082649	-0.164288
H	3.284736	-2.773018	-0.216274
H	7.379192	0.000139	-0.155169
H	5.491275	-3.864068	-0.207184
O	2.767401	1.498495	1.981999
O	2.112841	2.660157	2.310446
H	1.200529	2.393291	2.494946

ENERGIES
SCF ENERGY: -1527.90311467
SUM OF ELECTRONIC AND ZERO-POINT ENERGIES: -1527.495744
SUM OF ELECTRONIC AND THERMAL ENERGIES: -1527.466228
SUM OF ELECTRONIC AND THERMAL ENTHALPIES: -1527.465284
SUM OF ELECTRONIC AND THERMAL FREE ENERGIES: -1527.559553

==> AQ/PA/C4PP/RAF/C1PP <==
52
XYZ-COORDINATES + ENERGIES

O	2.269125	-1.223053	0.131338
O	6.088694	-1.490011	-1.286021
O	5.668977	-1.248235	1.454430
O	2.563340	0.829345	-0.938567
O	3.496295	-1.969641	-2.748744
O	3.817131	0.046880	3.165747
O	0.616833	5.032414	-0.252548
O	-7.805791	-0.667790	-1.356189
C	4.662319	-0.783688	0.561605
C	4.783368	-1.569086	-0.727202
C	3.271507	-1.043522	1.149327
C	3.816471	-1.005301	-1.753294
C	2.501815	-0.577919	-1.096213
C	2.841310	0.043576	2.116793
C	1.406716	1.513442	-0.637418
C	0.182738	0.901353	-0.411453
C	1.555607	2.896949	-0.576521
C	-0.933188	1.702547	-0.133923
C	0.443297	3.672633	-0.299054
C	-0.800908	3.088611	-0.081586
C	-2.269522	1.137775	0.114338
C	-2.569332	-0.157817	0.212301
C	-3.961673	-0.695147	0.408466
C	-5.005979	0.303066	0.747305
C	-4.344725	-1.570472	-0.729333
C	-6.248265	0.269711	0.194256
C	-5.587301	-1.554607	-1.278162
C	-6.618114	-0.659352	-0.835788
H	4.526096	-2.613357	-0.527454
H	4.809418	0.282079	0.363819
H	3.318117	-1.994583	1.680479
H	4.270080	-0.126462	-2.213088
H	1.668875	-0.849606	-1.740253
H	1.849856	-0.183819	2.510764
H	2.825028	1.013285	1.619480
H	6.700773	-1.916735	-0.677065
H	5.517940	-0.802178	2.298323
H	4.315435	-2.231572	-3.183237
H	3.687896	0.833378	3.703593
H	0.086242	-0.171414	-0.442239
H	2.519724	3.351445	-0.756368
H	-1.659892	3.712836	0.131615
H	-3.061644	1.871758	0.213871
H	-1.810288	-0.925476	0.103373
H	-0.229045	5.461668	-0.079483
H	-4.762317	1.033105	1.509275
H	-3.584716	-2.250320	-1.095372
H	-6.999797	0.984173	0.511933
H	-5.826886	-2.226968	-2.094547
O	-3.725434	-1.560337	1.646029
O	-4.913507	-2.269470	1.965849
H	-5.523066	-1.568570	2.245334

ENERGIES
SCF ENERGY: -1527.89930380
SUM OF ELECTRONIC AND ZERO-POINT ENERGIES: -1527.490108
SUM OF ELECTRONIC AND THERMAL ENERGIES: -1527.460929
SUM OF ELECTRONIC AND THERMAL ENTHALPIES: -1527.459985
SUM OF ELECTRONIC AND THERMAL FREE ENERGIES: -1527.552623

==> AQ/PA/C4PP/RAF/C2 <==
52
XYZ-COORDINATES + ENERGIES

O	1.939837	-1.123263	0.688757
O	5.496418	-2.293194	-0.946248
O	5.453629	-1.296686	1.650028
O	2.178671	0.495985	-0.972761
O	2.787696	-2.679846	-2.089071
O	3.992888	0.647719	3.109515
O	0.681312	4.837299	0.006557
O	-8.216156	-2.071621	0.839199
C	4.404406	-0.937031	0.758887
C	4.268900	-2.029611	-0.279013
C	3.079423	-0.826456	1.517783
C	3.274566	-1.579368	-1.330868
C	2.062416	-0.895006	-0.691680
C	2.913138	0.530375	2.174472
C	1.125764	1.316769	-0.631016
C	-0.260915	0.759401	-0.642461
C	1.385651	2.625093	-0.439779
C	-1.300707	1.732206	-0.168149
C	0.320334	3.525356	-0.151153
C	-0.977557	3.080253	-0.002856
C	-2.629236	1.275941	0.069128
C	-3.064347	-0.011581	-0.050477
C	-4.406346	-0.496732	0.193148
C	-5.491056	0.324932	0.561208
C	-4.671351	-1.872142	0.054492
C	-6.750398	-0.193859	0.776502
C	-5.927554	-2.404185	0.268728
C	-7.029327	-1.585572	0.639848
H	3.902300	-2.939948	0.203923
H	4.647809	0.010092	0.266528
H	3.081403	-1.597403	2.288936
H	3.768623	-0.860904	-1.986636
H	1.156993	-1.297453	-1.135325
H	1.950432	0.581719	2.685068
H	2.976165	1.322418	1.429433
H	6.110537	-2.668693	-0.306459
H	5.454131	-0.636740	2.356476
H	3.542257	-3.103149	-2.512903
H	4.063833	1.566234	3.384727
H	2.400990	2.995246	-0.490234
H	-1.753138	3.783437	0.278674
H	-3.330920	2.044085	0.376006
H	-2.360156	-0.776037	-0.359319
H	-0.095635	5.366126	0.222968
H	-5.343493	1.391392	0.677290
H	-3.857182	-2.529943	-0.229158
H	-7.567073	0.461102	1.056170
H	-6.097285	-3.468024	0.153685
H	-0.321572	-0.171315	-0.075389
O	-0.563214	0.264409	-1.992151
O	-0.577704	1.358792	-2.895280
H	0.329156	1.378304	-3.233281

ENERGIES
SCF ENERGY: -1527.90805880
SUM OF ELECTRONIC AND ZERO-POINT ENERGIES: -1527.498543
SUM OF ELECTRONIC AND THERMAL ENERGIES: -1527.469407
SUM OF ELECTRONIC AND THERMAL ENTHALPIES: -1527.468463
SUM OF ELECTRONIC AND THERMAL FREE ENERGIES: -1527.561298

==> AQ/PA/C4PP/RAF/C2/TS <==
52
XYZ-COORDINATES + ENERGIES

O	-1.816102	-1.316737	-0.360663
O	-5.587374	-1.999452	1.039783
O	-5.193145	-1.667932	-1.689791

O	-2.255063	0.678669	0.761265
O	-3.013336	-2.164533	2.550021
O	-3.473837	-0.160084	-3.381435
O	-0.633037	4.882648	-0.489833
O	8.089511	-2.260410	-0.486352
C	-4.240938	-1.114771	-0.788731
C	-4.278751	-1.924373	0.489913
C	-2.831091	-1.216648	-1.377869
C	-3.391010	-1.256347	1.523028
C	-2.102822	-0.730997	0.883002
C	-2.517070	-0.068980	-2.319249
C	-1.168752	1.432070	0.397750
C	0.153260	0.900981	0.391147
C	-1.411731	2.757201	0.141565
C	1.209202	1.734481	-0.109157
C	-0.340424	3.577849	-0.238557
C	0.948673	3.075055	-0.375186
C	2.552711	1.228053	-0.285937
C	2.969383	-0.020017	0.029144
C	4.306570	-0.556133	-0.124876
C	5.394421	0.173434	-0.644547
C	4.556009	-1.884178	0.265183
C	6.646383	-0.388345	-0.765227
C	5.805102	-2.460222	0.149444
C	6.912142	-1.735072	-0.372967
H	-3.901323	-2.930349	0.284946
H	-4.498318	-0.072790	-0.574950
H	-2.777301	-2.154736	-1.930750
H	-3.931716	-0.410378	1.949463
H	-1.259597	-0.965533	1.526307
H	-1.500584	-0.172116	-2.701931
H	-2.620625	0.886483	-1.805369
H	-6.140800	-2.493476	0.425481
H	-5.090400	-1.195602	-2.526561
H	-3.815550	-2.464777	2.991254
H	-3.437672	0.650082	-3.897890
H	-2.413602	3.158019	0.204804
H	1.745007	3.727758	-0.710955
H	3.258974	1.942683	-0.692465
H	2.257056	-0.720419	0.452419
H	0.162616	5.351573	-0.769675
H	5.253795	1.200466	-0.957767
H	3.736231	-2.468138	0.668752
H	7.467623	0.193014	-1.166904
H	5.965970	-3.485839	0.458739
H	0.277014	-0.167138	0.315264
O	0.499982	0.809786	2.276817
O	0.469493	2.098290	2.808752
H	-0.461136	2.251003	3.022045

ENERGIES
SCF ENERGY: -1527.89450335
SUM OF ELECTRONIC AND ZERO-POINT ENERGIES: -1527.486081
SUM OF ELECTRONIC AND THERMAL ENERGIES: -1527.456976
SUM OF ELECTRONIC AND THERMAL ENTHALPIES: -1527.456032
SUM OF ELECTRONIC AND THERMAL FREE ENERGIES: -1527.549267

==> AQ/PA/C4PP/RAF/C2P <==
52
XYZ-COORDINATES + ENERGIES

O	2.055299	-1.225073	0.201931
O	5.912697	-1.716035	-1.041412
O	5.409483	-1.168947	1.637516
O	2.443533	0.694607	-1.068189
O	3.346566	-2.319976	-2.520922
O	3.525495	0.310730	3.148634
O	0.633766	5.004143	-0.774395
O	-8.153666	-1.609656	-0.231652
C	4.441376	-0.787302	0.665993
C	4.587955	-1.710381	-0.525062
C	3.027758	-0.960617	1.229994
C	3.665722	-1.249851	-1.639825
C	2.344589	-0.719503	-1.077861
C	2.588928	0.226359	2.067597
C	1.301908	1.435085	-0.853240
C	0.061103	0.882246	-0.595376
C	1.493817	2.815828	-0.907544
C	-1.046863	1.741061	-0.392649
C	0.401958	3.652386	-0.706044
C	-0.856767	3.138987	-0.449731
C	-2.344860	1.238933	-0.126504
C	-2.665272	-0.213795	-0.054690
C	-4.129195	-0.536519	-0.106991
C	-5.098300	0.200679	0.585071
C	-4.562264	-1.651220	-0.832857
C	-6.435878	-0.157317	0.552535
C	-5.896534	-2.016288	-0.880851
C	-6.894691	-1.280762	-0.187020
H	4.302327	-2.721444	-0.220358
H	4.615934	0.248218	0.360271
H	3.037327	-1.853005	1.856457
H	4.154231	-0.442744	-2.187269
H	1.523927	-1.036468	-1.717394
H	1.577887	0.059827	2.442161
H	2.615025	1.142666	1.477741
H	6.493348	-2.083987	-0.366732
H	5.239957	-0.631845	2.422985
H	4.172285	-2.680324	-2.862111
H	3.391781	1.143093	3.611050
H	-0.067432	-0.184514	-0.523254
H	2.474332	3.223575	-1.108181
H	-1.694610	3.806579	-0.292628
H	-3.136192	1.957111	0.028341
H	-0.193727	5.482093	-0.646211
H	-4.807042	1.071059	1.160769
H	-3.831171	-2.238390	-1.377871
H	-7.167717	0.429096	1.095160
H	-6.205555	-2.877860	-1.460559
H	-2.155317	-0.758876	-0.851948
O	-2.021590	-0.832747	1.115751
O	-2.512856	-0.220711	2.297822
H	-3.356358	-0.672609	2.449245

ENERGIES
SCF ENERGY: -1527.92347529
SUM OF ELECTRONIC AND ZERO-POINT ENERGIES: -1527.513022
SUM OF ELECTRONIC AND THERMAL ENERGIES: -1527.484222
SUM OF ELECTRONIC AND THERMAL ENTHALPIES: -1527.483278
SUM OF ELECTRONIC AND THERMAL FREE ENERGIES: -1527.575897

==> AQ/PA/C4PP/RAF/C2P/TS <==
52
XYZ-COORDINATES + ENERGIES

O	1.958593	-1.234428	-0.072024
O	5.900700	-1.700464	-1.011258
O	5.140960	-1.634094	1.665099
O	2.534496	0.870022	-0.907235
O	3.498518	-1.845651	-2.826296
O	3.157760	-0.354613	3.242995
O	0.797202	5.151828	-0.141168
O	-7.898319	-1.954072	-0.256087
C	4.295803	-1.022621	0.696795
C	4.530676	-1.711576	-0.631186
C	2.824753	-1.218837	1.077548
C	3.751878	-0.991137	-1.717662
C	2.395089	-0.511686	-1.195973
C	2.340700	-0.186087	2.078325
C	1.398392	1.613831	-0.682451
C	0.126223	1.071656	-0.602317
C	1.623093	2.980208	-0.525208
C	-0.963767	1.927774	-0.360281
C	0.539911	3.812725	-0.286757
C	-0.748031	3.302177	-0.199106
C	-2.317287	1.436245	-0.236127
C	-2.699703	0.113087	-0.418615
C	-4.063417	-0.360524	-0.397519
C	-5.159637	0.410533	0.038605
C	-4.318448	-1.695851	-0.765883
C	-6.432336	-0.113069	0.082849
C	-5.585510	-2.233717	-0.722842

C	-6.707090	-1.461968	-0.296934
H	4.173273	-2.742866	-0.558474
H	4.539878	0.040506	0.614223
H	2.735792	-2.211234	1.520431
H	4.323865	-0.119052	-2.037140
H	1.634691	-0.659368	-1.959566
H	1.289864	-0.363590	2.312313
H	2.462210	0.820918	1.679161
H	6.389297	-2.237417	-0.378426
H	4.911122	-1.244454	2.519167
H	4.346222	-2.159376	-3.160036
H	3.005678	0.387141	3.835485
H	-0.025449	0.008730	-0.686379
H	2.625217	3.379645	-0.592094
H	-1.581724	3.964564	-0.002681
H	-3.055879	2.164825	0.069467
H	-0.028930	5.629229	-0.001587
H	-5.010844	1.437502	0.346848
H	-3.486708	-2.310330	-1.092333
H	-7.261879	0.497520	0.417745
H	-5.756047	-3.262586	-1.014903
H	-1.993267	-0.567176	-0.874156
O	-1.973986	-0.516765	1.444127
O	-3.064616	-0.552993	2.298225
H	-3.634611	-1.250252	1.940339

ENERGIES
SCF ENERGY: -1527.89929928
SUM OF ELECTRONIC AND ZERO-POINT ENERGIES: -1527.491378
SUM OF ELECTRONIC AND THERMAL ENERGIES: -1527.462226
SUM OF ELECTRONIC AND THERMAL ENTHALPIES: -1527.461282
SUM OF ELECTRONIC AND THERMAL FREE ENERGIES: -1527.553694

==> AQ/PA/C4PP/RAF/C2PP <==
52
XYZ-COORDINATES + ENERGIES

O	2.304403	-1.244930	0.295595
O	6.235777	-1.677455	-0.712689
O	5.566095	-1.060965	1.918242
O	2.703823	0.641382	-1.018340
O	3.791421	-2.371334	-2.328957
O	3.568709	0.451568	3.252469
O	0.750611	4.894508	-0.933956
O	-7.691944	-2.428630	-0.232510
C	4.649764	-0.731180	0.879938
C	4.884158	-1.686569	-0.270973
C	3.208964	-0.915881	1.366709
C	4.020274	-1.276609	-1.450230
C	2.651621	-0.774748	-0.983239
C	2.696205	0.293321	2.127050
C	1.530148	1.352207	-0.897787
C	0.286632	0.767637	-0.719425
C	1.687417	2.735017	-0.966055
C	-0.846343	1.594232	-0.618894
C	0.563399	3.537766	-0.858480
C	-0.698781	2.983587	-0.683832
C	-2.190961	1.064945	-0.443063
C	-2.534861	-0.257252	-0.447346
C	-3.846223	-0.762986	-0.287311
C	-5.005049	0.146146	0.005063
C	-4.073598	-2.153293	-0.320082
C	-6.346087	-0.472385	-0.126053
C	-5.333109	-2.678315	-0.232674
C	-6.534594	-1.831548	-0.180085
H	4.602196	-2.694613	0.046580
H	4.821667	0.297859	0.551077
H	3.200505	-1.782267	2.028583
H	4.518887	-0.465183	-1.981856
H	1.881119	-1.142142	-1.656906
H	1.670788	0.115809	2.454118
H	2.731849	1.183763	1.499604
H	6.785981	-2.023540	-0.002046
H	5.344313	-0.498513	2.672138
H	4.647803	-2.687357	-2.637035
H	3.398325	1.306973	3.657499
H	0.186734	-0.301778	-0.637143
H	2.668352	3.167461	-1.103929
H	-1.564834	3.628575	-0.602252
H	-2.963115	1.811966	-0.309196
H	-1.765289	-1.006479	-0.596093
H	-0.099575	5.344567	-0.870571
H	-3.218695	-2.808869	-0.436971
H	-7.208562	0.182548	-0.144782
H	-5.485514	-3.749564	-0.254278
H	-4.936734	1.075972	-0.560553
O	-4.724947	0.553957	1.415925
O	-5.674388	1.543958	1.786485
H	-6.466522	1.023776	1.986506

ENERGIES
SCF ENERGY: -1527.90380494
SUM OF ELECTRONIC AND ZERO-POINT ENERGIES: -1527.494085
SUM OF ELECTRONIC AND THERMAL ENERGIES: -1527.465124
SUM OF ELECTRONIC AND THERMAL ENTHALPIES: -1527.464180
SUM OF ELECTRONIC AND THERMAL FREE ENERGIES: -1527.556540

==> AQ/PA/C4PP/RAF/C3 <==
52
XYZ-COORDINATES + ENERGIES

O	1.129336	-1.315599	-0.553417
O	4.930331	-2.672692	0.041348
O	3.073348	-2.889124	2.102452
O	2.375109	0.651603	-0.411448
O	3.571339	-1.839047	-2.537382
O	0.848693	-1.442037	3.071416
O	1.337336	4.276656	2.041792
O	-7.931575	-1.682134	-0.371218
C	2.869314	-1.902133	1.096179
C	3.555061	-2.375768	-0.168279
C	1.374152	-1.753153	0.794685
C	3.476251	-1.289657	-1.227445
C	2.145097	-0.536059	-1.145335
C	0.671681	-0.838716	1.782108
C	1.526578	1.767510	-0.697669
C	0.074009	1.424120	-0.748171
C	1.887638	2.812437	0.297805
C	-0.832074	1.943446	0.137972
C	0.950686	3.328851	1.123750
C	-0.406378	2.912537	1.077360
C	-2.242315	1.533547	0.162585
C	-2.687159	0.355285	-0.305534
C	-4.062903	-0.127366	-0.318037
C	-5.167746	0.643268	0.084637
C	-4.321635	-1.437443	-0.748582
C	-6.449493	0.131527	0.067528
C	-5.600780	-1.963372	-0.771778
C	-6.725305	-1.199960	-0.359352
H	3.034988	-3.266429	-0.532883
H	3.299669	-0.951264	1.422799
H	0.934292	-2.749090	0.861548
H	4.284269	-0.576368	-1.060113
H	1.796320	-0.307853	-2.149410
H	-0.388298	-0.769802	1.532426
H	1.117926	0.155387	1.767775
H	4.984245	-3.435158	0.627234
H	2.541408	-2.619694	2.863362
H	4.400440	-2.326451	-2.595941
H	0.590584	-0.807975	3.746542
H	-0.232628	0.749730	-1.534349
H	2.922650	3.114565	0.365250
H	-1.117604	3.345994	1.767830
H	-2.924542	2.233662	0.631228
H	-1.960421	-0.344894	-0.708936
H	0.578975	4.558851	2.565202
H	-5.019520	1.664318	0.414665
H	-3.488367	-2.052814	-1.069930
H	-7.284437	0.746518	0.381791
H	-5.767411	-2.979422	-1.108860
O	1.794008	2.191204	-2.053037
O	3.164588	2.538886	-2.191054

H	3.149293	3.503044	-2.109411

\# ENERGIES
SCF ENERGY: -1527.89874022
SUM OF ELECTRONIC AND ZERO-POINT ENERGIES: -1527.489927
SUM OF ELECTRONIC AND THERMAL ENERGIES: -1527.461009
SUM OF ELECTRONIC AND THERMAL ENTHALPIES: -1527.460065
SUM OF ELECTRONIC AND THERMAL FREE ENERGIES: -1527.551272

==> AQ/PA/C4PP/RAF/C3PP <==
52
XYZ-COORDINATES + ENERGIES

O	2.427092	-1.240747	0.147631
O	6.343848	-1.759756	-0.859895
O	5.656581	-1.483616	1.819784
O	2.925019	0.790414	-0.884578
O	3.926682	-2.025498	-2.612121
O	3.721835	-0.049552	3.328924
O	1.114814	5.055608	-0.202683
O	-7.513490	-2.184986	-0.095263
C	4.783791	-0.950359	0.829979
C	4.984095	-1.741440	-0.445041
C	3.324085	-1.111677	1.266148
C	4.178451	-1.101778	-1.560191
C	2.820366	-0.613530	-1.047908
C	2.869229	0.012287	2.178783
C	1.774450	1.521635	-0.681246
C	0.505280	0.966045	-0.626159
C	1.982887	2.891144	-0.533944
C	-0.598663	1.809087	-0.427685
C	0.883627	3.709953	-0.339685
C	-0.402868	3.183138	-0.287494
C	-1.976702	1.306992	-0.349837
C	-2.350439	0.020657	-0.397106
C	-3.728795	-0.482041	-0.321526
C	-4.834561	0.332653	-0.361717
C	-3.892439	-1.892888	-0.234991
C	-6.208739	-0.205129	-0.252928
C	-5.154467	-2.480825	-0.175809
C	-6.317298	-1.710213	-0.192093
H	4.629279	-2.763787	-0.285514
H	5.024248	0.102745	0.655719
H	3.250265	-2.057019	1.804370
H	4.731537	-0.241374	-1.939326
H	2.051606	-0.853941	-1.778185
H	1.826291	-0.138243	2.460814
H	2.980374	0.976913	1.683838
H	6.850602	-2.241094	-0.197259
H	5.442938	-1.034868	2.648563
H	4.776417	-2.338647	-2.941165
H	3.621395	0.763428	3.832253
H	0.364338	-0.097855	-0.722435
H	2.982054	3.300830	-0.579183
H	-1.248370	3.841775	-0.131715
H	-2.729298	2.078859	-0.233938
H	-1.596360	-0.753464	-0.488402
H	0.277032	5.519233	-0.089177
H	-4.741646	1.402593	-0.488246
H	-3.007658	-2.515384	-0.213786
H	-5.257000	-3.556585	-0.103130
H	-6.877654	0.190975	-1.023436
O	-6.772056	0.312361	1.018249
O	-8.196597	0.140227	0.988349
H	-8.267956	-0.803007	0.704864

\# ENERGIES
SCF ENERGY: -1527.90922458
SUM OF ELECTRONIC AND ZERO-POINT ENERGIES: -1527.498554
SUM OF ELECTRONIC AND THERMAL ENERGIES: -1527.469965
SUM OF ELECTRONIC AND THERMAL ENTHALPIES: -1527.469021
SUM OF ELECTRONIC AND THERMAL FREE ENERGIES: -1527.560321

==> AQ/PA/C4PP/RAF/C3PP/TS <==
52
XYZ-COORDINATES + ENERGIES

O	2.374438	-1.245984	0.183833
O	6.285303	-1.875729	-0.785515
O	5.586010	-1.534213	1.884162
O	2.929405	0.750403	-0.886126
O	3.872297	-2.126951	-2.545966
O	3.676219	-0.022612	3.350007
O	1.222193	5.068904	-0.280469
O	-7.586123	-1.903100	-0.496935
C	4.733146	-0.998925	0.878027
C	4.923749	-1.819413	-0.379756
C	3.266850	-1.117119	1.306270
C	4.139983	-1.185733	-1.513714
C	2.791222	-0.653767	-1.021451
C	2.831673	0.034448	2.193769
C	1.795810	1.513086	-0.706272
C	0.513000	0.989964	-0.652446
C	2.037015	2.879215	-0.577959
C	-0.571285	1.863988	-0.479225
C	0.957688	3.728161	-0.402306
C	-0.342034	3.234427	-0.352795
C	-1.962013	1.399937	-0.414448
C	-2.377511	0.132071	-0.560527
C	-3.762682	-0.333236	-0.499366
C	-4.856092	0.499344	-0.341217
C	-3.987430	-1.733396	-0.658382
C	-6.162608	-0.031077	-0.221591
C	-5.247444	-2.271857	-0.667092
C	-6.394081	-1.445199	-0.497770
H	4.545448	-2.830216	-0.201920
H	5.000224	0.044528	0.685648
H	3.166857	-2.050102	1.861523
H	4.715838	-0.347680	-1.908659
H	2.021796	-0.890437	-1.752314
H	1.784151	-0.087927	2.472366
H	2.966325	0.986336	1.680387
H	6.777230	-2.358127	-0.112653
H	5.381280	-1.061768	2.701838
H	4.716223	-2.470561	-2.859171
H	3.581922	0.797581	3.842727
H	0.347636	-0.072346	-0.723678
H	3.046614	3.262905	-0.619366
H	-1.171725	3.916534	-0.213591
H	-2.688246	2.184002	-0.233015
H	-1.650113	-0.651478	-0.740928
H	0.396138	5.555635	-0.178169
H	-4.730882	1.570986	-0.266563
H	-3.127189	-2.377806	-0.792753
H	-5.401389	-3.332798	-0.816255
H	-7.009077	0.627218	-0.366910
O	-6.512460	-0.169687	1.648558
O	-7.915444	-0.368351	1.777501
H	-8.085499	-1.105421	1.158565

\# ENERGIES
SCF ENERGY: -1527.89970653
SUM OF ELECTRONIC AND ZERO-POINT ENERGIES: -1527.490462
SUM OF ELECTRONIC AND THERMAL ENERGIES: -1527.461666
SUM OF ELECTRONIC AND THERMAL ENTHALPIES: -1527.460722
SUM OF ELECTRONIC AND THERMAL FREE ENERGIES: -1527.552478

==> AQ/PA/C4PP/RAF/C4 <==
52
XYZ-COORDINATES + ENERGIES

O	1.512469	-1.712580	0.075515
O	5.406275	-2.259375	-1.020022
O	4.737794	-2.284737	1.681010
O	2.124545	0.401628	-0.696440
O	2.944274	-2.256810	-2.756520
O	2.863433	-0.993150	3.372928
O	0.692013	4.656669	0.702522
O	-8.495438	-1.817482	0.023021
C	3.882291	-1.605922	0.768211
C	4.050134	-2.246209	-0.593562
C	2.419092	-1.768356	1.192973
C	3.258629	-1.458374	-1.622151

C	1.934802	-0.962847	-1.035685
C	2.008204	-0.757616	2.248190
C	1.022495	1.155931	-0.383111
C	-0.252377	0.721186	-0.328908
C	1.432432	2.558004	-0.094208
C	-1.320778	1.607639	0.048736
C	0.292401	3.387202	0.376149
C	-0.983077	2.952569	0.408261
C	-2.676307	1.204110	0.106577
C	-3.175719	-0.034420	-0.210752
C	-4.556348	-0.446440	-0.136066
C	-5.611192	0.384305	0.299592
C	-4.900846	-1.760016	-0.514572
C	-6.912619	-0.066560	0.351312
C	-6.200052	-2.222447	-0.466406
C	-7.270697	-1.393878	-0.028819
H	3.663184	-3.268455	-0.550204
H	4.160300	-0.549010	0.720268
H	2.311701	-2.773188	1.602355
H	3.845346	-0.589545	-1.923534
H	1.145569	-1.056002	-1.778416
H	0.962274	-0.909626	2.518169
H	2.147471	0.258733	1.879362
H	5.900101	-2.833475	-0.424990
H	4.552444	-1.920619	2.556831
H	3.770934	-2.576847	-3.134266
H	2.772804	-0.258683	3.986574
H	-0.475624	-0.314887	-0.529017
H	2.272145	2.580173	0.605412
H	-1.769790	3.628106	0.724321
H	-3.367179	1.971212	0.438928
H	-2.493886	-0.803229	-0.556013
H	-0.076109	5.194444	0.931815
H	-5.404013	1.402352	0.604279
H	-4.112764	-2.423289	-0.853591
H	-7.702440	0.592937	0.691003
H	-6.429799	-3.238554	-0.763993
O	1.948899	3.108790	-1.349366
O	2.926845	4.097671	-1.061159
H	2.395649	4.875301	-0.831776

ENERGIES
SCF ENERGY: -1527.91041915
SUM OF ELECTRONIC AND ZERO-POINT ENERGIES: -1527.500257
SUM OF ELECTRONIC AND THERMAL ENERGIES: -1527.471143
SUM OF ELECTRONIC AND THERMAL ENTHALPIES: -1527.470199
SUM OF ELECTRONIC AND THERMAL FREE ENERGIES: -1527.562899

==> AQ/PA/C4PP/RAF/C4/TS <==
52
XYZ-COORDINATES + ENERGIES

O	1.563503	-1.696019	0.052486
O	5.434264	-2.163994	-1.157920
O	4.821689	-2.372338	1.547879
O	2.147130	0.468522	-0.590361
O	2.945508	-2.030628	-2.843644
O	2.984624	-1.212000	3.363209
O	0.683012	4.569035	1.092590
O	-8.410177	-1.954160	-0.129297
C	3.947515	-1.634611	0.701116
C	4.086964	-2.179146	-0.704697
C	2.493928	-1.828935	1.143911
C	3.277282	-1.318931	-1.657888
C	1.961218	-0.874244	-1.014933
C	2.106568	-0.895663	2.276269
C	1.049571	1.190988	-0.204366
C	-0.237176	0.724402	-0.188433
C	1.365952	2.534710	0.139486
C	-1.286754	1.575013	0.249471
C	0.319302	3.325979	0.672829
C	-0.979869	2.876937	0.690304
C	-2.667700	1.157491	0.282928
C	-3.141186	-0.043589	-0.134259
C	-4.514380	-0.489973	-0.115653
C	-5.593355	0.287368	0.355607
C	-4.815793	-1.779732	-0.593820
C	-6.882639	-0.193894	0.350432
C	-6.102372	-2.274894	-0.604433
C	-7.200108	-1.501878	-0.129416
H	3.697914	-3.201253	-0.725646
H	4.222998	-0.576001	0.720339
H	2.395033	-2.860254	1.483586
H	3.856970	-0.427893	-1.902494
H	1.161577	-0.925987	-1.749923
H	1.066053	-1.065335	2.556385
H	2.241447	0.144235	1.978886
H	5.940136	-2.772957	-0.609380
H	4.648184	-2.073994	2.450789
H	3.767131	-2.297666	-3.270126
H	2.921876	-0.515410	4.022916
H	-0.453603	-0.293173	-0.468517
H	2.390896	2.786875	0.369871
H	-1.768381	3.523098	1.055595
H	-3.358230	1.894905	0.675236
H	-2.444533	-0.772078	-0.534997
H	-0.095291	5.057797	1.387306
H	-5.412746	1.286401	0.732265
H	-4.004424	-2.397315	-0.962679
H	-7.695576	0.420671	0.717868
H	-6.303274	-3.271675	-0.977674
O	1.548676	3.322508	-1.652135
O	2.332094	4.472262	-1.525565
H	1.701446	5.167124	-1.294261

ENERGIES
SCF ENERGY: -1527.89272767
SUM OF ELECTRONIC AND ZERO-POINT ENERGIES: -1527.484333
SUM OF ELECTRONIC AND THERMAL ENERGIES: -1527.455090
SUM OF ELECTRONIC AND THERMAL ENTHALPIES: -1527.454146
SUM OF ELECTRONIC AND THERMAL FREE ENERGIES: -1527.547781

==> AQ/PA/C4PP/RAF/C4PP <==
52
XYZ-COORDINATES + ENERGIES

O	2.337352	-1.276078	0.102977
O	6.211519	-1.996574	-0.947115
O	5.553964	-1.779232	1.743626
O	2.968774	0.755986	-0.853620
O	3.765321	-2.059951	-2.689182
O	3.712930	-0.292959	3.320760
O	1.476606	5.114217	-0.025400
O	-7.476929	-1.103745	-1.064033
C	4.709968	-1.158346	0.780230
C	4.856874	-1.913529	-0.523683
C	3.245244	-1.248237	1.219909
C	4.084912	-1.188899	-1.611127
C	2.765691	-0.630244	-1.070255
C	2.861681	-0.136525	2.179071
C	1.874481	1.557559	-0.613495
C	0.572182	1.089005	-0.546668
C	2.179052	2.905400	-0.434647
C	-0.469577	2.002024	-0.303698
C	1.143796	3.793961	-0.193115
C	-0.173963	3.358070	-0.125912
C	-1.864958	1.598170	-0.214831
C	-2.343433	0.330921	-0.401793
C	-3.699324	-0.069570	-0.308387
C	-4.776844	0.827450	0.026409
C	-4.029735	-1.445254	-0.563195
C	-6.049556	0.396042	0.103135
C	-5.295250	-1.906173	-0.493121
C	-6.461195	-1.019410	-0.183221
H	4.443303	-2.918285	-0.397123
H	5.012950	-0.116026	0.643266
H	3.116301	-2.208476	1.720015
H	4.688993	-0.354111	-1.968957
H	1.976910	-0.786736	-1.802149
H	1.812728	-0.237176	2.461045
H	3.026717	0.838747	1.721296
H	6.694833	-2.516449	-0.296074

H	5.375808	-1.346819	2.589302
H	4.590698	-2.403867	-3.048198
H	3.638980	0.492852	3.869713
H	0.358336	0.039333	-0.658756
H	3.203139	3.246584	-0.489137
H	-0.967800	4.069658	0.065280
H	-2.554008	2.399730	0.017527
H	-1.647526	-0.462517	-0.648897
H	0.678739	5.636084	0.116949
H	-4.557785	1.868785	0.219140
H	-3.221637	-2.115381	-0.832006
H	-6.855116	1.076280	0.352057
H	-5.532701	-2.941067	-0.705085
O	-6.946262	-1.595233	1.150025
O	-8.292684	-1.143202	1.310276
H	-8.546867	-1.101465	0.354662

ENERGIES
SCF ENERGY: -1527.90791673
SUM OF ELECTRONIC AND ZERO-POINT ENERGIES: -1527.498438
SUM OF ELECTRONIC AND THERMAL ENERGIES: -1527.469704
SUM OF ELECTRONIC AND THERMAL ENTHALPIES: -1527.468760
SUM OF ELECTRONIC AND THERMAL FREE ENERGIES: -1527.560476

==> AQ/PA/C4PP/RAF/C4PP/TS <==
52
XYZ-COORDINATES + ENERGIES

O	2.330116	-1.275489	0.193647
O	6.111391	-2.071513	-1.121126
O	5.673457	-1.680526	1.596151
O	2.900094	0.686425	-0.932691
O	3.502346	-2.274336	-2.625540
O	3.957773	-0.117646	3.230565
O	1.485539	5.116849	-0.412873
O	-7.470741	-1.311782	1.236062
C	4.750147	-1.124528	0.666470
C	4.795750	-1.963194	-0.593182
C	3.326815	-1.189525	1.229299
C	3.928189	-1.319891	-1.660181
C	2.666366	-0.706904	-1.047669
C	3.016573	-0.027347	2.154395
C	1.830338	1.516864	-0.682921
C	0.535314	1.069036	-0.475945
C	2.149138	2.872786	-0.653792
C	-0.482367	2.011392	-0.250779
C	1.136530	3.790629	-0.427950
C	-0.175305	3.374730	-0.229360
C	-1.872049	1.627549	-0.030016
C	-2.339023	0.351619	0.038229
C	-3.696258	-0.033469	0.252751
C	-4.002511	-1.427228	0.328714
C	-4.769184	0.903928	0.414718
C	-5.267614	-1.870339	0.531987
C	-6.040474	0.486827	0.614358
C	-6.392642	-0.938055	0.654912
H	4.402766	-2.958356	-0.365478
H	5.033284	-0.092802	0.438711
H	3.243331	-2.121403	1.789194
H	4.497764	-0.523974	-2.141699
H	1.818190	-0.893875	-1.701718
H	1.994459	-0.114510	2.525419
H	3.137392	0.921283	1.631244
H	6.648961	-2.553409	-0.483530
H	5.565080	-1.190349	2.421872
H	4.286670	-2.683311	-3.007353
H	3.913869	0.692730	3.746108
H	0.308318	0.015952	-0.478461
H	3.167094	3.198212	-0.815535
H	-0.953476	4.107936	-0.056616
H	-2.558019	2.456658	0.089124
H	-1.643101	-0.471910	-0.072074
H	0.703221	5.660894	-0.267108
H	-3.187220	-2.133624	0.232146
H	-4.558574	1.963961	0.387819
H	-5.496650	-2.925524	0.602785
H	-6.851708	1.190600	0.747828
O	-6.941742	-1.039838	-1.262372
O	-8.261311	-1.576419	-1.158508
H	-8.353415	-1.620272	-0.173426

ENERGIES
SCF ENERGY: -1527.90256949
SUM OF ELECTRONIC AND ZERO-POINT ENERGIES: -1527.494492
SUM OF ELECTRONIC AND THERMAL ENERGIES: -1527.465775
SUM OF ELECTRONIC AND THERMAL ENTHALPIES: -1527.464831
SUM OF ELECTRONIC AND THERMAL FREE ENERGIES: -1527.556141

==> AQ/PA/C4PP/RAF/C5 <==
52
XYZ-COORDINATES + ENERGIES

O	1.727203	-1.612062	0.012250
O	5.626797	-2.143650	-1.067757
O	4.957791	-2.102660	1.633008
O	2.305351	0.486768	-0.829287
O	3.176344	-2.208874	-2.808813
O	3.061099	-0.792272	3.292351
O	0.674864	4.195739	1.260335
O	-8.040371	-2.607093	0.174979
C	4.094851	-1.458716	0.702175
C	4.270365	-2.133054	-0.642034
C	2.633726	-1.626319	1.131012
C	3.472530	-1.380255	-1.691401
C	2.137927	-0.890982	-1.122196
C	2.210698	-0.591580	2.157180
C	1.198561	1.250302	-0.518746
C	-0.105451	0.712412	-0.473434
C	1.449606	2.555770	-0.239071
C	-1.209627	1.542330	-0.101458
C	0.345462	3.495189	0.080698
C	-1.000260	2.860391	0.171675
C	-2.570445	1.003898	0.005741
C	-2.927127	-0.278667	-0.175633
C	-4.271430	-0.837142	-0.073791
C	-5.419291	-0.080609	0.221521
C	-4.451947	-2.213287	-0.280068
C	-6.667404	-0.664387	0.304530
C	-5.696535	-2.812062	-0.199680
C	-6.864195	-2.060516	0.097572
H	3.893906	-3.157742	-0.572341
H	4.360042	-0.400093	0.625695
H	2.537080	-2.620529	1.568020
H	4.048555	-0.510455	-2.009652
H	1.356291	-1.031108	-1.864643
H	1.164026	-0.743655	2.425050
H	2.345630	0.415781	1.763417
H	6.125006	-2.702699	-0.462180
H	4.765343	-1.720448	2.499541
H	4.010205	-2.525192	-3.173775
H	2.965847	-0.040487	3.884006
H	-0.270641	-0.327002	-0.695129
H	2.455681	2.949857	-0.257023
H	-1.821288	3.501764	0.464588
H	-3.322080	1.741944	0.261696
H	-2.166406	-1.011606	-0.423528
H	-0.113893	4.647246	1.585947
H	-5.334422	0.986206	0.388128
H	-3.584451	-2.822147	-0.510185
H	-7.536143	-0.058209	0.532466
H	-5.801353	-3.877644	-0.365138
O	0.382119	4.436497	-1.032279
O	-0.592741	5.448622	-0.812802
H	-0.084676	6.141807	-0.368401

ENERGIES
SCF ENERGY: -1527.89639655
SUM OF ELECTRONIC AND ZERO-POINT ENERGIES: -1527.486902
SUM OF ELECTRONIC AND THERMAL ENERGIES: -1527.457757
SUM OF ELECTRONIC AND THERMAL ENTHALPIES: -1527.456813
SUM OF ELECTRONIC AND THERMAL FREE ENERGIES: -1527.549337

==> AQ/PA/C4PP/RAF/C5PP <==
52
XYZ-COORDINATES + ENERGIES

O	2.180708	-1.269361	0.203702
O	5.972283	-2.236833	-0.966168
O	5.457153	-1.779043	1.729367
O	2.885402	0.640652	-0.936652
O	3.394568	-2.390890	-2.540983
O	3.749236	-0.127773	3.277675
O	1.690882	5.141862	-0.457210
O	-7.799060	-0.735328	0.696904
C	4.588000	-1.207265	0.757614
C	4.644930	-2.072889	-0.483257
C	3.144903	-1.208821	1.271578
C	3.834473	-1.425907	-1.592669
C	2.584829	-0.740680	-1.035243
C	2.848045	-0.022027	2.169283
C	1.854745	1.529339	-0.721016
C	0.531480	1.152061	-0.549535
C	2.246949	2.865329	-0.684377
C	-0.438022	2.145510	-0.345665
C	1.278582	3.833494	-0.482495
C	-0.058620	3.487375	-0.314533
C	-1.861081	1.834479	-0.150316
C	-2.395541	0.606521	-0.107722
C	-3.814249	0.285231	0.089042
C	-4.812246	1.297594	0.213640
C	-4.164227	-1.036948	0.181741
C	-6.145899	0.959675	0.433795
C	-5.562414	-1.479224	0.382883
C	-6.567937	-0.366092	0.537819
H	4.211196	-3.049100	-0.247557
H	4.917272	-0.191554	0.520737
H	3.006025	-2.128748	1.840256
H	4.450765	-0.669992	-2.081065
H	1.750132	-0.897731	-1.714139
H	1.811864	-0.066737	2.507320
H	3.019484	0.913189	1.636232
H	6.474234	-2.718949	-0.300436
H	5.339342	-1.269183	2.541781
H	4.170171	-2.854074	-2.875981
H	3.706799	0.686829	3.786726
H	0.247296	0.112725	-0.559974
H	3.284869	3.135257	-0.818904
H	-0.798681	4.262482	-0.157352
H	-2.495806	2.704250	-0.027662
H	-1.753974	-0.261058	-0.219157
H	0.932137	5.718691	-0.312944
H	-4.539766	2.339161	0.131768
H	-3.404659	-1.806086	0.118247
H	-6.897102	1.735832	0.517973
H	-5.661269	-2.200835	1.200408
O	-5.948121	-2.243903	-0.826040
O	-7.144190	-2.979616	-0.529673
H	-7.701425	-2.273034	-0.117056

ENERGIES
SCF ENERGY: -1527.90993402
SUM OF ELECTRONIC AND ZERO-POINT ENERGIES: -1527.499296
SUM OF ELECTRONIC AND THERMAL ENERGIES: -1527.470656
SUM OF ELECTRONIC AND THERMAL ENTHALPIES: -1527.469712
SUM OF ELECTRONIC AND THERMAL FREE ENERGIES: -1527.561395

==> AQ/PA/C4PP/RAF/C5PP/TS <==
52
XYZ-COORDINATES + ENERGIES

O	2.166399	-1.272449	0.171851
O	5.971656	-2.216740	-0.974971
O	5.428437	-1.802925	1.721992
O	2.884447	0.655694	-0.927843
O	3.405056	-2.353058	-2.574376
O	3.703306	-0.177935	3.278753
O	1.682994	5.151506	-0.417070
O	-7.734325	-0.759634	0.843705
C	4.568320	-1.216753	0.750823
C	4.639275	-2.062737	-0.502786
C	3.119957	-1.228506	1.250189
C	3.837948	-1.400856	-1.609996
C	2.583667	-0.723408	-1.053737
C	2.812349	-0.056569	2.163699
C	1.851796	1.541402	-0.710282
C	0.525874	1.162424	-0.562754
C	2.242587	2.876821	-0.652593
C	-0.446067	2.154092	-0.361918
C	1.270529	3.843689	-0.459759
C	-0.069348	3.496762	-0.319540
C	-1.869369	1.837166	-0.187340
C	-2.373399	0.611049	0.020274
C	-3.784454	0.271691	0.191266
C	-4.826799	1.240738	0.072261
C	-4.120888	-1.028133	0.515872
C	-6.136991	0.909511	0.292859
C	-5.474131	-1.433806	0.641814
C	-6.519014	-0.420747	0.635299
H	4.205355	-3.043287	-0.286252
H	4.898586	-0.197031	0.533494
H	2.976664	-2.157761	1.802353
H	4.457919	-0.638465	-2.083456
H	1.756287	-0.867980	-1.744177
H	1.773100	-0.108577	2.491188
H	2.986814	0.887142	1.646766
H	6.467681	-2.708769	-0.312041
H	5.302183	-1.306005	2.541118
H	4.182365	-2.815176	-2.906849
H	3.656419	0.629480	3.798704
H	0.238958	0.124313	-0.601942
H	3.282004	3.148704	-0.770761
H	-0.812629	4.270260	-0.169790
H	-2.528015	2.697346	-0.214416
H	-1.700492	-0.237341	0.084930
H	0.922004	5.727940	-0.283425
H	-4.581233	2.260801	-0.189032
H	-3.339083	-1.766446	0.643001
H	-6.917547	1.656091	0.219991
H	-5.698621	-2.351787	1.169040
O	-5.944019	-2.212890	-1.028947
O	-7.164386	-2.912182	-0.804811
H	-7.716302	-2.241520	-0.356616

ENERGIES
SCF ENERGY: -1527.90043289
SUM OF ELECTRONIC AND ZERO-POINT ENERGIES: -1527.490807
SUM OF ELECTRONIC AND THERMAL ENERGIES: -1527.462098
SUM OF ELECTRONIC AND THERMAL ENTHALPIES: -1527.461154
SUM OF ELECTRONIC AND THERMAL FREE ENERGIES: -1527.552296

==> AQ/PA/C4PP/RAF/C6 <==
52
XYZ-COORDINATES + ENERGIES

O	1.997561	-1.532662	-0.133728
O	5.956178	-1.502509	-1.132046
O	5.225887	-1.895844	1.526157
O	2.381932	0.707253	-0.659921
O	3.547070	-1.620039	-2.946430
O	3.168802	-1.020053	3.268176
O	0.428932	4.604409	1.070258
O	-7.914059	-2.829803	0.130087
C	4.319707	-1.227852	0.654713
C	4.598061	-1.697062	-0.757627
C	2.875902	-1.602039	1.004869
C	3.741899	-0.906484	-1.731517
C	2.356861	-0.624744	-1.145066
C	2.321376	-0.763830	2.142118
C	1.192686	1.301793	-0.313835
C	-0.036534	0.697779	-0.374896
C	1.366537	2.635418	0.187373
C	-1.195931	1.415168	-0.006439
C	0.286221	3.371636	0.498148
C	-1.100740	2.892048	0.215969
C	-2.495627	0.860670	0.093332

C	-2.845245	-0.440607	-0.164712
C	-4.165703	-1.011011	-0.070296
C	-5.320491	-0.286267	0.297908
C	-4.342788	-2.378657	-0.363955
C	-6.558364	-0.886773	0.366042
C	-5.577273	-2.990920	-0.298863
C	-6.747480	-2.269674	0.069012
H	4.338867	-2.757176	-0.832321
H	4.471074	-0.146847	0.725364
H	2.878179	-2.650225	1.304999
H	4.230267	0.048560	-1.929555
H	1.601487	-0.726228	-1.921216
H	1.293970	-1.062981	2.354061
H	2.349524	0.295627	1.887025
H	6.504381	-2.057902	-0.567522
H	4.973704	-1.652855	2.427076
H	4.413391	-1.798014	-3.328445
H	2.956356	-0.389985	3.962585
H	-0.122942	-0.342567	-0.645858
H	2.367205	3.015725	0.340884
H	-3.269350	1.553973	0.404609
H	-2.072622	-1.135478	-0.474757
H	-0.417874	5.067923	1.055034
H	-5.243080	0.767848	0.532735
H	-3.474432	-2.961647	-0.650080
H	-7.428764	-0.307248	0.649798
H	-5.678373	-4.044180	-0.531508
H	-1.792715	3.233814	0.988150
O	-1.581770	3.481978	-1.044141
O	-1.707242	4.891981	-0.856862
H	-0.943026	5.240618	-1.337116

ENERGIES
SCF ENERGY: -1527.90590557
SUM OF ELECTRONIC AND ZERO-POINT ENERGIES: -1527.495320
SUM OF ELECTRONIC AND THERMAL ENERGIES: -1527.466658
SUM OF ELECTRONIC AND THERMAL ENTHALPIES: -1527.465714
SUM OF ELECTRONIC AND THERMAL FREE ENERGIES: -1527.556968

==> AQ/PA/C4PP/RAF/C6/TS <==
52
XYZ-COORDINATES + ENERGIES

O	2.004820	-1.535444	-0.052822
O	5.900474	-1.502245	-1.283180
O	5.331069	-1.850668	1.418864
O	2.346325	0.695267	-0.642475
O	3.387650	-1.670960	-2.949327
O	3.378487	-0.969990	3.264364
O	0.418943	4.631505	0.984721
O	-7.908099	-2.856169	0.086007
C	4.368716	-1.206909	0.590944
C	4.568631	-1.699807	-0.826501
C	2.951748	-1.587652	1.031554
C	3.651063	-0.932953	-1.762479
C	2.300722	-0.648810	-1.100970
C	2.459325	-0.740103	2.190166
C	1.182850	1.304421	-0.255264
C	-0.054333	0.680948	-0.249933
C	1.356712	2.635195	0.169802
C	-1.186883	1.401499	0.153749
C	0.256088	3.350177	0.552178
C	-1.055657	2.796709	0.442125
C	-2.516726	0.849779	0.235665
C	-2.861302	-0.424735	-0.078157
C	-4.179422	-1.010277	-0.013622
C	-5.335764	-0.311683	0.393956
C	-4.343762	-2.359780	-0.381638
C	-6.568813	-0.921233	0.428699
C	-5.573061	-2.983571	-0.350620
C	-6.747975	-2.289059	0.055656
H	4.313900	-2.762853	-0.866973
H	4.513270	-0.123458	0.633491
H	2.979931	-2.632594	1.341333
H	4.120144	0.020886	-2.007217
H	1.505193	-0.768180	-1.832255
H	1.450937	-1.047576	2.470276
H	2.458719	0.315725	1.918778
H	6.485107	-2.045696	-0.744272
H	5.131231	-1.591928	2.328533
H	4.230925	-1.861299	-3.374560
H	3.205089	-0.329783	3.960303
H	-0.143131	-0.361022	-0.510247
H	2.345914	3.069326	0.197536
H	-3.278189	1.540572	0.577875
H	-2.087105	-1.103538	-0.420473
H	-0.439225	5.028903	1.176942
H	-5.261993	0.728339	0.686035
H	-3.470691	-2.919223	-0.698656
H	-7.443640	-0.365077	0.743189
H	-5.668337	-4.022992	-0.640068
H	-1.854905	3.287135	0.983333
O	-1.635478	3.539356	-1.234864
O	-1.753562	4.926748	-1.082063
H	-0.891119	5.272168	-1.348560

ENERGIES
SCF ENERGY: -1527.89193587
SUM OF ELECTRONIC AND ZERO-POINT ENERGIES: -1527.483523
SUM OF ELECTRONIC AND THERMAL ENERGIES: -1527.454454
SUM OF ELECTRONIC AND THERMAL ENTHALPIES: -1527.453510
SUM OF ELECTRONIC AND THERMAL FREE ENERGIES: -1527.546332

==> AQ/PA/C4PP/RAF/C6PP <==
52
XYZ-COORDINATES + ENERGIES

O	2.033734	-1.191786	-0.039176
O	5.898907	-1.818045	-1.194108
O	5.277949	-1.786041	1.518978
O	2.663653	0.897057	-0.866090
O	3.407051	-1.810698	-2.886993
O	3.457645	-0.434724	3.219072
O	1.226404	5.198803	0.290060
O	-7.993971	-1.100917	-0.537881
C	4.417020	-1.110759	0.608532
C	4.550797	-1.777841	-0.744014
C	2.959621	-1.241817	1.062215
C	3.752726	-0.996868	-1.772645
C	2.446405	-0.469803	-1.172781
C	2.585367	-0.205192	2.105773
C	1.581030	1.688311	-0.552510
C	0.275229	1.228194	-0.499598
C	1.901909	3.017556	-0.284067
C	-0.753407	2.133009	-0.181232
C	0.879294	3.897342	0.031444
C	-0.442174	3.472007	0.080501
C	-2.150763	1.738807	-0.103310
C	-2.634816	0.466772	-0.254683
C	-3.994409	0.084913	-0.189946
C	-5.074338	0.978660	0.011123
C	-4.295572	-1.385243	-0.239241
C	-6.364544	0.544163	-0.029446
C	-5.704779	-1.745984	-0.516257
C	-6.729191	-0.845085	-0.364921
H	4.150341	-2.793450	-0.675348
H	4.710337	-0.059339	0.536206
H	2.843652	-2.237006	1.492342
H	4.347303	-0.143094	-2.100198
H	1.645195	-0.572242	-1.900688
H	1.542371	-0.334505	2.397946
H	2.734763	0.801627	1.715976
H	6.394911	-2.391380	-0.600189
H	5.106147	-1.409264	2.392338
H	4.222467	-2.147217	-3.274702
H	3.401771	0.318652	3.813584
H	0.046924	0.192930	-0.690465
H	2.928992	3.351258	-0.327203
H	-1.226460	4.176650	0.328158
H	-2.839643	2.548099	0.102008
H	-1.935927	-0.341498	-0.439033
H	0.438153	5.703798	0.519914

H	-4.876143	2.029758	0.173510
H	-3.598204	-1.903052	-0.898956
H	-7.180822	1.238025	0.126009
H	-5.928492	-2.762511	-0.815424
O	-3.876400	-1.815004	1.136593
O	-3.995997	-3.229871	1.216184
H	-4.951872	-3.363270	1.301490

ENERGIES

SCF ENERGY: -1527.90093098
SUM OF ELECTRONIC AND ZERO-POINT ENERGIES: -1527.491492
SUM OF ELECTRONIC AND THERMAL ENERGIES: -1527.462286
SUM OF ELECTRONIC AND THERMAL ENTHALPIES: -1527.461342
SUM OF ELECTRONIC AND THERMAL FREE ENERGIES: -1527.554315

==> AQ/PA/C5 <==
49
XYZ-COORDINATES + ENERGIES

O	1.850015	-1.240951	0.163337
O	5.724030	-1.774715	-0.979304
O	5.139259	-1.435240	1.717272
O	2.319933	0.769548	-0.926312
O	3.229507	-2.103959	-2.620799
O	3.257998	0.036512	3.262374
O	0.547111	5.073761	-0.214474
O	-8.192812	-1.902504	0.357384
C	4.227923	-0.926645	0.748565
C	4.380791	-1.748970	-0.513181
C	2.786168	-1.079488	1.243307
C	3.528437	-1.143952	-1.613771
C	2.195720	-0.631348	-1.059104
C	2.362077	0.066159	2.143640
C	1.180144	1.524681	-0.685806
C	-0.087482	0.973406	-0.565726
C	1.414940	2.889895	-0.570374
C	-1.162839	1.849579	-0.327013
C	0.353101	3.790272	-0.329037
C	-0.943568	3.217675	-0.214296
C	-2.544927	1.367409	-0.174349
C	-2.946762	0.090529	-0.227113
C	-4.323626	-0.393299	-0.070138
C	-5.421070	0.446441	0.168302
C	-4.562613	-1.768097	-0.160549
C	-6.699027	-0.066459	0.308881
C	-5.839405	-2.296006	-0.021780
C	-6.907814	-1.440365	0.212841
H	4.036905	-2.767842	-0.312301
H	4.460406	0.121778	0.540475
H	2.737007	-2.010397	1.809122
H	4.066629	-0.300143	-2.047359
H	1.394754	-0.881836	-1.750755
H	1.331431	-0.080942	2.469407
H	2.448985	1.018186	1.620797
H	6.260478	-2.222472	-0.316400
H	4.958840	-0.963509	2.541164
H	4.063793	-2.429850	-2.975844
H	3.164705	0.857629	3.753766
H	-0.246042	-0.089973	-0.629524
H	2.425735	3.264286	-0.671252
H	-1.780478	3.880180	-0.026107
H	-3.274625	2.150848	-0.001158
H	-2.218147	-0.694089	-0.399381
H	-8.204993	-2.861602	0.258963
H	-5.282417	1.516017	0.246071
H	-3.731054	-2.437011	-0.343451
H	-7.542394	0.585354	0.492754
H	-6.010838	-3.362729	-0.094614

ENERGIES

SCF ENERGY: -1376.96773017
SUM OF ELECTRONIC AND ZERO-POINT ENERGIES: -1376.575103
SUM OF ELECTRONIC AND THERMAL ENERGIES: -1376.548994
SUM OF ELECTRONIC AND THERMAL ENTHALPIES: -1376.548050
SUM OF ELECTRONIC AND THERMAL FREE ENERGIES: -1376.633528

==> AQ/RAF/C1 <==
53
XYZ-COORDINATES + ENERGIES

O	2.335921	-0.862346	1.143243
O	5.222815	-2.835038	-0.959905
O	5.948848	-0.698605	0.666829
O	1.881588	-0.222636	-1.054011
O	2.344366	-3.612039	-0.533786
O	4.821304	1.765196	1.503143
O	0.030200	3.685536	-2.839372
O	-7.977926	-2.269988	0.201198
C	4.611096	-0.771532	0.185693
C	4.287174	-2.226559	-0.079329
C	3.636727	-0.254006	1.248253
C	2.919250	-2.326119	-0.730414
C	1.951014	-1.301071	-0.135001
C	3.509968	1.258179	1.227608
C	0.893558	0.718680	-0.879627
C	-0.018615	0.706737	0.120425
C	0.914324	1.730749	-1.873906
C	-1.053223	1.777225	0.225811
C	-0.045759	2.758946	-1.827251
C	-0.985822	2.814078	-0.847444
C	-2.465865	1.252374	0.306508
C	-2.808344	-0.019477	0.091799
C	-4.170039	-0.571295	0.120072
C	-5.315897	0.201146	0.352380
C	-4.335420	-1.942313	-0.091541
C	-6.573573	-0.375741	0.375718
C	-5.591125	-2.534546	-0.070280
C	-6.710384	-1.746409	0.165450
H	4.269798	-2.762096	0.874400
H	4.521691	-0.195242	-0.739848
H	4.023053	-0.564465	2.219492
H	3.024551	-2.119124	-1.796235
H	0.968634	-1.752149	-0.014167
H	2.800981	1.580879	1.991359
H	3.175847	1.601708	0.248664
H	6.082072	-2.841199	-0.524605
H	6.099857	0.220715	0.924955
H	2.944316	-4.265589	-0.909605
H	4.840095	2.702025	1.288194
H	-0.019930	-0.039590	0.899332
H	1.656098	1.703210	-2.657758
H	-1.729288	3.600743	-0.840537
H	-3.210883	2.011293	0.512154
H	-2.032874	-0.744867	-0.131189
H	-0.661503	4.347677	-2.724800
H	-5.232124	1.266574	0.517655
H	-3.463362	-2.557031	-0.275368
H	-7.456585	0.222175	0.556261
H	-5.705894	-3.598510	-0.235258
H	-7.942533	-3.223361	0.061889
O	-0.703767	2.393071	1.532560
O	-1.614493	3.442481	1.833306
H	-1.157134	4.226899	1.499844

ENERGIES

SCF ENERGY: -1528.35813434
SUM OF ELECTRONIC AND ZERO-POINT ENERGIES: -1527.936146
SUM OF ELECTRONIC AND THERMAL ENERGIES: -1527.906825
SUM OF ELECTRONIC AND THERMAL ENTHALPIES: -1527.905880
SUM OF ELECTRONIC AND THERMAL FREE ENERGIES: -1527.998480

==> AQ/RAF/C1P <==
53
XYZ-COORDINATES + ENERGIES

O	2.079384	-1.116828	-0.692381
O	6.156230	-1.052344	-0.923462
O	4.935762	-2.443577	1.149330
O	2.595308	1.093096	-0.147301
O	4.119737	-0.180102	-2.984668
O	2.602260	-2.191984	2.739526
O	0.349797	4.200541	2.536661
O	-7.781935	-2.320982	-0.084683

C	4.217501	-1.391437	0.513974
C	4.748767	-1.253178	-0.897143
C	2.728583	-1.741149	0.430762
C	4.102939	-0.053331	-1.567994
C	2.638051	0.089654	-1.148459
C	1.984353	-1.409187	1.710959
C	1.371976	1.605516	0.218097
C	0.158945	1.169994	-0.300800
C	1.427176	2.615522	1.173865
C	-1.013262	1.778942	0.149218
C	0.248674	3.203227	1.601840
C	-0.982829	2.796095	1.090297
C	-2.339604	1.296108	-0.385020
C	-2.662866	-0.083605	0.070577
C	-3.963707	-0.629630	0.030210
C	-5.087761	0.083417	-0.460124
C	-4.181970	-1.953060	0.489608
C	-6.340803	-0.491756	-0.487400
C	-5.434438	-2.529125	0.461612
C	-6.518760	-1.798093	-0.028693
H	4.490844	-2.156905	-1.457291
H	4.369390	-0.458042	1.063690
H	2.658532	-2.813542	0.246485
H	4.636699	0.847697	-1.263250
H	2.042493	0.389500	-2.007301
H	0.930647	-1.671754	1.604067
H	2.075690	-0.348133	1.943159
H	6.574499	-1.834094	-0.546902
H	4.520260	-2.581445	2.011091
H	5.036562	-0.274639	-3.264363
H	2.296000	-1.875397	3.594183
H	0.097167	0.370196	-1.022963
H	2.380127	2.941501	1.566674
H	-1.896770	3.268915	1.428292
H	-3.122656	2.005835	-0.115144
H	-1.849580	-0.704326	0.420581
H	-0.524401	4.550739	2.742910
H	-4.968105	1.091682	-0.831157
H	-3.340376	-2.518773	0.867597
H	-7.194800	0.053715	-0.865539
H	-5.588136	-3.541581	0.813717
H	-7.781392	-3.227053	0.245511
O	-2.310342	1.222860	-1.836867
O	-2.153256	2.541740	-2.349349
H	-1.192064	2.637013	-2.420798

ENERGIES
SCF ENERGY: -1528.39777670
SUM OF ELECTRONIC AND ZERO-POINT ENERGIES: -1527.973931
SUM OF ELECTRONIC AND THERMAL ENERGIES: -1527.944936
SUM OF ELECTRONIC AND THERMAL ENTHALPIES: -1527.943991
SUM OF ELECTRONIC AND THERMAL FREE ENERGIES: -1528.036021

==> AQ/RAF/C1P/TS <==
53
XYZ-COORDINATES + ENERGIES

O	-1.988173	-1.300200	0.220609
O	-5.926667	-1.447392	1.274106
O	-5.195271	-2.076513	-1.331152
O	-2.494129	0.958955	0.511504
O	-3.526067	-1.136170	3.057105
O	-3.225220	-1.257303	-3.196961
O	-0.642197	4.764534	-1.493499
O	7.757590	-2.483512	-0.136769
C	-4.331687	-1.250155	-0.558483
C	-4.560018	-1.572850	0.902707
C	-2.868160	-1.556576	-0.889664
C	-3.764299	-0.605018	1.759207
C	-2.397707	-0.312523	1.133141
C	-2.388659	-0.812295	-2.122081
C	-1.337742	1.572060	0.085261
C	-0.072235	1.018034	0.217434
C	-1.526677	2.822499	-0.498259
C	1.030755	1.745417	-0.246573
C	-0.421013	3.532511	-0.935465
C	0.862195	3.009012	-0.804825
C	2.395740	1.216729	-0.123339
C	2.676984	-0.130002	-0.206674
C	3.995797	-0.706180	-0.186713
C	5.171838	0.055880	-0.041621
C	4.123172	-2.101201	-0.306604
C	6.411900	-0.549282	-0.027450
C	5.361512	-2.715531	-0.295117
C	6.508165	-1.936038	-0.156882
H	-4.213373	-2.592296	1.095925
H	-4.562830	-0.197766	-0.748412
H	-2.796188	-2.629033	-1.072899
H	-4.316743	0.332745	1.831155
H	-1.640097	-0.293756	1.912862
H	-1.343372	-1.055078	-2.318427
H	-2.496134	0.263699	-1.986014
H	-6.429401	-2.104083	0.780201
H	-4.959786	-1.931170	-2.257360
H	-4.379443	-1.320058	3.464731
H	-3.111798	-0.662681	-3.943975
H	0.074164	0.051889	0.673225
H	-2.521736	3.234309	-0.592604
H	1.717713	3.576639	-1.149018
H	3.183476	1.934143	-0.308013
H	1.854501	-0.833872	-0.245949
H	0.197582	5.160954	-1.753419
H	5.115089	1.129819	0.067612
H	3.229546	-2.702715	-0.411287
H	7.315931	0.033072	0.085864
H	5.451290	-3.790102	-0.390410
H	7.701935	-3.442159	-0.229023
O	2.677612	1.448928	1.921934
O	2.126342	2.657349	2.276538
H	1.199952	2.461123	2.479914

ENERGIES
SCF ENERGY: -1528.36745927
SUM OF ELECTRONIC AND ZERO-POINT ENERGIES: -1527.945778
SUM OF ELECTRONIC AND THERMAL ENERGIES: -1527.916425
SUM OF ELECTRONIC AND THERMAL ENTHALPIES: -1527.915481
SUM OF ELECTRONIC AND THERMAL FREE ENERGIES: -1528.008570

==> AQ/RAF/C1PP <==
53
XYZ-COORDINATES + ENERGIES

O	2.300043	-1.211529	0.130760
O	6.097292	-1.466805	-1.344569
O	5.718648	-1.232118	1.401647
O	2.559452	0.843604	-0.942493
O	3.492403	-1.933881	-2.778598
O	3.891106	0.066355	3.139819
O	0.580577	5.025625	-0.225971
O	-7.646629	-0.445151	-1.471001
C	4.698209	-0.766532	0.525463
C	4.800320	-1.548072	-0.767382
C	3.316673	-1.028105	1.133805
C	3.819252	-0.978640	-1.776363
C	2.509949	-0.564453	-1.099628
C	2.899638	0.060274	2.105689
C	1.400789	1.514829	-0.622888
C	0.185982	0.890330	-0.381295
C	1.535280	2.899751	-0.562249
C	-0.932895	1.680901	-0.087898
C	0.419501	3.664725	-0.269882
C	-0.815959	3.067978	-0.036388
C	-2.259381	1.101830	0.181204
C	-2.537435	-0.195851	0.304432
C	-3.923637	-0.745977	0.528037
C	-4.962434	0.274701	0.846836
C	-4.292778	-1.626904	-0.621206
C	-6.149389	0.331087	0.195828
C	-5.489917	-1.533831	-1.257371
C	-6.435735	-0.565704	-0.860027
H	4.545522	-2.593034	-0.568235
H	4.841298	0.299911	0.327726

H	3.372618	-1.978124	1.665887
H	4.263630	-0.092980	-2.232033
H	1.670902	-0.844143	-1.732242
H	1.914386	-0.167045	2.515038
H	2.875061	1.029060	1.607054
H	6.718362	-1.893818	-0.744972
H	5.580347	-0.787332	2.248416
H	4.307045	-2.183230	-3.228783
H	3.788603	0.868485	3.659962
H	0.099021	-0.183224	-0.412358
H	2.492120	3.364407	-0.754659
H	-1.678569	3.682736	0.189153
H	-3.059494	1.827093	0.280088
H	-1.773009	-0.958514	0.203733
H	-0.265819	5.447563	-0.038159
H	-4.746141	0.954896	1.659414
H	-3.557283	-2.359802	-0.926120
H	-6.898995	1.062798	0.467669
H	-5.729218	-2.193027	-2.083311
H	-7.738367	-1.108660	-2.165802
O	-3.698838	-1.594131	1.731136
O	-4.901830	-2.267627	2.068424
H	-4.876020	-3.062765	1.516994

ENERGIES
SCF ENERGY: -1528.36398858
SUM OF ELECTRONIC AND ZERO-POINT ENERGIES: -1527.941399
SUM OF ELECTRONIC AND THERMAL ENERGIES: -1527.912095
SUM OF ELECTRONIC AND THERMAL ENTHALPIES: -1527.911151
SUM OF ELECTRONIC AND THERMAL FREE ENERGIES: -1528.003224

==> AQ/RAF/C2 <==
53
XYZ-COORDINATES + ENERGIES

O	1.986114	-1.121987	0.702971
O	5.531176	-2.307072	-0.948552
O	5.506988	-1.292447	1.641249
O	2.217486	0.484987	-0.970975
O	2.812437	-2.701597	-2.067466
O	4.057337	0.658202	3.101109
O	0.730759	4.829656	-0.015527
O	-8.245166	-1.926849	0.830576
C	4.451710	-0.938844	0.755061
C	4.308744	-2.038785	-0.274127
C	3.132311	-0.823020	1.522732
C	3.306883	-1.595887	-1.322033
C	2.100273	-0.905445	-0.679301
C	2.971597	0.537229	2.173728
C	1.170108	1.309192	-0.632914
C	-0.219127	0.754267	-0.636027
C	1.432930	2.617684	-0.448891
C	-1.256514	1.736756	-0.176340
C	0.369710	3.521717	-0.165585
C	-0.932677	3.080121	-0.016274
C	-2.593311	1.288427	0.055908
C	-3.029657	0.007407	-0.055300
C	-4.389908	-0.458546	0.186572
C	-5.456773	0.389169	0.527435
C	-4.659590	-1.828541	0.067041
C	-6.729354	-0.110343	0.739679
C	-5.930902	-2.340908	0.278405
C	-6.966683	-1.477649	0.614720
H	3.945575	-2.945583	0.217983
H	4.691548	0.004835	0.254367
H	3.138543	-1.590326	2.297434
H	3.796623	-0.883239	-1.987275
H	1.191372	-1.308234	-1.114974
H	2.012242	0.592034	2.690233
H	3.030668	1.325449	1.424234
H	6.149560	-2.679831	-0.311296
H	5.513704	-0.626784	2.342312
H	3.562620	-3.128396	-2.495580
H	4.129373	1.577520	3.373416
H	2.448560	2.986184	-0.502675
H	-1.705961	3.787926	0.259551
H	-3.290995	2.064582	0.350501
H	-2.334072	-0.769124	-0.350071
H	-0.044312	5.364318	0.194530
H	-5.297474	1.454096	0.624980
H	-3.854054	-2.502242	-0.196420
H	-7.548272	0.546877	0.999240
H	-6.125330	-3.401765	0.182238
H	-8.277509	-2.882110	0.703155
H	-0.280762	-0.165275	-0.050849
O	-0.523589	0.242193	-1.974260
O	-0.535439	1.324135	-2.892137
H	0.364923	1.321316	-3.247866

ENERGIES
SCF ENERGY: -1528.37846066
SUM OF ELECTRONIC AND ZERO-POINT ENERGIES: -1527.955535
SUM OF ELECTRONIC AND THERMAL ENERGIES: -1527.926147
SUM OF ELECTRONIC AND THERMAL ENTHALPIES: -1527.925203
SUM OF ELECTRONIC AND THERMAL FREE ENERGIES: -1528.018101

==> AQ/RAF/C2/TS <==
53
XYZ-COORDINATES + ENERGIES

O	1.849727	-1.321236	0.349195
O	5.623650	-2.019126	-1.035764
O	5.216968	-1.703878	1.693813
O	2.301930	0.679261	-0.757744
O	3.061442	-2.150582	-2.564602
O	3.497336	-0.195500	3.386843
O	0.688109	4.880405	0.504711
O	-8.109000	-2.132263	0.501510
C	4.273642	-1.137926	0.791577
C	4.312730	-1.938664	-0.492621
C	2.860027	-1.233723	1.372553
C	3.436185	-1.255526	-1.525290
C	2.145962	-0.730613	-0.889093
C	2.548680	-0.089402	2.318935
C	1.218573	1.432472	-0.394796
C	-0.105459	0.905691	-0.391040
C	1.464233	2.757692	-0.133136
C	-1.155075	1.741712	0.113656
C	0.397813	3.579190	0.248573
C	-0.896517	3.075120	0.384692
C	-2.507883	1.236654	0.285937
C	-2.928109	0.004473	-0.050351
C	-4.285393	-0.517617	0.107364
C	-5.348890	0.227553	0.638496
C	-4.542745	-1.832970	-0.293961
C	-6.612472	-0.320632	0.763146
C	-5.805524	-2.394869	-0.173601
C	-6.840410	-1.634365	0.356903
H	3.926583	-2.943283	-0.297523
H	4.539314	-0.096272	0.586313
H	2.796591	-2.174606	1.919545
H	3.984445	-0.407840	-1.938489
H	1.305814	-0.957528	-1.539092
H	1.528734	-0.186542	2.693953
H	2.663870	0.868477	1.811980
H	6.170042	-2.521963	-0.422303
H	5.113403	-1.236511	2.533259
H	3.865161	-2.447449	-3.005435
H	3.466563	0.612562	3.906913
H	2.467474	3.155344	-0.194482
H	-1.691331	3.728017	0.723605
H	-3.206013	1.949005	0.709801
H	-2.229575	-0.698604	-0.489627
H	-0.106694	5.349209	0.787732
H	-5.194674	1.248797	0.958502
H	-3.736184	-2.425194	-0.707327
H	-7.431158	0.255689	1.172194
H	-5.992862	-3.414290	-0.487014
H	-8.139845	-3.043252	0.187090
H	-0.234328	-0.162269	-0.319769
O	-0.452655	0.835863	-2.269789
O	-0.443151	2.127916	-2.784762

H	0.483603	2.296129	-3.004776

\# ENERGIES
SCF ENERGY: -1528.36388488
SUM OF ELECTRONIC AND ZERO-POINT ENERGIES: -1527.942026
SUM OF ELECTRONIC AND THERMAL ENERGIES: -1527.912632
SUM OF ELECTRONIC AND THERMAL ENTHALPIES: -1527.911688
SUM OF ELECTRONIC AND THERMAL FREE ENERGIES: -1528.004820

==> AQ/RAF/C2P <==
53
XYZ-COORDINATES + ENERGIES

O	2.050225	-1.239635	0.054882
O	5.967561	-1.659628	-1.001351
O	5.307588	-1.390413	1.686115
O	2.514339	0.797856	-0.988934
O	3.511452	-2.037175	-2.702534
O	3.345478	-0.033152	3.228174
O	0.675695	5.076673	-0.490777
O	-8.108697	-1.551183	-0.140375
C	4.406499	-0.888983	0.704890
C	4.613755	-1.683057	-0.567499
C	2.958577	-1.091938	1.161334
C	3.770612	-1.084202	-1.678874
C	2.416514	-0.607823	-1.146614
C	2.483142	0.014630	2.084605
C	1.362175	1.520999	-0.774275
C	0.114937	0.950297	-0.599858
C	1.550008	2.902796	-0.731149
C	-1.001877	1.792923	-0.380872
C	0.447916	3.723209	-0.519481
C	-0.817464	3.192006	-0.346008
C	-2.303831	1.272632	-0.178673
C	-2.609951	-0.184958	-0.160497
C	-4.079609	-0.512828	-0.170103
C	-5.003812	0.183715	0.612138
C	-4.531777	-1.578508	-0.946499
C	-6.344191	-0.173414	0.617817
C	-5.871017	-1.943792	-0.955159
C	-6.773941	-1.236612	-0.169247
H	4.295962	-2.715164	-0.392793
H	4.613879	0.169385	0.521879
H	2.918696	-2.041130	1.696517
H	4.298272	-0.223156	-2.091068
H	1.634385	-0.852101	-1.861924
H	1.445972	-0.163954	2.372829
H	2.566024	0.984896	1.594904
H	6.502718	-2.099118	-0.331857
H	5.088956	-0.945663	2.515876
H	4.359008	-2.343312	-3.043610
H	3.232890	0.776329	3.734571
H	-0.012228	-0.118947	-0.602122
H	2.535933	3.323997	-0.867059
H	-1.663329	3.846127	-0.175913
H	-3.107483	1.977342	-0.025492
H	-0.158165	5.541967	-0.357336
H	-4.682540	1.015269	1.224811
H	-3.827440	-2.128384	-1.558028
H	-7.062399	0.364330	1.221595
H	-6.219993	-2.767416	-1.565254
H	-8.280625	-2.288484	-0.737195
H	-2.133078	-0.687611	-1.005068
O	-1.933703	-0.857693	0.948873
O	-2.328449	-0.258756	2.173367
H	-3.077323	-0.802915	2.456336

\# ENERGIES
SCF ENERGY: -1528.39447621
SUM OF ELECTRONIC AND ZERO-POINT ENERGIES: -1527.970860
SUM OF ELECTRONIC AND THERMAL ENERGIES: -1527.941746
SUM OF ELECTRONIC AND THERMAL ENTHALPIES: -1527.940802
SUM OF ELECTRONIC AND THERMAL FREE ENERGIES: -1528.033110

==> AQ/RAF/C2P/TS <==
53
XYZ-COORDINATES + ENERGIES

O	1.990010	-1.236976	-0.069805
O	5.934044	-1.724850	-0.991283
O	5.163720	-1.645728	1.681342
O	2.579064	0.861027	-0.912201
O	3.538729	-1.866874	-2.815680
O	3.177953	-0.354744	3.248362
O	0.859033	5.152157	-0.163835
O	-7.923689	-1.815298	-0.202658
C	4.325284	-1.033883	0.707558
C	4.562504	-1.728818	-0.616877
C	2.851894	-1.221942	1.083130
C	3.791321	-1.009245	-1.709325
C	2.434590	-0.521735	-1.195174
C	2.368745	-0.183762	2.078704
C	1.446072	1.609634	-0.692154
C	0.171411	1.073168	-0.610879
C	1.674854	2.975801	-0.539547
C	-0.913062	1.935485	-0.372277
C	0.595942	3.814438	-0.304916
C	-0.694351	3.308783	-0.215761
C	-2.270169	1.448521	-0.243345
C	-2.652791	0.131273	-0.415740
C	-4.041716	-0.327638	-0.386441
C	-5.090739	0.428992	0.151573
C	-4.324252	-1.615758	-0.853010
C	-6.378782	-0.076062	0.201428
C	-5.609328	-2.132399	-0.809055
C	-6.636360	-1.357490	-0.278873
H	4.200388	-2.758307	-0.541713
H	4.574534	0.027824	0.622185
H	2.756323	-2.212483	1.528790
H	4.368431	-0.140902	-2.029759
H	1.676545	-0.669065	-1.961198
H	1.315434	-0.354024	2.307058
H	2.498958	0.821485	1.677843
H	6.418046	-2.259992	-0.353386
H	4.932670	-1.251815	2.533112
H	4.386518	-2.184569	-3.145473
H	3.032141	0.392507	3.835499
H	0.014091	0.010681	-0.691013
H	2.678802	3.370810	-0.606303
H	-1.525915	3.974340	-0.021272
H	-3.003075	2.182324	0.064130
H	0.035404	5.634604	-0.026612
H	-4.909144	1.420457	0.542157
H	-3.521047	-2.218759	-1.257306
H	-7.189614	0.506692	0.616440
H	-5.821909	-3.128623	-1.176011
H	-7.974800	-2.713881	-0.549101
H	-1.968611	-0.547521	-0.906057
O	-1.973969	-0.597960	1.369204
O	-2.974664	-0.496577	2.304728
H	-3.602456	-1.199387	2.078486

\# ENERGIES
SCF ENERGY: -1528.36705919
SUM OF ELECTRONIC AND ZERO-POINT ENERGIES: -1527.945559
SUM OF ELECTRONIC AND THERMAL ENERGIES: -1527.916176
SUM OF ELECTRONIC AND THERMAL ENTHALPIES: -1527.915232
SUM OF ELECTRONIC AND THERMAL FREE ENERGIES: -1528.008445

==> AQ/RAF/C2PP <==
53
XYZ-COORDINATES + ENERGIES

O	2.332970	-1.267792	0.196075
O	6.293597	-1.600746	-0.732848
O	5.549784	-1.172925	1.913981
O	2.752368	0.707743	-0.974592
O	3.894476	-2.205371	-2.455392
O	3.499688	0.212336	3.304367
O	0.761196	4.936818	-0.713230
O	-7.653101	-2.428808	-0.166867
C	4.657109	-0.780101	0.877116
C	4.930859	-1.651759	-0.329971

C	3.206474	-1.010746	1.311505
C	4.093673	-1.169304	-1.501468
C	2.709760	-0.708025	-1.037424
C	2.663261	0.136136	2.143410
C	1.571511	1.402350	-0.837369
C	0.330141	0.800453	-0.705929
C	1.717356	2.788270	-0.835531
C	-0.809036	1.613532	-0.580871
C	0.585632	3.576963	-0.709174
C	-0.674523	3.004549	-0.584530
C	-2.152204	1.067490	-0.433765
C	-2.472355	-0.255522	-0.383423
C	-3.786896	-0.773246	-0.245091
C	-4.960935	0.148216	-0.070204
C	-3.991468	-2.159353	-0.223870
C	-6.294179	-0.512620	-0.154086
C	-5.248895	-2.708582	-0.156036
C	-6.404945	-1.853222	-0.150515
H	4.651201	-2.681661	-0.089574
H	4.826837	0.270608	0.624876
H	3.189312	-1.922710	1.908868
H	4.599094	-0.321781	-1.966019
H	1.959176	-1.031751	-1.754628
H	1.629251	-0.068872	2.424059
H	2.713275	1.070986	1.585295
H	6.829713	-1.966307	-0.021006
H	5.301077	-0.666937	2.698988
H	4.760695	-2.502319	-2.754983
H	3.319101	1.039022	3.761368
H	0.236947	-0.272573	-0.684264
H	2.696808	3.234171	-0.936355
H	-1.547483	3.637593	-0.485310
H	-2.938957	1.807100	-0.353744
H	-1.688926	-1.000739	-0.458685
H	-0.092532	5.375258	-0.620101
H	-3.127433	-2.808548	-0.283595
H	-7.178451	0.109263	-0.179376
H	-5.389225	-3.781092	-0.139512
H	-7.573447	-3.387610	-0.221154
H	-4.903348	0.992067	-0.759171
O	-4.759120	0.721831	1.269442
O	-5.655344	1.813602	1.421932
H	-6.426821	1.410442	1.844411

\# ENERGIES
SCF ENERGY: -1528.37461837
SUM OF ELECTRONIC AND ZERO-POINT ENERGIES: -1527.951923
SUM OF ELECTRONIC AND THERMAL ENERGIES: -1527.922536
SUM OF ELECTRONIC AND THERMAL ENTHALPIES: -1527.921592
SUM OF ELECTRONIC AND THERMAL FREE ENERGIES: -1528.014869

==> AQ/RAF/C2PP/TS <==
53
XYZ-COORDINATES + ENERGIES

O	2.322098	-1.271304	0.158141
O	6.303888	-1.596375	-0.683515
O	5.498005	-1.220351	1.953791
O	2.775845	0.725776	-0.961388
O	3.944539	-2.159169	-2.474819
O	3.417811	0.138282	3.324619
O	0.795348	4.953689	-0.623391
O	-7.652414	-2.374375	-0.150025
C	4.631044	-0.805284	0.903926
C	4.931977	-1.653012	-0.313729
C	3.170064	-1.041454	1.299048
C	4.123752	-1.144264	-1.494576
C	2.730436	-0.688353	-1.054293
C	2.609374	0.088311	2.142609
C	1.594597	1.421305	-0.835433
C	0.346959	0.821887	-0.759372
C	1.745520	2.805552	-0.785454
C	-0.789639	1.636834	-0.642173
C	0.614484	3.595551	-0.668517
C	-0.651769	3.024800	-0.600358
C	-2.144948	1.092025	-0.550335
C	-2.468196	-0.216420	-0.502231
C	-3.809201	-0.740669	-0.398859
C	-4.968930	0.103080	-0.352814
C	-3.992930	-2.119880	-0.254151
C	-6.260839	-0.483472	-0.351458
C	-5.254725	-2.670958	-0.146475
C	-6.393228	-1.840826	-0.207616
H	4.645007	-2.687082	-0.101395
H	4.808273	0.249924	0.676830
H	3.136853	-1.966255	1.875656
H	4.642276	-0.288671	-1.928967
H	1.996493	-0.994569	-1.795826
H	1.568599	-0.120423	2.393968
H	2.674289	1.034838	1.606118
H	6.823228	-1.973655	0.034572
H	5.230358	-0.730669	2.742928
H	4.816486	-2.447702	-2.765847
H	3.227195	0.955532	3.794415
H	0.247087	-0.250760	-0.777220
H	2.729515	3.249270	-0.842535
H	-1.525314	3.658047	-0.508063
H	-2.928814	1.838366	-0.506867
H	-1.687104	-0.967559	-0.524480
H	-0.058466	5.393477	-0.537596
H	-3.125650	-2.766724	-0.237635
H	-7.140045	0.142090	-0.416164
H	-5.384214	-3.739440	-0.030591
H	-7.602492	-3.335785	-0.092987
H	-4.889668	1.117557	-0.713847
O	-4.731174	0.706909	1.472710
O	-5.715912	1.645245	1.732554
H	-6.450972	1.135063	2.099685

\# ENERGIES
SCF ENERGY: -1528.35617588
SUM OF ELECTRONIC AND ZERO-POINT ENERGIES: -1527.935155
SUM OF ELECTRONIC AND THERMAL ENERGIES: -1527.905616
SUM OF ELECTRONIC AND THERMAL ENTHALPIES: -1527.904671
SUM OF ELECTRONIC AND THERMAL FREE ENERGIES: -1527.998692

==> AQ/RAF/C3 <==
53
XYZ-COORDINATES + ENERGIES

O	1.162778	-1.310967	-0.566275
O	4.952777	-2.694121	0.043745
O	3.081567	-2.912043	2.091865
O	2.418474	0.647830	-0.407676
O	3.616049	-1.837419	-2.538353
O	0.857671	-1.459771	3.055696
O	1.383226	4.252407	2.070624
O	-7.941095	-1.546001	-0.324506
C	2.889718	-1.918102	1.090179
C	3.580623	-2.388385	-0.172691
C	1.397340	-1.758496	0.780630
C	3.514702	-1.295587	-1.225813
C	2.186962	-0.535611	-1.148665
C	0.694589	-0.845967	1.769639
C	1.576676	1.768066	-0.691720
C	0.122747	1.428166	-0.750428
C	1.934577	2.804464	0.314002
C	-0.782683	1.947062	0.135956
C	0.997246	3.315566	1.142211
C	-0.362098	2.904947	1.085425
C	-2.196205	1.541537	0.147503
C	-2.640086	0.367535	-0.319107
C	-4.031368	-0.095830	-0.324358
C	-5.114896	0.707251	0.057780
C	-4.296897	-1.408324	-0.725065
C	-6.407191	0.213133	0.052309
C	-5.588805	-1.916500	-0.736298
C	-6.643081	-1.102512	-0.341753
H	3.057796	-3.273898	-0.545851
H	3.323548	-0.971697	1.425054
H	0.951188	-2.752177	0.838565
H	4.325087	-0.587461	-1.048386

H	1.845997	-0.300776	-2.153903
H	-0.363006	-0.767320	1.512855
H	1.148412	0.144839	1.765536
H	4.998953	-3.458763	0.627469
H	2.547570	-2.643312	2.851575
H	4.443626	-2.327720	-2.594595
H	0.604269	-0.826915	3.733708
H	-0.184414	0.760610	-1.542142
H	2.970127	3.103319	0.387760
H	-1.076026	3.335583	1.774774
H	-2.878710	2.246744	0.607778
H	-1.923523	-0.343689	-0.718437
H	0.625015	4.530000	2.596769
H	-4.952866	1.732521	0.361265
H	-3.474686	-2.043276	-1.030208
H	-7.241973	0.834037	0.348015
H	-5.782612	-2.935921	-1.045679
H	-7.976926	-2.468350	-0.603108
O	1.850079	2.199469	-2.042267
O	3.220747	2.548896	-2.170406
H	3.203761	3.512911	-2.086816

ENERGIES
SCF ENERGY: -1528.36923151
SUM OF ELECTRONIC AND ZERO-POINT ENERGIES: -1527.947073
SUM OF ELECTRONIC AND THERMAL ENERGIES: -1527.917833
SUM OF ELECTRONIC AND THERMAL ENTHALPIES: -1527.916889
SUM OF ELECTRONIC AND THERMAL FREE ENERGIES: -1528.008645

==> AQ/RAF/C3PP <==
53
XYZ-COORDINATES + ENERGIES

O	2.461682	-1.233472	0.161019
O	6.376733	-1.773049	-0.839513
O	5.689126	-1.466571	1.837113
O	2.956332	0.784279	-0.898839
O	3.965561	-2.046910	-2.592538
O	3.757410	-0.005072	3.327578
O	1.136096	5.049611	-0.247327
O	-7.527870	-2.150799	-0.134298
C	4.818869	-0.940682	0.841176
C	5.017008	-1.746013	-0.425212
C	3.358324	-1.091458	1.278309
C	4.214581	-1.114888	-1.547329
C	2.855199	-0.622450	-1.042624
C	2.906604	0.046083	2.175635
C	1.804366	1.513675	-0.701109
C	0.536698	0.955262	-0.638909
C	2.007867	2.885089	-0.565306
C	-0.567163	1.797798	-0.443795
C	0.907797	3.703162	-0.373875
C	-0.377228	3.172719	-0.313195
C	-1.944423	1.292202	-0.356674
C	-2.315852	0.008367	-0.423551
C	-3.697485	-0.491388	-0.332973
C	-4.798470	0.319697	-0.384901
C	-3.866638	-1.899026	-0.213908
C	-6.181829	-0.198811	-0.235215
C	-5.144592	-2.476781	-0.151342
C	-6.253134	-1.684727	-0.189772
H	4.658614	-2.765418	-0.255328
H	5.063378	0.109594	0.655533
H	3.281070	-2.029117	1.829295
H	4.769174	-0.257759	-1.931668
H	2.087677	-0.874452	-1.770310
H	1.862635	-0.096479	2.458066
H	3.022596	1.003943	1.668888
H	6.881441	-2.252283	-0.173836
H	5.477737	-1.007570	2.660783
H	4.816319	-2.363114	-2.916022
H	3.659244	0.813953	3.821469
H	0.398382	-0.109832	-0.724286
H	3.005906	3.297117	-0.615163
H	-1.224647	3.829134	-0.158933
H	-2.695653	2.061394	-0.216608
H	-1.566898	-0.766573	-0.539511
H	0.297928	5.512458	-0.133299
H	-4.708298	1.387973	-0.519616
H	-2.992281	-2.532641	-0.179897
H	-5.259397	-3.550983	-0.078294
H	-7.533834	-3.112899	-0.041034
H	-6.855989	0.202357	-0.995681
O	-6.681594	0.341245	1.042126
O	-8.101156	0.427845	1.001423
H	-8.386674	-0.482637	1.168926

ENERGIES
SCF ENERGY: -1528.36665796
SUM OF ELECTRONIC AND ZERO-POINT ENERGIES: -1527.943402
SUM OF ELECTRONIC AND THERMAL ENERGIES: -1527.914146
SUM OF ELECTRONIC AND THERMAL ENTHALPIES: -1527.913202
SUM OF ELECTRONIC AND THERMAL FREE ENERGIES: -1528.005871

==> AQ/RAF/C4 <==
53
XYZ-COORDINATES + ENERGIES

O	1.560241	-1.715549	0.078551
O	5.443478	-2.251877	-1.061391
O	4.801451	-2.306137	1.645105
O	2.157791	0.407630	-0.678955
O	2.970943	-2.217907	-2.778901
O	2.946465	-1.029873	3.368373
O	0.715695	4.668397	0.682988
O	-8.513294	-1.686483	-0.001110
C	3.937284	-1.617775	0.748104
C	4.091465	-2.242661	-0.622575
C	2.478573	-1.784050	1.186079
C	3.291853	-1.439489	-1.632762
C	1.970866	-0.956390	-1.029354
C	2.079104	-0.783656	2.255463
C	1.057718	1.155568	-0.361126
C	-0.215065	0.717132	-0.296038
C	1.463629	2.562281	-0.079726
C	-1.281760	1.608439	0.073630
C	0.320358	3.397547	0.376789
C	-0.954079	2.958116	0.415405
C	-2.640930	1.204601	0.131101
C	-3.133836	-0.034999	-0.166239
C	-4.531115	-0.430279	-0.106712
C	-5.573720	0.435402	0.269086
C	-4.871962	-1.748672	-0.445521
C	-6.887105	0.004097	0.300015
C	-6.185273	-2.192014	-0.416400
C	-7.194713	-1.310703	-0.044928
H	3.703176	-3.264710	-0.588500
H	4.214937	-0.560303	0.708696
H	2.374399	-2.793080	1.585690
H	3.874923	-0.564432	-1.922809
H	1.175882	-1.044013	-1.766447
H	1.036163	-0.938190	2.535332
H	2.214715	0.236064	1.894568
H	5.942963	-2.830098	-0.475080
H	4.625400	-1.950496	2.526360
H	3.796072	-2.500204	-3.188533
H	2.868278	-0.297822	3.986580
H	-0.437935	-0.320631	-0.486886
H	2.294029	2.586517	0.630801
H	-1.744603	3.633891	0.720245
H	-3.331959	1.979404	0.443055
H	-2.456045	-0.818670	-0.481702
H	-0.052013	5.210837	0.903669
H	-5.361699	1.460423	0.539122
H	-4.087664	-2.435555	-0.737448
H	-7.685357	0.674978	0.587468
H	-6.432458	-3.212643	-0.680318
H	-8.601134	-2.605877	-0.277239
O	1.987390	3.102427	-1.330732
O	2.967721	4.087789	-1.042799
H	2.439137	4.872318	-0.831395

ENERGIES
SCF ENERGY: -1528.38043385
SUM OF ELECTRONIC AND ZERO-POINT ENERGIES: -1527.957175
SUM OF ELECTRONIC AND THERMAL ENERGIES: -1527.927662
SUM OF ELECTRONIC AND THERMAL ENTHALPIES: -1527.926717
SUM OF ELECTRONIC AND THERMAL FREE ENERGIES: -1528.020632

==> AQ/RAF/C4/TS <==
53
XYZ-COORDINATES + ENERGIES

O	1.617610	-1.695000	0.084288
O	5.480689	-2.183747	-1.142759
O	4.888483	-2.329651	1.570929
O	2.187989	0.455031	-0.615710
O	2.984431	-2.088967	-2.816564
O	3.059894	-1.136176	3.374188
O	0.734825	4.562189	1.052803
O	-8.434940	-1.822742	-0.055145
C	4.006234	-1.614309	0.713756
C	4.136685	-2.190777	-0.680224
C	2.556595	-1.801566	1.171668
C	3.319317	-1.352969	-1.646806
C	2.004941	-0.900092	-1.006059
C	2.175443	-0.843944	2.285817
C	1.093822	1.177101	-0.228916
C	-0.192533	0.708688	-0.191167
C	1.408192	2.528446	0.092966
C	-1.232252	1.564024	0.251712
C	0.369314	3.320215	0.642573
C	-0.930513	2.865218	0.681526
C	-2.620412	1.139523	0.304194
C	-3.101626	-0.028939	-0.162167
C	-4.495282	-0.464197	-0.116248
C	-5.516867	0.265047	0.511674
C	-4.835123	-1.676412	-0.727883
C	-6.819762	-0.197202	0.523281
C	-6.138849	-2.150012	-0.725111
C	-7.130602	-1.406781	-0.096277
H	3.748974	-3.213601	-0.675333
H	4.278951	-0.554780	0.706097
H	2.462206	-2.825206	1.534925
H	3.894613	-0.464933	-1.911782
H	1.200492	-0.972438	-1.733953
H	1.136643	-1.007642	2.575756
H	2.308228	0.189309	1.965217
H	5.991383	-2.778688	-0.583361
H	4.721692	-2.009761	2.467751
H	3.804862	-2.353271	-3.247081
H	3.012161	-0.417671	4.011190
H	-0.413882	-0.313225	-0.451168
H	2.435652	2.783662	0.309238
H	-1.716513	3.509372	1.055263
H	-3.297425	1.859130	0.749203
H	-2.429264	-0.736360	-0.634090
H	-0.039828	5.051861	1.356450
H	-5.296259	1.202866	1.002391
H	-4.061611	-2.255688	-1.215886
H	-7.605554	0.364164	1.010115
H	-6.390929	-3.088446	-1.202843
H	-8.523761	-2.666380	-0.513618
O	1.539432	3.280092	-1.691794
O	2.291048	4.448077	-1.604656
H	1.643156	5.138512	-1.408965

ENERGIES
SCF ENERGY: -1528.36123309
SUM OF ELECTRONIC AND ZERO-POINT ENERGIES: -1527.939342
SUM OF ELECTRONIC AND THERMAL ENERGIES: -1527.909889
SUM OF ELECTRONIC AND THERMAL ENTHALPIES: -1527.908945
SUM OF ELECTRONIC AND THERMAL FREE ENERGIES: -1528.002529

==> AQ/RAF/C4PP <==
53
XYZ-COORDINATES + ENERGIES

O	2.362376	-1.273956	0.065673
O	6.255918	-1.941131	-0.954605
O	5.558712	-1.815645	1.734949
O	3.000098	0.789005	-0.818824
O	3.828186	-1.970930	-2.726162
O	3.689588	-0.392913	3.332859
O	1.471029	5.129535	0.034514
O	-7.391482	-1.207059	-1.127699
C	4.725582	-1.169014	0.779196
C	4.895473	-1.881168	-0.545907
C	3.255240	-1.280179	1.195028
C	4.132541	-1.128787	-1.621005
C	2.804820	-0.590783	-1.080672
C	2.853299	-0.202766	2.185316
C	1.898842	1.580525	-0.581712
C	0.598371	1.104246	-0.539913
C	2.191943	2.928129	-0.382365
C	-0.450969	2.010090	-0.304758
C	1.148582	3.809814	-0.151130
C	-0.167896	3.366421	-0.112790
C	-1.844946	1.598387	-0.241742
C	-2.304100	0.316121	-0.369654
C	-3.657443	-0.091289	-0.308513
C	-4.762404	0.815716	-0.124845
C	-3.962410	-1.487406	-0.453094
C	-6.033488	0.385202	-0.060700
C	-5.220342	-1.960430	-0.384928
C	-6.389306	-1.062907	-0.145312
H	4.487984	-2.892403	-0.456684
H	5.024627	-0.121310	0.679772
H	3.124170	-2.257196	1.660967
H	4.737102	-0.282035	-1.948583
H	2.026744	-0.726316	-1.828107
H	1.801110	-0.317260	2.449485
H	3.019537	0.787816	1.762343
H	6.733713	-2.486037	-0.320270
H	5.366955	-1.408898	2.590402
H	4.658045	-2.311501	-3.078045
H	3.616024	0.380873	3.898618
H	0.391462	0.055856	-0.674004
H	3.214779	3.275577	-0.417989
H	-0.969511	4.071489	0.069436
H	-2.548073	2.403375	-0.072771
H	-1.591897	-0.484776	-0.529116
H	0.667768	5.646290	0.165224
H	-4.569689	1.876664	-0.049532
H	-3.140987	-2.170373	-0.630780
H	-6.855542	1.076984	0.056944
H	-5.443592	-3.013401	-0.503153
H	-7.528908	-2.148603	-1.296810
O	-6.919012	-1.542919	1.117985
O	-8.070486	-0.788695	1.470150
H	-8.769570	-1.196697	0.938932

ENERGIES
SCF ENERGY: -1528.38145125
SUM OF ELECTRONIC AND ZERO-POINT ENERGIES: -1527.958000
SUM OF ELECTRONIC AND THERMAL ENERGIES: -1527.928726
SUM OF ELECTRONIC AND THERMAL ENTHALPIES: -1527.927782
SUM OF ELECTRONIC AND THERMAL FREE ENERGIES: -1528.020504

==> AQ/RAF/C4PP/TS <==
53
XYZ-COORDINATES + ENERGIES

O	2.344696	-1.280919	0.007905
O	6.279134	-1.851750	-0.915792
O	5.500239	-1.823681	1.756564
O	2.987520	0.818640	-0.779842
O	3.895491	-1.874171	-2.752930
O	3.558060	-0.495385	3.343233
O	1.397835	5.103079	0.232259
O	-7.656305	-1.364461	-0.742937
C	4.684171	-1.162310	0.796204
C	4.906710	-1.828461	-0.545077
C	3.204240	-1.314793	1.162915
C	4.160846	-1.057242	-1.619141

C	2.812814	-0.551724	-1.099128
C	2.751721	-0.282210	2.178663
C	1.873349	1.591557	-0.544032
C	0.574668	1.107673	-0.573423
C	2.150022	2.928923	-0.268079
C	-0.484852	1.996773	-0.333938
C	1.094265	3.792780	-0.031826
C	-0.220398	3.340601	-0.064926
C	-1.886548	1.577205	-0.345741
C	-2.334422	0.321054	-0.551468
C	-3.721523	-0.085428	-0.564858
C	-4.803771	0.818546	-0.426705
C	-4.013764	-1.460016	-0.749464
C	-6.094311	0.373070	-0.417120
C	-5.299082	-1.924519	-0.761873
C	-6.369215	-1.026011	-0.482163
H	4.515691	-2.849056	-0.499370
H	4.965537	-0.106701	0.739332
H	3.078015	-2.310939	1.587848
H	4.761473	-0.194068	-1.908953
H	2.058672	-0.666151	-1.874110
H	1.694978	-0.427507	2.407216
H	2.908553	0.725957	1.795282
H	6.747747	-2.413840	-0.289744
H	5.281379	-1.440859	2.616579
H	4.737414	-2.205341	-3.084521
H	3.445331	0.253064	3.936077
H	0.377810	0.066001	-0.765409
H	3.171471	3.281796	-0.247762
H	-1.033254	4.031531	0.121736
H	-2.594131	2.376846	-0.163749
H	-1.625587	-0.481652	-0.716503
H	0.585200	5.603261	0.369680
H	-4.616986	1.878998	-0.339696
H	-3.192928	-2.148178	-0.904006
H	-6.928642	1.053024	-0.315398
H	-5.525847	-2.970972	-0.918750
H	-7.743411	-2.325072	-0.789244
O	-6.358427	-1.384568	1.485289
O	-7.544130	-0.863529	1.982024
H	-8.210888	-1.528997	1.764361

ENERGIES
SCF ENERGY: -1528.35786409
SUM OF ELECTRONIC AND ZERO-POINT ENERGIES: -1527.936873
SUM OF ELECTRONIC AND THERMAL ENERGIES: -1527.907343
SUM OF ELECTRONIC AND THERMAL ENTHALPIES: -1527.906399
SUM OF ELECTRONIC AND THERMAL FREE ENERGIES: -1528.000333

==> AQ/RAF/C5 <==
53
XYZ-COORDINATES + ENERGIES

O	1.764015	-1.616320	0.012657
O	5.662859	-2.165369	-1.062495
O	4.989809	-2.122607	1.637928
O	2.349979	0.479471	-0.830578
O	3.216110	-2.218663	-2.807421
O	3.096409	-0.802711	3.294853
O	0.725857	4.186799	1.262049
O	-8.075012	-2.442765	0.196705
C	4.131771	-1.474289	0.705677
C	4.306018	-2.149204	-0.638357
C	2.669236	-1.634189	1.132549
C	3.513168	-1.392376	-1.688580
C	2.179183	-0.898568	-1.122025
C	2.250598	-0.596246	2.157323
C	1.245442	1.245458	-0.521432
C	-0.060595	0.710097	-0.477171
C	1.498404	2.550233	-0.241695
C	-1.159370	1.544645	-0.108399
C	0.397670	3.493389	0.078812
C	-0.950773	2.861991	0.165609
C	-2.525585	1.015220	-0.001777
C	-2.891110	-0.259181	-0.190393
C	-4.253648	-0.792550	-0.082515
C	-5.374578	-0.008593	0.225204
C	-4.450784	-2.159823	-0.297302
C	-6.636428	-0.569460	0.314313
C	-5.711067	-2.735475	-0.210698
C	-6.804385	-1.935420	0.096312
H	3.925040	-3.172283	-0.569503
H	4.402539	-0.417032	0.629653
H	2.566811	-2.627484	1.570311
H	4.093255	-0.524464	-2.004642
H	1.398497	-1.037023	-1.865794
H	1.202133	-0.740725	2.422481
H	2.393765	0.409990	1.763668
H	6.157945	-2.727682	-0.457395
H	4.797165	-1.739777	2.504193
H	4.049453	-2.538725	-3.170329
H	3.018207	-0.042229	3.877767
H	-0.229125	-0.329246	-0.696899
H	2.505467	2.941853	-0.258476
H	-1.771697	3.504252	0.456336
H	-3.269565	1.758692	0.259604
H	-2.145783	-1.003651	-0.446063
H	-0.057324	4.655105	1.577742
H	-5.268263	1.053512	0.398271
H	-3.599169	-2.784020	-0.536888
H	-7.499338	0.037863	0.551820
H	-5.849908	-3.795919	-0.380048
H	-8.060813	-3.393207	0.035668
O	0.434771	4.436053	-1.030790
O	-0.536651	5.450320	-0.806038
H	-0.023461	6.144601	-0.369159

ENERGIES
SCF ENERGY: -1528.36708370
SUM OF ELECTRONIC AND ZERO-POINT ENERGIES: -1527.944143
SUM OF ELECTRONIC AND THERMAL ENERGIES: -1527.914728
SUM OF ELECTRONIC AND THERMAL ENTHALPIES: -1527.913784
SUM OF ELECTRONIC AND THERMAL FREE ENERGIES: -1528.006777

==> AQ/RAF/C5PP <==
53
XYZ-COORDINATES + ENERGIES

O	2.217557	-1.263967	0.216722
O	6.008143	-2.237151	-0.950772
O	5.495187	-1.763855	1.742724
O	2.915071	0.640680	-0.936845
O	3.433578	-2.396013	-2.525788
O	3.786923	-0.104485	3.283485
O	1.698663	5.140809	-0.506861
O	-7.767454	-0.661403	0.939788
C	4.625319	-1.197737	0.768409
C	4.681291	-2.070070	-0.467809
C	3.182648	-1.196272	1.283467
C	3.871027	-1.427609	-1.579868
C	2.619650	-0.742715	-1.025877
C	2.886527	-0.003938	2.174044
C	1.880513	1.526060	-0.729890
C	0.558918	1.144283	-0.554408
C	2.264880	2.864584	-0.707606
C	-0.413910	2.135941	-0.362724
C	1.292388	3.830937	-0.517611
C	-0.043456	3.479874	-0.347269
C	-1.836125	1.819326	-0.165959
C	-2.358297	0.589093	-0.090150
C	-3.778419	0.269045	0.102161
C	-4.782205	1.285528	0.193455
C	-4.131419	-1.042472	0.222120
C	-6.110910	0.949804	0.463324
C	-5.540768	-1.487693	0.370521
C	-6.485914	-0.359616	0.599414
H	4.247023	-3.044825	-0.227084
H	4.954270	-0.183312	0.525448
H	3.044298	-2.112602	1.858042
H	4.486819	-0.672252	-2.069765
H	1.785207	-0.906866	-1.703412
H	1.850094	-0.045478	2.511748

H	3.059518	0.928011	1.635812
H	6.510381	-2.716424	-0.283217
H	5.378318	-1.249171	2.552224
H	4.210365	-2.858823	-2.858570
H	3.746532	0.713669	3.786987
H	0.278992	0.103729	-0.554626
H	3.301470	3.138235	-0.845165
H	-0.788166	4.252457	-0.200269
H	-2.478046	2.687406	-0.073566
H	-1.713083	-0.278580	-0.168971
H	0.937284	5.716494	-0.371932
H	-4.519588	2.324888	0.076787
H	-3.380290	-1.819584	0.173739
H	-6.857947	1.722574	0.586954
H	-7.902939	-1.616797	0.882342
H	-5.651954	-2.251882	1.144707
O	-5.851620	-2.170068	-0.895849
O	-7.074486	-2.887003	-0.721626
H	-7.681491	-2.409338	-1.304498

ENERGIES
SCF ENERGY: -1528.36754174
SUM OF ELECTRONIC AND ZERO-POINT ENERGIES: -1527.944033
SUM OF ELECTRONIC AND THERMAL ENERGIES: -1527.914720
SUM OF ELECTRONIC AND THERMAL ENTHALPIES: -1527.913776
SUM OF ELECTRONIC AND THERMAL FREE ENERGIES: -1528.006582

==> AQ/RAF/C6 <==
53
XYZ-COORDINATES + ENERGIES

O	2.032516	-1.532199	-0.110453
O	5.988611	-1.551576	-1.122770
O	5.264074	-1.889626	1.545968
O	2.428522	0.694588	-0.682048
O	3.574585	-1.680489	-2.927352
O	3.218357	-0.976226	3.279059
O	0.506370	4.635615	0.964688
O	-7.933734	-2.695897	0.196730
C	4.360128	-1.232204	0.664621
C	4.630726	-1.729241	-0.739854
C	2.915205	-1.590426	1.025967
C	3.777708	-0.948879	-1.724796
C	2.395498	-0.649453	-1.140742
C	2.370238	-0.730458	2.151497
C	1.249738	1.302175	-0.342777
C	0.014009	0.697930	-0.382876
C	1.430947	2.645429	0.122975
C	-1.134132	1.421111	-0.022617
C	0.355450	3.387570	0.438675
C	-1.038381	2.897148	0.211484
C	-2.444632	0.872286	0.074600
C	-2.802247	-0.414063	-0.199644
C	-4.141958	-0.969497	-0.089593
C	-5.266989	-0.227427	0.310916
C	-4.332802	-2.324203	-0.398078
C	-6.516298	-0.813481	0.399797
C	-5.580817	-2.922114	-0.311969
C	-6.674297	-2.163089	0.089324
H	4.362969	-2.788398	-0.794873
H	4.518480	-0.151023	0.714719
H	2.910663	-2.633777	1.342248
H	4.271765	-0.000300	-1.939105
H	1.638390	-0.760414	-1.913614
H	1.341001	-1.018110	2.370362
H	2.406414	0.324808	1.880275
H	6.534790	-2.106659	-0.556050
H	5.015039	-1.629421	2.442980
H	4.438308	-1.868514	-3.310383
H	3.009877	-0.336771	3.966064
H	-0.075356	-0.347124	-0.633580
H	2.433687	3.031514	0.243899
H	-3.208657	1.568875	0.401100
H	-2.046985	-1.116401	-0.531873
H	-0.350026	5.081241	0.997121
H	-5.171085	0.820992	0.556877
H	-3.482122	-2.916653	-0.710138
H	-7.378843	-0.238429	0.708428
H	-5.712003	-3.969812	-0.552265
H	-7.915259	-3.630618	-0.038846
H	-1.695976	3.221452	1.021001
O	-1.581445	3.495345	-1.013407
O	-1.771064	4.890013	-0.778682
H	-1.061944	5.292470	-1.299670

ENERGIES
SCF ENERGY: -1528.37564339
SUM OF ELECTRONIC AND ZERO-POINT ENERGIES: -1527.951895
SUM OF ELECTRONIC AND THERMAL ENERGIES: -1527.922846
SUM OF ELECTRONIC AND THERMAL ENTHALPIES: -1527.921902
SUM OF ELECTRONIC AND THERMAL FREE ENERGIES: -1528.013784

==> AQ/RAF/C6PP <==
53
XYZ-COORDINATES + ENERGIES

O	2.033126	-1.175727	-0.029068
O	5.895265	-1.859915	-1.158664
O	5.260376	-1.799536	1.551275
O	2.685841	0.898842	-0.874895
O	3.418167	-1.830921	-2.866991
O	3.447955	-0.405726	3.230035
O	1.275154	5.215910	0.256781
O	-7.990918	-1.088337	-0.287298
C	4.413815	-1.119547	0.630944
C	4.545600	-1.798891	-0.715640
C	2.952381	-1.226184	1.077850
C	3.764059	-1.014768	-1.754409
C	2.459067	-0.469885	-1.167781
C	2.586690	-0.173926	2.108757
C	1.608365	1.699395	-0.568814
C	0.299277	1.248504	-0.515524
C	1.936587	3.028000	-0.306682
C	-0.722117	2.161113	-0.200116
C	0.920035	3.915885	0.004634
C	-0.404343	3.498276	0.057139
C	-2.123408	1.773543	-0.115938
C	-2.611309	0.510556	-0.280611
C	-3.977041	0.127339	-0.200664
C	-5.049480	1.017788	-0.009430
C	-4.250507	-1.349823	-0.263835
C	-6.347519	0.583559	-0.000174
C	-5.685107	-1.727198	-0.397203
C	-6.649144	-0.806926	-0.227798
H	4.131361	-2.808596	-0.641721
H	4.722409	-0.072950	0.551883
H	2.820493	-2.215086	1.517781
H	4.370931	-0.169669	-2.081939
H	1.661082	-0.573117	-1.899166
H	1.539916	-0.284820	2.395043
H	2.753693	0.826792	1.710595
H	6.381271	-2.435379	-0.558608
H	5.089834	-1.413461	2.420762
H	4.232817	-2.179976	-3.245183
H	3.403714	0.356488	3.814147
H	0.064692	0.213703	-0.700617
H	2.965846	3.355036	-0.350122
H	-1.184214	4.208246	0.303468
H	-2.805476	2.583827	0.108359
H	-1.919247	-0.298436	-0.484519
H	0.490278	5.727640	0.483330
H	-4.853714	2.074541	0.106446
H	-3.641383	-1.818848	-1.038225
H	-7.169339	1.270652	0.140821
H	-5.934300	-2.761367	-0.597536
H	-8.115332	-2.033122	-0.436435
O	-3.701338	-1.847098	1.009224
O	-3.640734	-3.266111	0.942718
H	-4.463887	-3.543983	1.368260

ENERGIES
SCF ENERGY: -1528.37259068

SUM OF ELECTRONIC AND ZERO-POINT ENERGIES: -1527.949353
SUM OF ELECTRONIC AND THERMAL ENERGIES: -1527.920119
SUM OF ELECTRONIC AND THERMAL ENTHALPIES: -1527.919175
SUM OF ELECTRONIC AND THERMAL FREE ENERGIES: -1528.011678

==> PET <==
50
XYZ-COORDINATES + ENERGIES

O	1.806033	-1.235876	-0.018152
O	5.709101	-1.745208	-1.038825
O	5.010693	-1.798300	1.619104
O	2.346823	0.906253	-0.764998
O	3.350609	-1.627549	-2.857498
O	3.141626	-0.507338	3.279344
O	0.544946	5.132253	0.076546
O	-8.107782	-2.027666	0.163044
C	4.155560	-1.107809	0.721198
C	4.347147	-1.720961	-0.647826
C	2.686842	-1.291135	1.113903
C	3.589377	-0.894080	-1.666515
C	2.227368	-0.464183	-1.109429
C	2.252401	-0.297226	2.175601
C	1.205738	1.625487	-0.518774
C	-0.061629	1.063871	-0.448146
C	1.409839	2.991372	-0.336691
C	-1.163063	1.894556	-0.199800
C	0.314332	3.801362	-0.090665
C	-0.968636	3.264437	-0.022929
C	-2.536789	1.382284	-0.103872
C	-2.905111	0.097126	-0.178476
C	-4.272270	-0.423869	-0.084515
C	-5.404333	0.385955	0.084308
C	-4.467800	-1.804737	-0.167865
C	-6.671642	-0.160333	0.165099
C	-5.734492	-2.366922	-0.087359
C	-6.839948	-1.541609	0.078948
H	3.939492	-2.737670	-0.636904
H	4.422011	-0.045758	0.692429
H	2.585986	-2.301289	1.513086
H	4.169767	0.005253	-1.880877
H	1.466774	-0.593574	-1.877243
H	1.218224	-0.488936	2.465872
H	2.344213	0.723522	1.802326
H	6.205969	-2.134803	-0.311581
H	4.812204	-1.464683	2.502331
H	4.201351	-1.937999	-3.182944
H	2.983897	0.165348	3.946017
H	-0.193822	0.000682	-0.559679
H	2.404443	3.408693	-0.401714
H	-1.816521	3.910104	0.173866
H	-3.290538	2.146941	0.045674
H	-2.148644	-0.666776	-0.320459
H	-0.288975	5.589456	0.222756
H	-5.299756	1.460023	0.153007
H	-3.610198	-2.452682	-0.299282
H	-7.542546	0.467798	0.293909
H	-5.862533	-3.440733	-0.153516
H	-8.094519	-2.986971	0.087232

ENERGIES
SCF ENERGY: -1377.42301326
SUM OF ELECTRONIC AND ZERO-POINT ENERGIES: -1377.016916
SUM OF ELECTRONIC AND THERMAL ENERGIES: -1376.990628
SUM OF ELECTRONIC AND THERMAL ENTHALPIES: -1376.989684
SUM OF ELECTRONIC AND THERMAL FREE ENERGIES: -1377.075224

==> PET/AIP <==
50
XYZ-COORDINATES + ENERGIES

O	1.816825	-1.190157	0.105620
O	5.671597	-1.828594	-1.036872
O	5.084181	-1.677778	1.645931
O	2.289015	0.872307	-0.866929
O	3.281965	-1.778032	-2.778697
O	3.311750	-0.222230	3.269234
O	0.481339	5.097828	-0.109549
O	-8.043970	-2.003445	0.312344
C	4.202345	-1.043627	0.735536
C	4.330135	-1.758315	-0.591223
C	2.749447	-1.173184	1.201395
C	3.546663	-0.987282	-1.632580
C	2.194826	-0.535994	-1.069522
C	2.374406	-0.090619	2.197021
C	1.148215	1.569110	-0.613286
C	-0.106505	0.998576	-0.488691
C	1.326390	2.948534	-0.480873
C	-1.209628	1.840221	-0.235227
C	0.235802	3.772240	-0.224264
C	-1.031964	3.227932	-0.097254
C	-2.546416	1.345869	-0.099044
C	-2.937587	0.028085	-0.235435
C	-4.257990	-0.448648	-0.097542
C	-5.376772	0.379017	0.212748
C	-4.480771	-1.843277	-0.277472
C	-6.624009	-0.155181	0.341549
C	-5.728416	-2.386515	-0.152728
C	-6.809717	-1.542944	0.164389
H	3.909484	-2.765132	-0.492946
H	4.480327	0.009594	0.615754
H	2.650854	-2.147216	1.681322
H	4.119013	-0.097902	-1.903755
H	1.416347	-0.751261	-1.799550
H	1.352724	-0.243427	2.549456
H	2.460481	0.894653	1.735688
H	6.197422	-2.182754	-0.311652
H	4.940365	-1.268289	2.507485
H	4.125369	-2.105218	-3.108076
H	3.189759	0.497814	3.892772
H	-0.230841	-0.068654	-0.556792
H	2.315257	3.373338	-0.585373
H	-1.886111	3.861140	0.107895
H	-3.292862	2.096294	0.124090
H	-2.193532	-0.721550	-0.471431
H	-0.337883	5.579811	0.046302
H	-5.250235	1.442261	0.351595
H	-3.636829	-2.475984	-0.517919
H	-7.482751	0.456357	0.578857
H	-5.894845	-3.447187	-0.292379
H	-8.083854	-2.959878	0.175022

ENERGIES
SCF ENERGY: -1377.20594440
SUM OF ELECTRONIC AND ZERO-POINT ENERGIES: -1376.797902
SUM OF ELECTRONIC AND THERMAL ENERGIES: -1376.772271
SUM OF ELECTRONIC AND THERMAL ENTHALPIES: -1376.771327
SUM OF ELECTRONIC AND THERMAL FREE ENERGIES: -1376.854382

==> PET/BDE/C1P <==
49
XYZ-COORDINATES + ENERGIES

O	2.138771	-1.098900	0.733761
O	5.622719	-1.986395	-1.171654
O	5.723102	-0.723371	1.269889
O	2.054022	0.389722	-1.058198
O	2.867362	-2.835971	-1.922963
O	4.197941	1.195380	2.652143
O	-0.125919	4.460752	-1.746542
O	-7.851261	-2.138632	-0.727364
C	4.540950	-0.584879	0.496625
C	4.446216	-1.795125	-0.405003
C	3.299352	-0.570781	1.393066
C	3.295156	-1.600873	-1.370591
C	2.091083	-0.971926	-0.660109
C	3.011510	0.813080	1.945755
C	0.915774	1.113392	-0.816101
C	-0.162705	0.639459	-0.095298
C	0.923553	2.399877	-1.362594
C	-1.301139	1.481265	0.073013
C	-0.188338	3.220390	-1.188157
C	-1.297499	2.787855	-0.486384

C	-2.390661	1.030184	0.773859
C	-3.431535	0.605819	1.432533
C	-4.588832	-0.111757	0.856183
C	-4.673567	-0.410919	-0.507741
C	-5.632459	-0.508265	1.689615
C	-5.765436	-1.085463	-1.021717
C	-6.735311	-1.186552	1.185262
C	-6.800026	-1.475744	-0.172504
H	4.250614	-2.678401	0.212616
H	4.595230	0.325744	-0.109619
H	3.496605	-1.249109	2.224121
H	3.618883	-0.920395	-2.160423
H	1.181270	-1.479396	-0.975186
H	2.152360	0.775161	2.617210
H	2.810471	1.512922	1.133666
H	6.366873	-1.961239	-0.560839
H	5.725894	0.003311	1.904338
H	3.633025	-3.252487	-2.330772
H	4.113064	2.105464	2.945986
H	-0.163046	-0.335713	0.363656
H	1.778008	2.745426	-1.925965
H	-2.159052	3.429380	-0.356309
H	-3.479880	0.778881	2.508492
H	-0.949286	4.931368	-1.583096
H	-3.871261	-0.111179	-1.170474
H	-5.583444	-0.285733	2.748214
H	-5.833291	-1.319543	-2.075385
H	-7.540052	-1.489441	1.844593
H	-8.493383	-2.359921	-0.045668

\# ENERGIES
SCF ENERGY: -1376.74694118
SUM OF ELECTRONIC AND ZERO-POINT ENERGIES: -1376.355196
SUM OF ELECTRONIC AND THERMAL ENERGIES: -1376.328850
SUM OF ELECTRONIC AND THERMAL ENTHALPIES: -1376.327906
SUM OF ELECTRONIC AND THERMAL FREE ENERGIES: -1376.415041

==> PET/BDE/C2P <==
49
XYZ-COORDINATES + ENERGIES

O	1.237428	-1.161706	0.096525
O	4.823167	-2.558735	-1.221796
O	4.397309	-2.329158	1.485729
O	2.142666	0.771541	-0.840503
O	2.377124	-2.017243	-2.833393
O	3.016513	-0.585366	3.208881
O	1.376914	5.317753	-0.093383
O	-6.898698	-2.849518	0.004911
C	3.622480	-1.514569	0.619220
C	3.543151	-2.220186	-0.716038
C	2.194116	-1.356012	1.148339
C	2.881236	-1.297822	-1.719146
C	1.702538	-0.556600	-1.077744
C	2.085477	-0.243353	2.174805
C	1.208046	1.730368	-0.550554
C	-0.141088	1.466539	-0.357531
C	1.714257	3.025323	-0.456356
C	-1.002755	2.532299	-0.080004
C	0.846820	4.066825	-0.174389
C	-0.513878	3.831206	0.013728
C	-2.449543	2.304458	0.128796
C	-3.053859	1.148615	0.055884
C	-4.026513	0.169650	0.064830
C	-4.727382	-0.179878	-1.123666
C	-4.329222	-0.556462	1.247880
C	-5.673892	-1.178856	-1.119425
C	-5.278961	-1.556423	1.240254
C	-5.955578	-1.872771	0.060291
H	2.932439	-3.122156	-0.600637
H	4.103369	-0.538388	0.494706
H	1.921075	-2.300631	1.620787
H	3.615426	-0.557425	-2.042437
H	0.860044	-0.555994	-1.766435
H	1.067889	-0.195335	2.565564
H	2.350197	0.715039	1.726658
H	5.294663	-3.016054	-0.517504
H	4.360233	-1.918438	2.357898
H	3.106409	-2.521384	-3.207605
H	3.067636	0.135695	3.840712
H	-0.535692	0.462411	-0.391121
H	2.767080	3.211515	-0.614047
H	-1.186132	4.652739	0.232784
H	-3.033845	3.197383	0.359275
H	0.678322	5.954502	0.086484
H	-4.506332	0.353854	-2.037389
H	-3.803920	-0.315327	2.161309
H	-6.209260	-1.440298	-2.022095
H	-5.502500	-2.099392	2.151149
H	-6.999181	-3.256969	0.871147

\# ENERGIES
SCF ENERGY: -1376.74756964
SUM OF ELECTRONIC AND ZERO-POINT ENERGIES: -1376.355884
SUM OF ELECTRONIC AND THERMAL ENERGIES: -1376.330417
SUM OF ELECTRONIC AND THERMAL ENTHALPIES: -1376.329473
SUM OF ELECTRONIC AND THERMAL FREE ENERGIES: -1376.412572

==> PET/BDE/C4PP <==
49
XYZ-COORDINATES + ENERGIES

O	1.783007	-1.226715	0.021106
O	5.683627	-1.701828	-1.031845
O	5.015157	-1.704732	1.634257
O	2.277225	0.903277	-0.789280
O	3.308850	-1.658319	-2.828303
O	3.148284	-0.404321	3.283978
O	0.415821	5.114004	-0.021718
O	-8.034399	-2.129796	0.202731
C	4.139450	-1.049185	0.730739
C	4.326246	-1.690826	-0.625994
C	2.678360	-1.247210	1.143996
C	3.545742	-0.898041	-1.654381
C	2.181274	-0.480254	-1.094635
C	2.239175	-0.238597	2.189794
C	1.126849	1.603545	-0.546364
C	-0.128488	1.021068	-0.443835
C	1.304790	2.977930	-0.399577
C	-1.239681	1.842576	-0.200105
C	0.201032	3.778383	-0.154073
C	-1.069729	3.221508	-0.053140
C	-2.594913	1.318195	-0.079084
C	-2.961978	0.017364	-0.207334
C	-4.294004	-0.490682	-0.090266
C	-5.431134	0.326539	0.193456
C	-4.497308	-1.890507	-0.267723
C	-6.673175	-0.210292	0.291315
C	-5.732010	-2.450897	-0.174659
C	-6.901692	-1.637587	0.113023
H	3.934960	-2.713370	-0.587801
H	4.388287	0.016134	0.674356
H	2.597338	-2.250723	1.563610
H	4.109075	0.006495	-1.891295
H	1.416267	-0.643401	-1.851654
H	1.213535	-0.445186	2.499843
H	2.303906	0.774975	1.791796
H	6.195182	-2.070042	-0.303593
H	4.823674	-1.352277	2.511688
H	4.161450	-1.963344	-3.154256
H	2.981319	0.274790	3.941875
H	-0.242620	-0.047174	-0.520813
H	2.291238	3.410508	-0.488022
H	-1.927238	3.854359	0.142049
H	-3.349064	2.066376	0.128570
H	-2.205931	-0.728218	-0.421448
H	-0.420773	5.566660	0.124755
H	-5.304860	1.390871	0.333372
H	-3.634109	-2.507849	-0.483170
H	-7.538414	0.402459	0.506023
H	-5.886645	-3.512793	-0.310207

ENERGIES
SCF ENERGY: -1376.78213187
SUM OF ELECTRONIC AND ZERO-POINT ENERGIES: -1376.388751
SUM OF ELECTRONIC AND THERMAL ENERGIES: -1376.362831
SUM OF ELECTRONIC AND THERMAL ENTHALPIES: -1376.361887
SUM OF ELECTRONIC AND THERMAL FREE ENERGIES: -1376.447086

==> PET/BDE/C4PP/TS <==
53
XYZ-COORDINATES + ENERGIES

O	2.289945	-1.271042	-0.100530
O	6.188909	-1.980076	-1.035090
O	5.385954	-2.205578	1.583406
O	3.035976	0.872296	-0.635551
O	3.889141	-1.571398	-2.906547
O	3.546719	-0.944785	3.289976
O	1.582502	5.160790	0.499521
O	-7.629848	-1.068783	-0.787326
C	4.613905	-1.385210	0.720526
C	4.817582	-1.896501	-0.688314
C	3.122294	-1.499146	1.047448
C	4.153015	-0.943818	-1.661586
C	2.813491	-0.448555	-1.105720
C	2.713842	-0.579431	2.183528
C	1.953682	1.668916	-0.374789
C	0.639972	1.220780	-0.392799
C	2.265792	2.992440	-0.072003
C	-0.391184	2.130452	-0.121559
C	1.238491	3.877873	0.208470
C	-0.088394	3.457048	0.186619
C	-1.803504	1.740898	-0.136785
C	-2.289887	0.572403	-0.588684
C	-3.691809	0.181930	-0.603184
C	-4.722139	0.965543	-0.044454
C	-4.034925	-1.043513	-1.204250
C	-6.030921	0.547987	-0.097796
C	-5.340659	-1.477616	-1.254415
C	-6.367242	-0.684087	-0.705936
H	4.345475	-2.881340	-0.774032
H	4.950375	-0.345415	0.791412
H	2.936711	-2.532461	1.343219
H	4.805738	-0.079816	-1.798965
H	2.072432	-0.442255	-1.902899
H	1.659598	-0.728697	2.422303
H	2.882578	0.462359	1.907818
H	6.630923	-2.465198	-0.330208
H	5.173987	-1.936881	2.485423
H	4.721635	-1.921481	-3.238900
H	3.394698	-0.336522	4.017122
H	0.417338	0.182149	-0.572650
H	3.295388	3.320774	-0.063485
H	-0.885338	4.158313	0.404647
H	-2.484415	2.496042	0.238270
H	-1.610742	-0.164272	-1.002041
H	0.789624	5.684317	0.652415
H	-4.491187	1.907374	0.432684
H	-3.248773	-1.652916	-1.631318
H	-6.828636	1.151541	0.316432
H	-5.605906	-2.420968	-1.711614
H	-7.937760	-1.479728	0.176992
O	-7.933266	-1.927563	1.406314
O	-6.606863	-1.969261	1.680997
H	-6.405506	-1.122084	2.110117

ENERGIES
SCF ENERGY: -1528.34141121
SUM OF ELECTRONIC AND ZERO-POINT ENERGIES: -1527.923735
SUM OF ELECTRONIC AND THERMAL ENERGIES: -1527.894507
SUM OF ELECTRONIC AND THERMAL ENTHALPIES: -1527.893563
SUM OF ELECTRONIC AND THERMAL FREE ENERGIES: -1527.988109

==> PET/BDE/C5 <==
49
XYZ-COORDINATES + ENERGIES

O	1.748058	-1.155975	0.004298
O	5.623887	-1.855482	-1.020195
O	4.936511	-1.815094	1.640758
O	2.359813	0.937132	-0.814911
O	3.272778	-1.680714	-2.835771
O	3.132237	-0.411468	3.277002
O	0.530675	5.141205	0.064240
O	-8.031863	-2.053386	0.188746
C	4.106660	-1.112888	0.729677
C	4.266734	-1.767196	-0.624530
C	2.634300	-1.225812	1.133725
C	3.540613	-0.933657	-1.660420
C	2.195373	-0.443255	-1.113856
C	2.247709	-0.189976	2.173417
C	1.250159	1.695355	-0.558125
C	-0.045169	1.156142	-0.473078
C	1.464566	3.038252	-0.375511
C	-1.165657	1.988789	-0.208385
C	0.354594	3.919105	-0.103532
C	-0.967603	3.336223	-0.031863
C	-2.526623	1.450347	-0.110171
C	-2.871053	0.157967	-0.182889
C	-4.228187	-0.385513	-0.082118
C	-5.372530	0.405900	0.091967
C	-4.399387	-1.769671	-0.163731
C	-6.629188	-0.162360	0.180379
C	-5.655341	-2.353628	-0.075897
C	-6.773764	-1.546716	0.096480
H	3.817599	-2.765703	-0.588230
H	4.415964	-0.063409	0.673021
H	2.491384	-2.221703	1.554717
H	4.153993	-0.060889	-1.892059
H	1.430854	-0.573023	-1.877979
H	1.207436	-0.330252	2.472256
H	2.381917	0.816908	1.775261
H	6.108702	-2.251305	-0.288101
H	4.759461	-1.450698	2.516376
H	4.111009	-2.026134	-3.158841
H	3.005273	0.280485	3.930614
H	-0.189856	0.095951	-0.595208
H	2.458998	3.456717	-0.440337
H	-1.794368	4.003203	0.172570
H	-3.290081	2.203750	0.042995
H	-2.104498	-0.595514	-0.326217
H	-5.286999	1.481729	0.158908
H	-3.531374	-2.402625	-0.299570
H	-7.510219	0.450394	0.313632
H	-5.765294	-3.429455	-0.141280
H	-8.003918	-3.012623	0.114883

ENERGIES
SCF ENERGY: -1376.77200619
SUM OF ELECTRONIC AND ZERO-POINT ENERGIES: -1376.378841
SUM OF ELECTRONIC AND THERMAL ENERGIES: -1376.353008
SUM OF ELECTRONIC AND THERMAL ENTHALPIES: -1376.352064
SUM OF ELECTRONIC AND THERMAL FREE ENERGIES: -1376.437149

==> PET/BDE/C5/TS <==
53
XYZ-COORDINATES + ENERGIES

O	1.692521	-1.567711	0.172706
O	5.543004	-2.552874	-0.702935
O	4.911855	-1.794049	1.864484
O	2.293092	0.218780	-1.193814
O	3.155521	-2.857257	-2.447716
O	3.151526	0.025166	3.092900
O	0.611518	4.589847	-1.177198
O	-8.178847	-2.110015	0.569463
C	4.067848	-1.351997	0.813240
C	4.195490	-2.348292	-0.317190
C	2.603257	-1.334149	1.259994
C	3.449596	-1.820474	-1.525239
C	2.117776	-1.189326	-1.105305
C	2.246458	-0.049580	1.986007
C	1.196957	1.028382	-1.080839
C	-0.101437	0.557041	-0.891213

C	1.452707	2.387281	-1.162376
C	-1.180248	1.458389	-0.793171
C	0.383499	3.296116	-1.075386
C	-0.934220	2.818789	-0.896372
C	-2.561836	1.006960	-0.588567
C	-2.938212	-0.233052	-0.249973
C	-4.314220	-0.696081	-0.051047
C	-5.442784	0.078820	-0.353603
C	-4.522228	-1.979505	0.460434
C	-6.720574	-0.403101	-0.140605
C	-5.799631	-2.475723	0.679640
C	-6.901948	-1.683759	0.380177
H	3.743717	-3.295393	-0.002551
H	4.383195	-0.360013	0.471791
H	2.464728	-2.175378	1.940069
H	4.060489	-1.049085	-1.997943
H	1.338916	-1.516141	-1.791720
H	1.211244	-0.089884	2.329574
H	2.380846	0.810032	1.328183
H	6.042990	-2.744434	0.097647
H	4.755024	-1.205648	2.612822
H	3.986340	-3.284206	-2.679484
H	3.063128	0.881881	3.517793
H	-0.284976	-0.502149	-0.815751
H	2.460207	2.753320	-1.300295
H	-1.741704	3.537899	-0.842636
H	-3.309909	1.781511	-0.712879
H	-2.183376	-0.994010	-0.082610
H	-5.326149	1.068954	-0.772435
H	-3.666722	-2.601080	0.693535
H	-7.589391	0.195185	-0.378809
H	-5.938935	-3.473173	1.078770
H	-8.175753	-3.005812	0.921443
H	0.668028	5.024483	-0.132672
O	0.670907	5.175013	1.103853
O	0.667570	3.893958	1.559785
H	-0.268767	3.675847	1.694297

ENERGIES
SCF ENERGY: -1528.33748481
SUM OF ELECTRONIC AND ZERO-POINT ENERGIES: -1527.920097
SUM OF ELECTRONIC AND THERMAL ENERGIES: -1527.891003
SUM OF ELECTRONIC AND THERMAL ENTHALPIES: -1527.890058
SUM OF ELECTRONIC AND THERMAL FREE ENERGIES: -1527.983079

==> PET/RAF/C1 <==
53
XYZ-COORDINATES + ENERGIES

O	2.294436	-0.868430	1.109889
O	5.243776	-2.820860	-0.899385
O	5.923717	-0.762633	0.789787
O	1.903856	-0.151763	-1.076392
O	2.350949	-3.545121	-0.729818
O	4.821602	1.691384	1.596115
O	-0.102449	3.621369	-2.954073
O	-7.910602	-2.336274	0.297873
C	4.607477	-0.778444	0.259504
C	4.269777	-2.216345	-0.066471
C	3.593258	-0.285861	1.295566
C	2.945244	-2.258504	-0.801441
C	1.956338	-1.260027	-0.189910
C	3.493320	1.228107	1.324856
C	0.895388	0.762250	-0.914946
C	0.005903	0.762735	0.107220
C	0.860264	1.738834	-1.943272
C	-1.053481	1.810463	0.208240
C	-0.128070	2.743847	-1.910322
C	-1.041907	2.808095	-0.906721
C	-2.448900	1.248538	0.344070
C	-2.784953	-0.011774	0.067193
C	-4.133650	-0.591263	0.139981
C	-5.258020	0.118165	0.582383
C	-4.313263	-1.919611	-0.249596
C	-6.506552	-0.474228	0.629445
C	-5.561275	-2.527479	-0.208260
C	-6.660958	-1.801434	0.232245
H	4.176354	-2.774110	0.871678
H	4.562829	-0.170116	-0.650295
H	3.934303	-0.636443	2.270477
H	3.118923	-1.975010	-1.841284
H	0.973005	-1.721462	-0.115174
H	2.801304	1.540869	2.108059
H	3.148278	1.605477	0.361161
H	6.102972	-2.657422	-0.496135
H	6.090665	0.138537	1.091301
H	2.993424	-4.180890	-1.060222
H	4.835898	2.650949	1.568083
H	0.060505	0.057223	0.921309
H	1.576137	1.700004	-2.750283
H	-1.818491	3.562597	-0.911142
H	-3.189025	1.977974	0.648970
H	-2.011921	-0.699977	-0.259249
H	-0.824701	4.251196	-2.864119
H	-5.161300	1.147509	0.899672
H	-3.459504	-2.490000	-0.593566
H	-7.372234	0.075341	0.973333
H	-5.678096	-3.559608	-0.516685
H	-7.888179	-3.252586	0.004710
O	-0.690776	2.471768	1.478867
O	-1.617305	3.517617	1.747169
H	-1.124405	4.297112	1.460553

ENERGIES
SCF ENERGY: -1528.33720414
SUM OF ELECTRONIC AND ZERO-POINT ENERGIES: -1527.914683
SUM OF ELECTRONIC AND THERMAL ENERGIES: -1527.885510
SUM OF ELECTRONIC AND THERMAL ENTHALPIES: -1527.884566
SUM OF ELECTRONIC AND THERMAL FREE ENERGIES: -1527.977086

==> PET/RAF/C1P <==
53
XYZ-COORDINATES + ENERGIES

O	2.106456	-1.067067	-0.710433
O	6.169195	-1.060556	-0.957158
O	4.958436	-2.514160	1.038603
O	2.588176	1.122927	-0.076213
O	4.222124	0.071199	-2.910240
O	2.679989	-2.272588	2.666094
O	0.250302	4.110533	2.644310
O	-7.823026	-2.278104	-0.085406
C	4.239260	-1.426954	0.477878
C	4.761030	-1.215604	-0.925791
C	2.746387	-1.752405	0.376829
C	4.147536	0.047890	-1.493790
C	2.667407	0.151561	-1.109448
C	2.013339	-1.479183	1.677458
C	1.357671	1.605535	0.277683
C	0.158348	1.169284	-0.278099
C	1.377508	2.588620	1.262906
C	-1.031043	1.749559	0.164216
C	0.183134	3.148300	1.686325
C	-1.030790	2.738585	1.135573
C	-2.341630	1.271206	-0.417718
C	-2.680048	-0.110247	0.023079
C	-3.985027	-0.645330	-0.018300
C	-5.100045	0.069210	-0.526432
C	-4.221597	-1.956685	0.463110
C	-6.360811	-0.487102	-0.539246
C	-5.482431	-2.514774	0.449014
C	-6.560411	-1.779616	-0.049684
H	4.460817	-2.068986	-1.543569
H	4.401981	-0.523028	1.074579
H	2.665189	-2.814235	0.140819
H	4.671658	0.905910	-1.068462
H	2.093470	0.475895	-1.975352
H	0.966240	-1.772191	1.583243
H	2.074840	-0.420878	1.934294
H	6.547576	-1.796591	-0.464635
H	4.552872	-2.703936	1.893219
H	5.146111	-0.040600	-3.154932

H	2.325092	-2.063898	3.533496
H	0.124303	0.381689	-1.015689
H	2.317341	2.919987	1.681590
H	-1.961655	3.187496	1.462104
H	-3.130519	1.978699	-0.155587
H	-1.872680	-0.738388	0.373122
H	-0.633996	4.434513	2.842701
H	-4.963887	1.061953	-0.931822
H	-3.388321	-2.529118	0.849677
H	-7.206754	0.059911	-0.932751
H	-5.640269	-3.519626	0.822464
H	-7.831520	-3.174797	0.264605
O	-2.267891	1.220222	-1.861693
O	-2.099524	2.550744	-2.337467
H	-1.138500	2.663921	-2.309694

ENERGIES
SCF ENERGY: -1528.37728278
SUM OF ELECTRONIC AND ZERO-POINT ENERGIES: -1527.953202
SUM OF ELECTRONIC AND THERMAL ENERGIES: -1527.924249
SUM OF ELECTRONIC AND THERMAL ENTHALPIES: -1527.923305
SUM OF ELECTRONIC AND THERMAL FREE ENERGIES: -1528.015312

==> PET/RAF/C1P/TS <==
53
XYZ-COORDINATES + ENERGIES

O	-1.996212	-1.253701	0.347924
O	-5.955426	-1.391458	1.272044
O	-5.135478	-2.259205	-1.204639
O	-2.515173	1.018924	0.389776
O	-3.686844	-0.750693	3.090178
O	-3.161064	-1.549832	-3.078548
O	-0.571737	4.708885	-1.710103
O	7.720825	-2.534789	-0.040372
C	-4.306619	-1.336166	-0.515448
C	-4.577050	-1.502287	0.963073
C	-2.826041	-1.643575	-0.757056
C	-3.847486	-0.412706	1.721745
C	-2.449198	-0.187581	1.135131
C	-2.318750	-1.023911	-2.046336
C	-1.346530	1.601076	-0.023804
C	-0.088966	1.042613	0.168765
C	-1.503381	2.827993	-0.664428
C	1.035412	1.735923	-0.294133
C	-0.378918	3.508262	-1.101008
C	0.894523	2.974960	-0.912501
C	2.391686	1.194941	-0.105934
C	2.653955	-0.159113	-0.160955
C	3.966471	-0.751100	-0.116296
C	5.137637	-0.005697	0.118975
C	4.097532	-2.136146	-0.308364
C	6.376214	-0.613190	0.140832
C	5.335391	-2.753538	-0.290126
C	6.479352	-1.989188	-0.069213
H	-4.195680	-2.478416	1.282054
H	-4.548127	-0.313390	-0.824228
H	-2.731287	-2.728101	-0.824681
H	-4.413474	0.515313	1.620814
H	-1.730736	-0.089090	1.946527
H	-1.277187	-1.304728	-2.211301
H	-2.401734	0.062786	-2.005089
H	-6.428593	-1.982996	0.677312
H	-4.888804	-2.211789	-2.136136
H	-4.560591	-0.935956	3.448483
H	-2.953317	-1.116349	-3.909708
H	0.030302	0.092077	0.663208
H	-2.489577	3.249407	-0.798599
H	1.769064	3.516177	-1.253362
H	3.193636	1.887992	-0.320197
H	1.820740	-0.849795	-0.206660
H	0.278320	5.094569	-1.944326
H	5.071782	1.056449	0.308124
H	3.209254	-2.731082	-0.478762
H	7.275512	-0.042768	0.328499
H	5.418111	-3.822702	-0.444513
H	7.665451	-3.485367	-0.182815
O	2.623206	1.561467	1.862882
O	2.324497	2.886015	2.047044
H	1.362775	2.903433	2.158350

ENERGIES
SCF ENERGY: -1528.34549141
SUM OF ELECTRONIC AND ZERO-POINT ENERGIES: -1527.923114
SUM OF ELECTRONIC AND THERMAL ENERGIES: -1527.894054
SUM OF ELECTRONIC AND THERMAL ENTHALPIES: -1527.893109
SUM OF ELECTRONIC AND THERMAL FREE ENERGIES: -1527.986159

==> PET/RAF/C1PP <==
53
XYZ-COORDINATES + ENERGIES

O	2.273995	-1.180869	-0.030903
O	6.112306	-1.343867	-1.369521
O	5.642914	-1.431116	1.337347
O	2.562682	0.994237	-0.822456
O	3.605262	-1.469010	-2.983498
O	3.808039	-0.312033	3.143575
O	0.467834	5.059042	0.123877
O	-7.585952	-0.201607	-1.453952
C	4.655283	-0.834312	0.511370
C	4.790813	-1.441758	-0.867061
C	3.247711	-1.151818	1.024055
C	3.878129	-0.700205	-1.822482
C	2.535245	-0.385265	-1.153749
C	2.810708	-0.198727	2.121369
C	1.387233	1.613074	-0.486464
C	0.184413	0.943885	-0.303691
C	1.480638	2.994441	-0.334793
C	-0.958559	1.681161	0.027326
C	0.341986	3.710893	-0.008138
C	-0.879025	3.064684	0.170564
C	-2.267730	1.044824	0.248552
C	-2.503510	-0.265467	0.295476
C	-3.873422	-0.875589	0.470604
C	-4.915321	0.074808	0.960426
C	-4.261623	-1.564721	-0.801009
C	-6.095897	0.256662	0.324693
C	-5.455511	-1.349311	-1.415682
C	-6.388050	-0.442985	-0.870936
H	4.482205	-2.491772	-0.818991
H	4.816102	0.247688	0.457817
H	3.273853	-2.165641	1.425599
H	4.355367	0.243755	-2.092185
H	1.727986	-0.585528	-1.855778
H	1.829296	-0.490072	2.498845
H	2.768490	0.823544	1.743270
H	6.701385	-1.677786	-0.684622
H	5.485574	-1.110290	2.233618
H	4.449351	-1.704068	-3.381408
H	3.624854	0.325304	3.837897
H	0.132982	-0.128212	-0.395611
H	2.425174	3.497062	-0.486925
H	-1.763850	3.635585	0.427381
H	-3.095741	1.733001	0.380644
H	-1.711045	-0.993586	0.161361
H	-0.387841	5.446192	0.333426
H	-4.697042	0.595885	1.882895
H	-3.535775	-2.247115	-1.224917
H	-6.842873	0.934292	0.717328
H	-5.694689	-1.866193	-2.338544
H	-7.677488	-0.732052	-2.252731
O	-3.606219	-1.888685	1.508193
O	-4.797189	-2.610143	1.775674
H	-4.816715	-3.263206	1.062374

ENERGIES
SCF ENERGY: -1528.34281407
SUM OF ELECTRONIC AND ZERO-POINT ENERGIES: -1527.919546
SUM OF ELECTRONIC AND THERMAL ENERGIES: -1527.890559
SUM OF ELECTRONIC AND THERMAL ENTHALPIES: -1527.889615
SUM OF ELECTRONIC AND THERMAL FREE ENERGIES: -1527.981022

==> PET/RAF/C2 <==

53

XYZ-COORDINATES + ENERGIES

O	1.926315	-1.133809	0.599267
O	5.536033	-2.299646	-0.887135
O	5.380036	-1.454887	1.722988
O	2.247905	0.568907	-0.960312
O	2.941622	-2.498694	-2.253511
O	3.933426	0.468723	3.166107
O	0.714505	4.866110	0.077684
O	-8.177272	-1.989702	0.827050
C	4.383436	-1.006383	0.818783
C	4.280452	-2.029267	-0.289175
C	3.019471	-0.917637	1.506872
C	3.373760	-1.478513	-1.367994
C	2.123413	-0.838538	-0.754082
C	2.842709	0.397696	2.241015
C	1.193225	1.372094	-0.620986
C	-0.192316	0.801360	-0.639048
C	1.439893	2.680027	-0.406746
C	-1.241071	1.762503	-0.158703
C	0.367338	3.566457	-0.101786
C	-0.927818	3.104221	0.039590
C	-2.569888	1.294494	0.071756
C	-3.009790	0.024145	-0.118711
C	-4.357067	-0.467708	0.141583
C	-5.377649	0.310939	0.711790
C	-4.664579	-1.792914	-0.191098
C	-6.639909	-0.207399	0.931305
C	-5.927392	-2.324588	0.024703
C	-6.919218	-1.529095	0.587641
H	3.847293	-2.950633	0.114902
H	4.669574	-0.035731	0.397927
H	2.966730	-1.736827	2.225120
H	3.921746	-0.707255	-1.913099
H	1.245980	-1.217611	-1.269978
H	1.884609	0.409407	2.763421
H	2.884576	1.229641	1.537638
H	6.154421	-2.496600	-0.175550
H	5.373665	-0.843439	2.469281
H	3.727984	-2.931462	-2.601054
H	3.922419	1.321860	3.606496
H	2.450561	3.062841	-0.453838
H	-1.714652	3.791192	0.331875
H	-3.263262	2.047573	0.429587
H	-2.327711	-0.715116	-0.519908
H	-0.070431	5.388946	0.273133
H	-5.187210	1.337188	0.993464
H	-3.896844	-2.416705	-0.631579
H	-7.421589	0.396920	1.371279
H	-6.142187	-3.352368	-0.243715
H	-8.246872	-2.907118	0.544904
H	-0.236825	-0.123136	-0.058241
O	-0.482756	0.297489	-1.976921
O	-0.531491	1.388760	-2.880374
H	0.369419	1.411060	-3.231052

ENERGIES

SCF ENERGY: -1528.35704545

SUM OF ELECTRONIC AND ZERO-POINT ENERGIES: -1527.933148

SUM OF ELECTRONIC AND THERMAL ENERGIES: -1527.904084

SUM OF ELECTRONIC AND THERMAL ENTHALPIES: -1527.903140

SUM OF ELECTRONIC AND THERMAL FREE ENERGIES: -1527.994985

==> PET/RAF/C2P <==

53

XYZ-COORDINATES + ENERGIES

O	2.038622	-1.225834	-0.123802
O	5.976157	-1.566611	-1.078478
O	5.233534	-1.698481	1.564203
O	2.537389	0.953434	-0.803787
O	3.625918	-1.526272	-2.931641
O	3.277103	-0.549952	3.220496
O	0.612820	5.168553	-0.206704
O	-8.041641	-1.613613	-0.359405
C	4.364975	-1.027837	0.663890
C	4.607255	-1.605512	-0.713053
C	2.899152	-1.285408	1.023106
C	3.832728	-0.794612	-1.733129
C	2.453706	-0.411242	-1.184380
C	2.398700	-0.347861	2.106159
C	1.374499	1.645965	-0.583746
C	0.136972	1.044510	-0.430344
C	1.530231	3.029984	-0.503158
C	-1.000244	1.856642	-0.205435
C	0.411181	3.823222	-0.274234
C	-0.844070	3.258012	-0.129987
C	-2.293634	1.302820	-0.042143
C	-2.575417	-0.159005	-0.115765
C	-4.039606	-0.506558	-0.201845
C	-5.002032	0.114524	0.598251
C	-4.450183	-1.520311	-1.064283
C	-6.334209	-0.265480	0.539182
C	-5.781156	-1.906987	-1.139050
C	-6.723834	-1.278160	-0.332183
H	4.242792	-2.638496	-0.726633
H	4.584038	0.045195	0.661498
H	2.833584	-2.312097	1.385921
H	4.386928	0.123490	-1.936351
H	1.702982	-0.533525	-1.963426
H	1.369588	-0.597723	2.368342
H	2.448660	0.687290	1.765978
H	6.474839	-1.948072	-0.348158
H	4.995148	-1.401959	2.450959
H	4.487871	-1.811825	-3.250205
H	3.051139	0.069620	3.918220
H	0.043843	-0.028365	-0.425501
H	2.506273	3.476641	-0.626733
H	-1.709548	3.885173	0.047538
H	-3.114091	1.983744	0.129082
H	-0.226897	5.614270	-0.058066
H	-4.713582	0.904215	1.279108
H	-3.719105	-2.015640	-1.691202
H	-7.080304	0.213615	1.158221
H	-6.086759	-2.692013	-1.820138
H	-8.185598	-2.320301	-0.996685
H	-2.063420	-0.596782	-0.976467
O	-1.932942	-0.890946	0.966456
O	-2.431677	-0.413201	2.205163
H	-3.180727	-1.003318	2.367572

ENERGIES

SCF ENERGY: -1528.37357156

SUM OF ELECTRONIC AND ZERO-POINT ENERGIES: -1527.949287

SUM OF ELECTRONIC AND THERMAL ENERGIES: -1527.920423

SUM OF ELECTRONIC AND THERMAL ENTHALPIES: -1527.919478

SUM OF ELECTRONIC AND THERMAL FREE ENERGIES: -1528.011548

==> PET/RAF/C2P/TS <==

53

XYZ-COORDINATES + ENERGIES

O	2.026770	-1.223115	-0.092162
O	5.949013	-1.730114	-1.035028
O	5.204912	-1.742100	1.611152
O	2.577994	0.910884	-0.862008
O	3.615695	-1.659710	-2.894637
O	3.299932	-0.445798	3.217681
O	0.759089	5.167019	-0.238746
O	-7.929020	-1.841007	-0.413983
C	4.360629	-1.073132	0.687058
C	4.580160	-1.707612	-0.668325
C	2.886685	-1.261404	1.056806
C	3.835493	-0.902881	-1.714532
C	2.464333	-0.466454	-1.185916
C	2.424501	-0.259259	2.098492
C	1.433439	1.633479	-0.650644
C	0.173484	1.067134	-0.527169
C	1.626199	3.010209	-0.551140
C	-0.932140	1.906390	-0.307970

C	0.529688	3.828845	-0.328180
C	-0.745191	3.290019	-0.205262
C	-2.278610	1.393581	-0.166336
C	-2.637938	0.062457	-0.264066
C	-4.030667	-0.398054	-0.314431
C	-5.099507	0.335108	0.216267
C	-4.302966	-1.651573	-0.868778
C	-6.393610	-0.157283	0.175351
C	-5.594236	-2.153138	-0.919947
C	-6.642926	-1.402224	-0.396414
H	4.178542	-2.726581	-0.647393
H	4.619797	-0.009715	0.645193
H	2.784146	-2.267777	1.464782
H	4.416866	-0.006388	-1.937746
H	1.714497	-0.605492	-1.962942
H	1.388126	-0.458823	2.374264
H	2.511448	0.758041	1.714377
H	6.435061	-2.104448	-0.292571
H	4.984679	-1.398943	2.485704
H	4.471726	-1.974268	-3.201739
H	3.087404	0.198502	3.897023
H	0.051755	-0.002573	-0.551834
H	2.616695	3.430031	-0.653347
H	-1.594947	3.937227	-0.024114
H	-3.035637	2.126807	0.078610
H	-0.072357	5.630307	-0.097107
H	-4.926118	1.298977	0.674464
H	-3.488094	-2.240428	-1.270785
H	-7.218536	0.408898	0.585649
H	-5.787932	-3.123211	-1.361576
H	-7.974474	-2.705382	-0.835002
H	-1.927359	-0.631406	-0.691729
O	-2.102605	-0.578029	1.546945
O	-3.074979	-0.259466	2.453951
H	-3.790604	-0.887115	2.273320

ENERGIES
SCF ENERGY: -1528.34549298
SUM OF ELECTRONIC AND ZERO-POINT ENERGIES: -1527.923097
SUM OF ELECTRONIC AND THERMAL ENERGIES: -1527.894088
SUM OF ELECTRONIC AND THERMAL ENTHALPIES: -1527.893143
SUM OF ELECTRONIC AND THERMAL FREE ENERGIES: -1527.985466

==> PET/RAF/C2PP <==
53
XYZ-COORDINATES + ENERGIES

O	2.330628	-1.260198	0.111101
O	6.290229	-1.587146	-0.774991
O	5.530402	-1.347461	1.855666
O	2.756174	0.800086	-0.894614
O	3.953685	-1.934239	-2.613602
O	3.527447	-0.020423	3.307701
O	0.712948	4.984306	-0.563250
O	-7.613586	-2.470341	-0.255107
C	4.645968	-0.854608	0.861107
C	4.921543	-1.627082	-0.409710
C	3.187326	-1.106698	1.253559
C	4.122833	-1.016832	-1.544070
C	2.726049	-0.608076	-1.063875
C	2.648901	-0.028448	2.176103
C	1.571720	1.473591	-0.750186
C	0.336900	0.852568	-0.632401
C	1.695137	2.861298	-0.723056
C	-0.814461	1.645200	-0.498121
C	0.553252	3.633557	-0.583757
C	-0.698840	3.038132	-0.474012
C	-2.150035	1.077162	-0.370489
C	-2.457470	-0.249474	-0.350070
C	-3.768198	-0.781552	-0.240031
C	-4.955731	0.129058	-0.092436
C	-3.961044	-2.169528	-0.225080
C	-6.279529	-0.547778	-0.210745
C	-5.213846	-2.731061	-0.182010
C	-6.381249	-1.889150	-0.207419
H	4.599958	-2.664199	-0.264142
H	4.827028	0.212501	0.694330
H	3.156666	-2.065339	1.773083
H	4.640750	-0.119590	-1.887707
H	1.990903	-0.880082	-1.819037
H	1.628024	-0.270030	2.476066
H	2.662953	0.941205	1.676795
H	6.798870	-1.834526	0.004553
H	5.282228	-0.919593	2.684061
H	4.828908	-2.223758	-2.889645
H	3.287475	0.703962	3.890450
H	0.265460	-0.222138	-0.617279
H	2.666845	3.324169	-0.819467
H	-1.585333	3.651354	-0.362502
H	-2.948303	1.801439	-0.272092
H	-1.663611	-0.983447	-0.427162
H	-0.144686	5.411383	-0.472415
H	-3.090251	-2.811624	-0.264524
H	-7.170431	0.061075	-0.277594
H	-5.335027	-3.806783	-0.163523
H	-7.522990	-3.424060	-0.339324
H	-4.891793	0.963380	-0.794011
O	-4.786795	0.714164	1.238822
O	-5.726753	1.772211	1.367304
H	-6.454012	1.345350	1.839051

ENERGIES
SCF ENERGY: -1528.35347938
SUM OF ELECTRONIC AND ZERO-POINT ENERGIES: -1527.930148
SUM OF ELECTRONIC AND THERMAL ENERGIES: -1527.900968
SUM OF ELECTRONIC AND THERMAL ENTHALPIES: -1527.900023
SUM OF ELECTRONIC AND THERMAL FREE ENERGIES: -1527.992281

==> PET/RAF/C3 <==
53
XYZ-COORDINATES + ENERGIES

O	1.155718	-1.298879	-0.547496
O	4.924567	-2.750295	-0.036361
O	3.122584	-2.966762	2.028143
O	2.405847	0.656423	-0.393175
O	3.621821	-1.766308	-2.563505
O	0.999376	-1.468706	3.079985
O	1.363305	4.290974	2.046542
O	-7.930966	-1.573249	-0.352926
C	2.908457	-1.946618	1.062961
C	3.563648	-2.398476	-0.222918
C	1.412983	-1.762470	0.785037
C	3.505782	-1.272557	-1.236716
C	2.171861	-0.523082	-1.138310
C	0.753424	-0.853920	1.807520
C	1.565039	1.770356	-0.675531
C	0.109999	1.423980	-0.721090
C	1.920880	2.811730	0.327249
C	-0.799516	1.961928	0.149198
C	0.980900	3.348488	1.137322
C	-0.380413	2.942283	1.076584
C	-2.212359	1.551986	0.160289
C	-2.648086	0.363349	-0.275334
C	-4.035528	-0.109237	-0.293506
C	-5.132825	0.695412	0.042789
C	-4.286001	-1.432311	-0.664929
C	-6.421350	0.195218	0.020179
C	-5.574726	-1.947167	-0.693550
C	-6.644909	-1.131119	-0.346884
H	3.004215	-3.255251	-0.614215
H	3.360500	-1.009232	1.402803
H	0.954960	-2.751097	0.846237
H	4.308266	-0.565354	-1.020426
H	1.823773	-0.286752	-2.141083
H	-0.318087	-0.785955	1.611942
H	1.199147	0.140572	1.771659
H	4.959271	-3.353755	0.713349
H	2.613294	-2.717843	2.808880
H	4.452037	-2.248862	-2.622566
H	0.700800	-0.881416	3.778129
H	-0.198049	0.739633	-1.497008

H	2.959643	3.092526	0.416613
H	-1.101523	3.385940	1.751714
H	-2.905193	2.268947	0.585947
H	-1.920440	-0.354047	-0.642148
H	0.600446	4.588628	2.550935
H	-4.983291	1.730122	0.319781
H	-3.454526	-2.071125	-0.935565
H	-7.266825	0.818414	0.277836
H	-5.747548	-2.976423	-0.984497
H	-7.957576	-2.494953	-0.628734
O	1.817272	2.201712	-2.026635
O	3.178722	2.588632	-2.157390
H	3.107166	3.552307	-2.169214

ENERGIES
SCF ENERGY: -1528.34638521
SUM OF ELECTRONIC AND ZERO-POINT ENERGIES: -1527.923943
SUM OF ELECTRONIC AND THERMAL ENERGIES: -1527.894771
SUM OF ELECTRONIC AND THERMAL ENTHALPIES: -1527.893827
SUM OF ELECTRONIC AND THERMAL FREE ENERGIES: -1527.985563

==> PET/RAF/C3PP <==
53
XYZ-COORDINATES + ENERGIES

O	2.464265	-1.218660	-0.165197
O	6.414759	-1.527547	-1.080366
O	5.626681	-1.833232	1.535617
O	2.967424	0.994653	-0.701115
O	4.117006	-1.319315	-2.967823
O	3.658678	-0.753104	3.232414
O	1.016212	5.066634	0.494176
O	-7.446884	-2.220340	0.040741
C	4.781799	-1.091355	0.669585
C	5.040163	-1.576169	-0.739433
C	3.307119	-1.351616	0.989197
C	4.292538	-0.685300	-1.710601
C	2.900013	-0.342985	-1.168719
C	2.803079	-0.471492	2.118287
C	1.798809	1.653874	-0.420565
C	0.545839	1.056058	-0.451057
C	1.954691	2.999340	-0.096161
C	-0.583429	1.829231	-0.154072
C	0.828825	3.753984	0.188543
C	-0.439544	3.180455	0.156921
C	-1.939665	1.262530	-0.142836
C	-2.244557	-0.039548	-0.145509
C	-3.614994	-0.576983	-0.134989
C	-4.679266	0.117179	-0.637156
C	-3.810248	-1.878706	0.405266
C	-6.074874	-0.391771	-0.578009
C	-5.092579	-2.451365	0.467673
C	-6.168760	-1.763773	-0.003942
H	4.664633	-2.601111	-0.831591
H	5.014615	-0.023750	0.743399
H	3.224921	-2.396387	1.291925
H	4.851915	0.244935	-1.825923
H	2.170825	-0.424045	-1.972769
H	1.766025	-0.719317	2.350057
H	2.873595	0.580994	1.841464
H	6.898107	-1.969026	-0.373978
H	5.385967	-1.585598	2.436440
H	4.987311	-1.578662	-3.286275
H	3.455374	-0.147519	3.949215
H	0.439843	0.010887	-0.690099
H	2.937905	3.447775	-0.086935
H	-1.313273	3.779364	0.385324
H	-2.742200	1.990897	-0.093348
H	-1.454891	-0.782472	-0.116906
H	0.165709	5.485785	0.658431
H	-4.545461	1.080112	-1.109316
H	-2.960328	-2.428008	0.783405
H	-5.226378	-3.438376	0.894504
H	-7.482640	-3.079047	0.479174
H	-6.560625	-0.350823	-1.557808
O	-6.830388	0.531241	0.266311
O	-8.196485	0.509405	-0.128760
H	-8.510848	-0.324005	0.249072

ENERGIES
SCF ENERGY: -1528.34749632
SUM OF ELECTRONIC AND ZERO-POINT ENERGIES: -1527.923310
SUM OF ELECTRONIC AND THERMAL ENERGIES: -1527.894451
SUM OF ELECTRONIC AND THERMAL ENTHALPIES: -1527.893507
SUM OF ELECTRONIC AND THERMAL FREE ENERGIES: -1527.985536

==> PET/RAF/C4 <==
53
XYZ-COORDINATES + ENERGIES

O	1.509620	-1.684752	-0.145731
O	5.440787	-2.247358	-1.043175
O	4.586808	-2.689728	1.531239
O	2.206715	0.513045	-0.506573
O	3.184790	-1.723654	-2.930640
O	2.688591	-1.597656	3.286348
O	0.674174	4.754839	0.807661
O	-8.426043	-1.748992	-0.111335
C	3.817862	-1.818090	0.717076
C	4.061233	-2.212774	-0.722509
C	2.322168	-1.985092	0.999504
C	3.400265	-1.195941	-1.631285
C	2.034913	-0.778504	-1.074958
C	1.870299	-1.165018	2.193880
C	1.097945	1.251719	-0.229605
C	-0.175073	0.805689	-0.225582
C	1.481950	2.656896	0.099966
C	-1.266255	1.677683	0.115550
C	0.304944	3.478454	0.499651
C	-0.965187	3.029054	0.480028
C	-2.618912	1.253822	0.129461
C	-3.087088	0.009299	-0.188113
C	-4.476745	-0.412458	-0.160420
C	-5.545044	0.430014	0.195536
C	-4.785817	-1.735867	-0.505952
C	-6.848551	-0.027845	0.206359
C	-6.089854	-2.205945	-0.498110
C	-7.125931	-1.349667	-0.140107
H	3.608498	-3.195038	-0.896702
H	4.133025	-0.781328	0.876553
H	2.152248	-3.041675	1.210335
H	4.034044	-0.308447	-1.678196
H	1.310478	-0.725900	-1.885856
H	0.814935	-1.352566	2.397889
H	2.022024	-0.101956	2.001766
H	5.876522	-2.774534	-0.365114
H	4.354354	-2.492552	2.446527
H	4.037225	-2.010374	-3.272903
H	2.502449	-1.057321	4.057847
H	-0.373855	-0.232998	-0.436347
H	2.260243	2.668061	0.868783
H	-1.779584	3.694185	0.745545
H	-3.331587	2.013866	0.428662
H	-2.387955	-0.759750	-0.492574
H	-0.105974	5.298649	0.967230
H	-5.359001	1.459870	0.466893
H	-3.983614	-2.407195	-0.785579
H	-7.665997	0.625331	0.480032
H	-6.303448	-3.233250	-0.768865
H	-8.488676	-2.673580	-0.371007
O	2.078864	3.235043	-1.087752
O	3.026821	4.213587	-0.689032
H	2.461907	4.960928	-0.445221

ENERGIES
SCF ENERGY: -1528.36032436
SUM OF ELECTRONIC AND ZERO-POINT ENERGIES: -1527.935520
SUM OF ELECTRONIC AND THERMAL ENERGIES: -1527.906712
SUM OF ELECTRONIC AND THERMAL ENTHALPIES: -1527.905768
SUM OF ELECTRONIC AND THERMAL FREE ENERGIES: -1527.997184

==> PET/RAF/C4/TS <==

53
XYZ-COORDINATES + ENERGIES

O	1.562892	-1.677397	-0.118677
O	5.478031	-2.181123	-1.116903
O	4.691573	-2.663396	1.472274
O	2.231305	0.529203	-0.467524
O	3.164162	-1.674683	-2.942511
O	2.819453	-1.620527	3.286790
O	0.695291	4.616151	1.167046
O	-8.395453	-1.819492	-0.191055
C	3.893132	-1.792011	0.686542
C	4.106347	-2.168359	-0.762727
C	2.406828	-1.979601	1.003359
C	3.409715	-1.152670	-1.646185
C	2.055277	-0.755273	-1.049998
C	1.975460	-1.177981	2.217729
C	1.125875	1.249149	-0.129646
C	-0.163160	0.778924	-0.159815
C	1.424112	2.593132	0.237706
C	-1.224615	1.616426	0.256229
C	0.354113	3.370725	0.746717
C	-0.943652	2.913702	0.716102
C	-2.611830	1.184312	0.257148
C	-3.070820	-0.012837	-0.153014
C	-4.464816	-0.450736	-0.150768
C	-5.535330	0.357480	0.261870
C	-4.757587	-1.747708	-0.584207
C	-6.834916	-0.111035	0.243119
C	-6.057760	-2.230885	-0.606711
C	-7.099808	-1.409305	-0.192159
H	3.662577	-3.154908	-0.935785
H	4.199897	-0.753467	0.850278
H	2.254313	-3.040257	1.207107
H	4.031811	-0.257699	-1.704750
H	1.312470	-0.702073	-1.843604
H	0.925796	-1.375093	2.441412
H	2.116794	-0.111376	2.038361
H	5.936819	-2.711027	-0.456467
H	4.476585	-2.481069	2.394959
H	4.010582	-1.942864	-3.313456
H	2.656850	-1.081634	4.064603
H	-0.361614	-0.239960	-0.448854
H	2.436634	2.832378	0.529389
H	-1.753014	3.550649	1.053297
H	-3.312906	1.921697	0.630511
H	-2.372829	-0.751960	-0.529423
H	-0.092711	5.093785	1.449600
H	-5.355953	1.368702	0.600395
H	-3.948754	-2.390063	-0.909140
H	-7.658257	0.514564	0.559828
H	-6.261982	-3.239766	-0.944888
H	-8.452381	-2.723703	-0.516046
O	1.669500	3.469541	-1.440125
O	2.540890	4.520926	-1.209409
H	1.968712	5.228841	-0.884296

ENERGIES
SCF ENERGY: -1528.33835833
SUM OF ELECTRONIC AND ZERO-POINT ENERGIES: -1527.915515
SUM OF ELECTRONIC AND THERMAL ENERGIES: -1527.886484
SUM OF ELECTRONIC AND THERMAL ENTHALPIES: -1527.885540
SUM OF ELECTRONIC AND THERMAL FREE ENERGIES: -1527.978034

==> PET/RAF/C4PP <==
53
XYZ-COORDINATES + ENERGIES

O	2.347352	-1.255698	0.002927
O	6.228862	-1.972394	-0.982575
O	5.509462	-1.965013	1.669997
O	3.012976	0.847368	-0.752119
O	3.883503	-1.761651	-2.817373
O	3.695675	-0.578361	3.305995
O	1.458728	5.167299	0.095718
O	-7.322389	-1.118531	-1.209482
C	4.695248	-1.243087	0.759279
C	4.867021	-1.878139	-0.602532
C	3.216565	-1.352362	1.141925
C	4.157850	-1.026010	-1.635452
C	2.818859	-0.517164	-1.089922
C	2.823484	-0.333667	2.196140
C	1.913167	1.629531	-0.516907
C	0.615225	1.143117	-0.463439
C	2.193555	2.980944	-0.324490
C	-0.440713	2.039326	-0.227744
C	1.145744	3.855906	-0.085582
C	-0.166476	3.398181	-0.038834
C	-1.830603	1.615149	-0.158133
C	-2.287788	0.337603	-0.327429
C	-3.637941	-0.080239	-0.262091
C	-4.742514	0.806523	0.000874
C	-3.939907	-1.468292	-0.470504
C	-6.011377	0.369732	0.049878
C	-5.195818	-1.948079	-0.419739
C	-6.370928	-1.064859	-0.161054
H	4.410229	-2.873642	-0.584974
H	5.012168	-0.195274	0.722902
H	3.062191	-2.354968	1.543038
H	4.785883	-0.161753	-1.859518
H	2.057207	-0.607074	-1.862384
H	1.779841	-0.475036	2.481394
H	2.965110	0.679481	1.817699
H	6.700140	-2.382179	-0.249387
H	5.319346	-1.615356	2.548902
H	4.717512	-2.119158	-3.137846
H	3.561120	0.100706	3.971305
H	0.423716	0.089006	-0.571840
H	3.212025	3.338941	-0.372467
H	-0.977945	4.092142	0.146936
H	-2.537784	2.408542	0.045512
H	-1.573704	-0.450502	-0.534866
H	0.654495	5.677733	0.232628
H	-4.549576	1.857926	0.161094
H	-3.117112	-2.142328	-0.674959
H	-6.832325	1.045744	0.239803
H	-5.407383	-3.000275	-0.567712
H	-7.326718	-2.012344	-1.571938
O	-6.979944	-1.635681	1.014285
O	-8.149980	-0.895968	1.332723
H	-8.748306	-1.135982	0.610450

ENERGIES
SCF ENERGY: -1528.36122697
SUM OF ELECTRONIC AND ZERO-POINT ENERGIES: -1527.937217
SUM OF ELECTRONIC AND THERMAL ENERGIES: -1527.908417
SUM OF ELECTRONIC AND THERMAL ENTHALPIES: -1527.907473
SUM OF ELECTRONIC AND THERMAL FREE ENERGIES: -1527.998589

==> PET/RAF/C4PP/TS <==
53
XYZ-COORDINATES + ENERGIES

O	2.316911	-1.252080	-0.119602
O	6.261664	-1.858434	-0.913780
O	5.380675	-2.063306	1.681865
O	3.010714	0.906836	-0.664457
O	4.024642	-1.534202	-2.867634
O	3.450240	-0.831225	3.304009
O	1.387366	5.152582	0.396444
O	-7.599296	-1.397831	-0.841397
C	4.613735	-1.280399	0.780213
C	4.877973	-1.806693	-0.612897
C	3.116034	-1.434428	1.059490
C	4.220892	-0.885645	-1.620928
C	2.846932	-0.427047	-1.119831
C	2.642018	-0.509947	2.165763
C	1.895196	1.668049	-0.435100
C	0.598445	1.176586	-0.485521
C	2.156979	3.002899	-0.133958
C	-0.470701	2.050732	-0.240392
C	1.093570	3.855702	0.109893
C	-0.217506	3.390928	0.057653

C	-1.868480	1.616507	-0.270059
C	-2.304506	0.367141	-0.526387
C	-3.686034	-0.062724	-0.557854
C	-4.784346	0.810934	-0.380136
C	-3.956134	-1.430510	-0.801281
C	-6.068086	0.344133	-0.391060
C	-5.233736	-1.915672	-0.832241
C	-6.323672	-1.054620	-0.511832
H	4.435339	-2.805039	-0.699150
H	4.916788	-0.229979	0.844745
H	2.952435	-2.468040	1.367071
H	4.851094	-0.002866	-1.743511
H	2.133950	-0.458331	-1.941798
H	1.585923	-0.688828	2.373672
H	2.786749	0.532271	1.878566
H	6.693862	-2.315410	-0.184413
H	5.129147	-1.787236	2.571540
H	4.880381	-1.859602	-3.164092
H	3.251143	-0.220424	4.017545
H	0.417916	0.133126	-0.682708
H	3.174477	3.365580	-0.103328
H	-1.042570	4.066486	0.251715
H	-2.586253	2.399518	-0.056887
H	-1.583836	-0.417549	-0.724636
H	0.573617	5.648499	0.530328
H	-4.617108	1.869836	-0.244924
H	-3.125045	-2.099122	-0.985896
H	-6.912036	1.004858	-0.253989
H	-5.431562	-2.961236	-1.033036
H	-7.648070	-2.352024	-0.970074
O	-6.401414	-1.494978	1.378697
O	-7.515297	-0.857021	1.882438
H	-8.257424	-1.372785	1.539392

ENERGIES
SCF ENERGY: -1528.33493923
SUM OF ELECTRONIC AND ZERO-POINT ENERGIES: -1527.912619
SUM OF ELECTRONIC AND THERMAL ENERGIES: -1527.883740
SUM OF ELECTRONIC AND THERMAL ENTHALPIES: -1527.882796
SUM OF ELECTRONIC AND THERMAL FREE ENERGIES: -1527.974189

==> PET/RAF/C5 <==
53
XYZ-COORDINATES + ENERGIES

O	1.758062	-1.595078	-0.022044
O	5.633556	-2.217998	-1.087853
O	4.965315	-2.246054	1.578027
O	2.354883	0.525932	-0.779753
O	3.255819	-2.042379	-2.877779
O	3.150878	-0.902494	3.259674
O	0.678938	4.167853	1.311034
O	-8.046188	-2.487957	0.136328
C	4.119602	-1.533445	0.688802
C	4.277746	-2.154964	-0.680861
C	2.651355	-1.674517	1.099873
C	3.531482	-1.310761	-1.693904
C	2.189463	-0.840543	-1.121480
C	2.258322	-0.666505	2.164329
C	1.244419	1.281024	-0.487719
C	-0.053029	0.728380	-0.420332
C	1.475329	2.598801	-0.245900
C	-1.169523	1.560504	-0.100525
C	0.367863	3.528532	0.093486
C	-0.978825	2.886014	0.140453
C	-2.532937	1.019747	-0.019498
C	-2.880483	-0.266086	-0.153429
C	-4.239248	-0.812326	-0.070538
C	-5.380771	-0.030998	0.157511
C	-4.414798	-2.189239	-0.227800
C	-6.638460	-0.601270	0.224034
C	-5.671577	-2.775199	-0.163003
C	-6.787094	-1.978045	0.063226
H	3.842051	-3.159866	-0.662229
H	4.415305	-0.479364	0.654481
H	2.525822	-2.680826	1.501588
H	4.134113	-0.429121	-1.919809
H	1.418634	-0.952720	-1.881616
H	1.221283	-0.825754	2.464863
H	2.378528	0.350638	1.789140
H	6.127861	-2.621453	-0.366425
H	4.787502	-1.905169	2.462870
H	4.092987	-2.376127	-3.215236
H	3.028951	-0.217047	3.920929
H	-0.200158	-0.323525	-0.590004
H	2.475112	3.006208	-0.285445
H	-1.817041	3.531666	0.369939
H	-3.294990	1.765758	0.173832
H	-2.116504	-1.012268	-0.340307
H	-0.128031	4.560314	1.662107
H	-5.292655	1.039294	0.284082
H	-3.549395	-2.815197	-0.406061
H	-7.516809	0.004981	0.398790
H	-5.783749	-3.845511	-0.288162
H	-8.019329	-3.441579	0.009534
O	0.408374	4.511940	-0.969831
O	-0.532837	5.534064	-0.660387
H	0.043096	6.229405	-0.316541

ENERGIES
SCF ENERGY: -1528.34639086
SUM OF ELECTRONIC AND ZERO-POINT ENERGIES: -1527.923416
SUM OF ELECTRONIC AND THERMAL ENERGIES: -1527.894332
SUM OF ELECTRONIC AND THERMAL ENTHALPIES: -1527.893388
SUM OF ELECTRONIC AND THERMAL FREE ENERGIES: -1527.985679

==> PET/RAF/C5PP <==
53
XYZ-COORDINATES + ENERGIES

O	2.152187	-1.247837	-0.004181
O	5.981921	-2.228890	-0.976130
O	5.270083	-2.113257	1.676677
O	2.962876	0.784792	-0.808291
O	3.634400	-1.938241	-2.806898
O	3.545665	-0.591168	3.284189
O	1.727833	5.217579	-0.014281
O	-7.787333	-0.633707	0.522214
C	4.498793	-1.366154	0.748597
C	4.628942	-2.045112	-0.596615
C	3.016999	-1.377364	1.134625
C	3.966897	-1.182924	-1.652509
C	2.669457	-0.569004	-1.115171
C	2.688061	-0.317204	2.169697
C	1.924639	1.648218	-0.575854
C	0.592509	1.260390	-0.519178
C	2.304075	2.976337	-0.397787
C	-0.388871	2.232508	-0.288247
C	1.323191	3.928458	-0.176541
C	-0.020651	3.567806	-0.127372
C	-1.818067	1.900761	-0.193214
C	-2.325090	0.670703	-0.051049
C	-3.750444	0.331999	0.051936
C	-4.778330	1.317414	-0.084362
C	-4.086476	-0.964697	0.303858
C	-6.122817	0.970270	0.068929
C	-5.492524	-1.431116	0.418283
C	-6.488126	-0.322309	0.328915
H	4.115299	-3.011685	-0.550800
H	4.877580	-0.340525	0.685935
H	2.802693	-2.361126	1.554637
H	4.647785	-0.369627	-1.910353
H	1.900125	-0.622470	-1.883200
H	1.638214	-0.390604	2.457867
H	2.888321	0.678374	1.771829
H	6.429928	-2.648808	-0.234157
H	5.099254	-1.734191	2.547335
H	4.440312	-2.360249	-3.120611
H	3.449194	0.105604	3.937669
H	0.314497	0.226842	-0.643173
H	3.346136	3.257513	-0.451434
H	-0.779477	4.321500	0.047343

H	-2.480053	2.758326	-0.219058
H	-1.656807	-0.180830	0.014093
H	0.961142	5.783728	0.118232
H	-4.526106	2.341343	-0.309216
H	-3.319394	-1.718753	0.419537
H	-6.897423	1.720933	-0.014554
H	-7.882035	-1.594909	0.489013
H	-5.646553	-2.011901	1.335614
O	-5.675588	-2.383093	-0.675636
O	-6.880091	-3.112934	-0.379480
H	-7.231262	-3.248316	-1.269676

ENERGIES
SCF ENERGY: -1528.34895534
SUM OF ELECTRONIC AND ZERO-POINT ENERGIES: -1527.924559
SUM OF ELECTRONIC AND THERMAL ENERGIES: -1527.895611
SUM OF ELECTRONIC AND THERMAL ENTHALPIES: -1527.894666
SUM OF ELECTRONIC AND THERMAL FREE ENERGIES: -1527.986882

==> PET/RAF/C6 <==
53
XYZ-COORDINATES + ENERGIES

O	2.046686	-1.497607	-0.132944
O	5.990286	-1.566389	-1.140216
O	5.279087	-1.977084	1.483386
O	2.433376	0.748332	-0.627045
O	3.627911	-1.473684	-2.969115
O	3.298154	-1.050041	3.245766
O	0.444214	4.714130	0.850313
O	-7.873361	-2.792015	0.143343
C	4.374409	-1.265749	0.653262
C	4.629312	-1.705647	-0.771295
C	2.924170	-1.616508	0.998020
C	3.807787	-0.845150	-1.709969
C	2.415856	-0.583328	-1.125589
C	2.398168	-0.795289	2.161002
C	1.249635	1.345100	-0.308343
C	0.022785	0.720641	-0.327237
C	1.403052	2.711048	0.096671
C	-1.140027	1.428891	0.009828
C	0.317757	3.443941	0.400021
C	-1.072487	2.905825	0.258237
C	-2.441739	0.856187	0.098954
C	-2.780268	-0.431156	-0.188326
C	-4.111117	-1.010561	-0.092493
C	-5.250059	-0.295485	0.317099
C	-4.282049	-2.360584	-0.427235
C	-6.490099	-0.900646	0.389841
C	-5.521645	-2.978177	-0.358624
C	-6.630323	-2.246338	0.052696
H	4.314287	-2.749626	-0.875733
H	4.548375	-0.188465	0.747014
H	2.906322	-2.671977	1.272570
H	4.313853	0.114614	-1.829777
H	1.668814	-0.674498	-1.911854
H	1.384252	-1.110668	2.412014
H	2.398292	0.265766	1.907816
H	6.515355	-1.996553	-0.457013
H	5.045839	-1.763591	2.394893
H	4.500430	-1.676790	-3.320607
H	3.069040	-0.485863	3.988137
H	-0.042128	-0.334201	-0.540745
H	2.395881	3.133726	0.164076
H	-3.220082	1.538594	0.421116
H	-2.012747	-1.116168	-0.528761
H	-0.407562	5.158529	0.755619
H	-5.171840	0.749948	0.581458
H	-3.422978	-2.935845	-0.748676
H	-7.363080	-0.345289	0.704880
H	-5.627500	-4.023401	-0.624328
H	-7.842256	-3.718198	-0.116223
H	-1.676466	3.185317	1.127665
O	-1.732576	3.509972	-0.895629
O	-1.956495	4.891979	-0.569802
H	-1.671090	5.321806	-1.387116

ENERGIES
SCF ENERGY: -1528.35669535
SUM OF ELECTRONIC AND ZERO-POINT ENERGIES: -1527.932832
SUM OF ELECTRONIC AND THERMAL ENERGIES: -1527.903725
SUM OF ELECTRONIC AND THERMAL ENTHALPIES: -1527.902780
SUM OF ELECTRONIC AND THERMAL FREE ENERGIES: -1527.995864

==> PET/RAF/C6/TS <==
53
XYZ-COORDINATES + ENERGIES

O	2.014360	-1.508644	-0.100121
O	5.922119	-1.614257	-1.239663
O	5.297064	-2.008132	1.408153
O	2.405895	0.731124	-0.611899
O	3.499327	-1.509619	-2.987301
O	3.385228	-1.070202	3.232946
O	0.443993	4.663545	0.972865
O	-7.905086	-2.751279	0.086818
C	4.369855	-1.294139	0.606031
C	4.573592	-1.740970	-0.824559
C	2.929970	-1.635164	1.000145
C	3.727717	-0.877250	-1.738322
C	2.358451	-0.602620	-1.107850
C	2.449512	-0.810761	2.180413
C	1.245791	1.342005	-0.242224
C	0.002570	0.717100	-0.244057
C	1.405557	2.674889	0.170489
C	-1.127776	1.430728	0.150912
C	0.298821	3.387097	0.549128
C	-1.009143	2.820426	0.461311
C	-2.462720	0.862647	0.226603
C	-2.803386	-0.395308	-0.108894
C	-4.141421	-0.977961	-0.043749
C	-5.273880	-0.281255	0.405688
C	-4.313187	-2.303976	-0.453798
C	-6.516518	-0.883764	0.443381
C	-5.555412	-2.920602	-0.421271
C	-6.660727	-2.207743	0.029190
H	4.246661	-2.782642	-0.914595
H	4.553094	-0.217511	0.689670
H	2.914007	-2.690782	1.273991
H	4.236780	0.077972	-1.879697
H	1.586869	-0.689265	-1.870145
H	1.443083	-1.119994	2.466910
H	2.447344	0.250288	1.927026
H	6.466684	-2.046355	-0.573095
H	5.093911	-1.792463	2.326411
H	4.357333	-1.721543	-3.368129
H	3.184244	-0.506370	3.983723
H	-0.077465	-0.327768	-0.495408
H	2.388766	3.122882	0.184887
H	-3.225277	1.546625	0.579123
H	-2.038314	-1.071904	-0.473002
H	-0.415091	5.102607	0.935766
H	-5.189285	0.746855	0.729500
H	-3.455526	-2.862398	-0.807263
H	-7.387669	-0.344004	0.788758
H	-5.665523	-3.948540	-0.745520
H	-7.879134	-3.660975	-0.226549
H	-1.793387	3.275919	1.052678
O	-1.680096	3.626993	-1.123758
O	-1.622250	5.010510	-0.931884
H	-0.862521	5.278778	-1.465684

ENERGIES
SCF ENERGY: -1528.33970559
SUM OF ELECTRONIC AND ZERO-POINT ENERGIES: -1527.916998
SUM OF ELECTRONIC AND THERMAL ENERGIES: -1527.887958
SUM OF ELECTRONIC AND THERMAL ENTHALPIES: -1527.887014
SUM OF ELECTRONIC AND THERMAL FREE ENERGIES: -1527.979932

==> PET/RAF/C6PP <==
53
XYZ-COORDINATES + ENERGIES

O	2.008820	-1.149338	-0.197597
O	5.906380	-1.794715	-1.173580
O	5.127360	-2.078665	1.445948
O	2.723668	1.015888	-0.692737
O	3.592614	-1.366468	-3.020860
O	3.284877	-0.877275	3.190403
O	1.223611	5.278471	0.482297
O	-7.923066	-1.128629	-0.466037
C	4.338315	-1.251114	0.605298
C	4.536644	-1.731093	-0.815287
C	2.850699	-1.390442	0.940195
C	3.855628	-0.764322	-1.763087
C	2.513636	-0.297695	-1.187915
C	2.437970	-0.500407	2.098060
C	1.633799	1.792486	-0.400109
C	0.329217	1.321055	-0.388514
C	1.931907	3.120648	-0.101213
C	-0.714582	2.209566	-0.082848
C	0.894963	3.988443	0.201266
C	-0.423429	3.545906	0.209324
C	-2.110713	1.798101	-0.042877
C	-2.576760	0.530647	-0.234675
C	-3.936886	0.125282	-0.204084
C	-5.028984	0.994701	-0.024743
C	-4.186885	-1.354951	-0.302029
C	-6.319648	0.544207	-0.066505
C	-5.612381	-1.743624	-0.504779
C	-6.597546	-0.842829	-0.346434
H	4.073358	-2.718428	-0.918265
H	4.662657	-0.208845	0.694395
H	2.679784	-2.431928	1.215530
H	4.497676	0.110078	-1.884219
H	1.766088	-0.283989	-1.979079
H	1.387924	-0.669656	2.341293
H	2.590212	0.549309	1.843523
H	6.357960	-2.287163	-0.479935
H	4.915374	-1.833083	2.354594
H	4.426539	-1.704533	-3.361860
H	3.117108	-0.297686	3.937189
H	0.123560	0.282845	-0.587675
H	2.955226	3.467362	-0.119743
H	-1.226299	4.233386	0.448452
H	-2.812829	2.594479	0.169455
H	-1.865981	-0.266063	-0.420647
H	0.425579	5.785430	0.661557
H	-4.851998	2.050549	0.126157
H	-3.548664	-1.798603	-1.069020
H	-7.156106	1.214868	0.067271
H	-5.833526	-2.772515	-0.760316
H	-8.035742	-2.066716	-0.651942
O	-3.678437	-1.863912	0.973593
O	-3.650955	-3.283748	0.892769
H	-4.456653	-3.529367	1.365431

ENERGIES
SCF ENERGY: -1528.35171045
SUM OF ELECTRONIC AND ZERO-POINT ENERGIES: -1527.927777
SUM OF ELECTRONIC AND THERMAL ENERGIES: -1527.898777
SUM OF ELECTRONIC AND THERMAL ENTHALPIES: -1527.897833
SUM OF ELECTRONIC AND THERMAL FREE ENERGIES: -1527.989777
